# Randomised trial comparing weight loss through lifestyle and GLP-1 receptor agonist therapy in people with MASLD

**DOI:** 10.1016/j.jhepr.2025.101363

**Published:** 2025-02-21

**Authors:** Ahmad Moolla, Toryn Poolman, Nantia Othonos, Jiawen Dong, Kieran Smith, Thomas Cornfield, Sarah White, David W. Ray, Sofia Mouchti, Ferenc E. Mózes, Helena Thomaides-Brears, Stefan Neubauer, Jeremy F. Cobbold, Leanne Hodson, Jeremy W. Tomlinson

**Affiliations:** 1 Oxford Centre for Diabetes Endocrinology & Metabolism (OCDEM), NIHR Oxford Biomedical Research Centre, University of Oxford, Churchill Hospital, Oxford, UK; 2 Structural and Molecular Biology, Faculty of Life Sciences, University College London, London, UK; 3 NIHR Oxford Health Biomedical Research Centre, University of Oxford, Oxford, UK; 4 Oxford Kavli Centre for Nanoscience Discovery, University of Oxford, Oxford, UK; 5 Perspectum, Gemini One, Oxford, UK; 6 Oxford Centre for Clinical Magnetic Resonance Research (OCMR), John Radcliffe Hospital, University of Oxford, Oxford, UK; 7 Department of Gastroenterology and Hepatology, Oxford University Hospitals, Oxford, UK; 8 NIHR Oxford Biomedical Research Centre, Oxford, UK

**Keywords:** MASLD, GLP-1, Liraglutide, Weight loss

## Abstract

**Background & Aims:**

Glucagon-like peptide 1 receptor agonist (GLP-1RA) therapies deliver histological benefit in people with metabolic dysfunction-associated steatotic liver disease (MASLD). Multiple mechanisms may be important including weight loss, improved glycaemic control and putative direct tissue-specific actions. Following cessation of GLP1-RA therapy, weight regain is common. To dissect the mechanisms underpinning their benefits, we conducted a prospective, randomised, experimental medicine study in people with MASLD, comparing GLP-1RA treatment (liraglutide) to matched lifestyle-induced weight loss and assessed the impact of treatment withdrawal.

**Methods:**

Twenty-nine participants with MASLD, without type 2 diabetes underwent metabolic phenotyping including measurement *de novo* lipogenesis (DNL), liver magnetic resonance imaging, body composition, adipose tissue RNA sequencing, circulating proteome, and stool microbiome analysis. Participants were randomised to lifestyle (∼500 kcal energy deficit) or GLP1-RA treatment for 12 weeks, after which investigations were repeated, and treatment stopped; investigations were also repeated 12 weeks after treatment withdrawal.

**Results:**

Matched weight loss was achieved in both arms. Body composition changes, reductions in alanine aminotransferase, liver steatosis, and disease activity were similar following both treatments. GLP-1RA treatment, but not lifestyle, improved glucose handling, fasting lipids, and significantly deceased DNL. The subcutaneous adipose transcriptome, circulating proteome profile and stool microbiome were not different between groups after treatment. However, 12 weeks after GLP1-RA (but not lifestyle) withdrawal, circulating MMP-10, IL10RB, FGF-23, and Flt3L were elevated, alongside dysregulated adipose gene expression.

**Conclusions:**

Although matched weight loss through lifestyle or GLP-1RA have comparable effects on hepatic steatosis, GLP-1RA treatment had additional metabolic benefits on glucose homeostasis, lipid profiles, and DNL. However, GLP-1RA withdrawal may adversely impact the circulating proteome, adipose tissue gene expression, and the stool microbiome, predisposing to weight regain.

**Impact and implications:**

Weight loss, through either lifestyle intervention or pharmacotherapy with GLP-1RA has an equally beneficial impact on the liver, and both strategies should be considered in the management of people with MASLD. GLP-1RA therapy may have additional benefits to improve glucose homeostasis even in the absence of pre-existing type 2 diabetes. Further research is needed to explore the differential impact of treatment withdrawal and the resultant metabolic consequences.

**Clinical Trials Registration:**

This study is registered at EudraCT (2016-002045-36).

## Highlights

• Matched weight loss with either lifestyle or GLP-1RA therapy cause similar reductions in liver fat content.
• GLP-1RA therapy, but not lifestyle-induced weight loss, improves glucose homeostasis in the absence of type 2 diabetes.
• Withdrawal of interventions differentially impacts glucose homeostasis, the plasma proteome, and adipose gene expression.

## Graphical abstract

**Figure:**
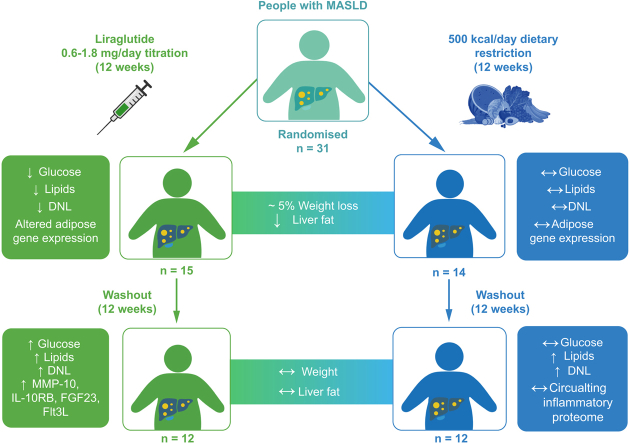

## Introduction

Current estimates suggest that non-alcoholic fatty liver disease (NAFLD), more recently termed metabolic dysfunction-associated steatotic liver disease (MASLD), affects up to 30% of unselected populations with a prevalence that has risen rapidly over the past 15 years.^1^ MASLD is now the most common chronic liver disease, the second leading worldwide indication for liver transplantation (and the leading indication in some populations^2^), and is tightly associated with type 2 diabetes (T2D) and obesity.^3^ Although the more advanced stages are associated with liver-specific morbidity and mortality, there is increased cardiovascular risk across all stages and severity of MASLD which is highest in people with advanced fibrosis.[4], [5], [6] Despite the significant disease burden, only a single agent has been granted FDA approval^7^ and the current mainstays of treatment for MASLD remain weight reduction, achieved through dietary and lifestyle measures, alongside aggressive cardiovascular risk reduction.^8^

Current evidence suggests that agents with the ability to induce significant weight loss and offer cardiovascular risk protection have clinical utility in the treatment of people with MASLD. Glucagon-like peptide 1 (GLP-1) receptor agonists (GLP-1RA) have a range of metabolically beneficial actions, acting peripherally and centrally to reduce appetite and promote satiety resulting in significant weight loss.^9^ In addition, they enhance glucose-dependent insulin secretion from the pancreatic islets, improving glycaemic control in people with T2D. They also have important anti-inflammatory actions.^10^ The cardioprotective impact of GLP-1RA therapy is now established with several large cardiovascular outcome trials demonstrating clear benefits and together strongly suggest a class effect of GLP-1RA therapy.^11^^,^^12^ GLP-1RA therapy is safe and well tolerated in most people and has a good side-effect profile suggesting no major concerns with respect to long-term use.

Specifically in the context of MASLD, the GLP-1RA, liraglutide, decreases alanine aminotransferase (ALT) in a dose-dependent manner^13^ as well as limiting hepatic *de novo* lipogenesis (DNL),^14^ a critical driver of hepatic triacylglycerol (TAG) accumulation. The LEAN study was the first to demonstrate (using histological endpoints) the ability of liraglutide to resolve metabolic dysfunction-associated steatohepatitis (MASH) in people with biopsy-proven disease, with and without T2D.^15^ More recently, semaglutide, a more potent GLP1-RA, has achieved resolution of MASH in up to 59% of people and this was associated with a 13% reduction in weight.^16^ However, in people with MASLD-cirrhosis, it failed to improve fibrosis, but was safe and well tolerated and conveyed significant metabolic improvements (weight reduction, improved glucose control, and lipid profiles).^17^ Although not yet in the peer reviewed literature, there is emerging evidence to suggest that semaglutide may have a beneficial impact on liver fibrosis in the phase III ESSENCE trial.^18^

Over a 12-week intervention period, liraglutide (doses up to 3 mg daily) induces weight loss of ∼5%,^19^ however, it remains unclear as to whether the benefits conferred by GLP-1RA therapy in people with MASLD (and without co-existent T2D) are solely a result of weight loss. Weight loss is associated with significant improvements in MASLD and other metabolic variables, but it is plausible that there are additional benefits (over and above the magnitude of weight loss) mediated by GLP-1RA therapy. We have therefore, undertaken a prospective, randomised, controlled study in people with MASLD (but without T2D to limit the potential confounding impact of alterations in glycaemic control) to determine if matched weight loss (achieved through lifestyle intervention) has comparable effects to GLP-1RA therapy in people with MASLD. Treatment with pharmacotherapy for weight loss is often administered for a limited and defined time, and following cessation, weight regain is frequent.^20^ We have therefore included an exploration of mechanisms that may differ following treatment withdrawal that may have relevance for weight regain.

## Patients and methods

We conducted a single-centre, open-label, prospective, randomised phase IIa clinical trial to evaluate the effects of the GLP-1RA, liraglutide, compared with lifestyle-induced weight loss in people with a diagnosis of progressed MASLD over a period of 24 weeks. The clinical study protocol received full ethical approval from the East of England – Cambridgeshire and Hertfordshire Research Ethics Committee (REC ref. 16/EE/0403), and the National Health Service (NHS) Health Research Authority. The trial was sponsored and monitored by the Clinical Trials and Research Governance research support unit at University of Oxford, was registered as a Clinical Trial of an Investigational Medicinal Product with the UK Medicines and Healthcare Products Regulatory Agency and was registered with the European Clinical Trials Database (EudraCT Number 2016-002045-36). All participants provided informed written consent before participation.

### Clinical protocol

Individuals aged 18–75 years with a diagnosis of progressed MASLD, defined as steatosis on imaging or histology along with ALT >19 IU/L for females or >30 IU/L for males, or liver biopsy showing MASH or liver fibrosis, or vibration-controlled transient elastography (FibroScan, Echosens, Paris, France) liver stiffness measurement >8 kPa. Liver biopsy histological scoring and liver stiffness measurements are presented in Table S1. People with an established diagnosis of T2D were excluded to remove any confounding effects of concomitant antidiabetic medications or alterations in glycaemic control.

Participants underwent baseline investigations including anthropometric and blood pressure measurements, fasting blood tests, assessment of DNL using deuterated water (see below), and a subcutaneous abdominal adipose tissue biopsy. Aspiration of adipocytes (∼1 g of tissue) was achieved using a needle and syringe and liposuction following administration of local anaesthetic. Samples were snap frozen in liquid nitrogen before being stored at -80 °C before RNA extraction and RNA sequencing.

Following the completion of the investigations in the fasting state, a prolonged (180 min) oral glucose tolerance test (OGTT) was performed. At the start of the OGTT, a microdialysis catheter (CMA Microdialysis, Solna, Sweden) was inserted under local anaesthetic (2 ml, 1% lidocaine) into the subcutaneous adipose tissue, 5–10 cm lateral to the umbilicus to allow sampling of adipose tissue interstitial fluid. Using a microdialysis pump (CMA 106), the microdialysate solution (physiological sterile saline solution) was introduced into the catheter (perfusion rate = 0.3 μl/min) and samples collected at 30-min intervals until the completion of the OGTT. Serum sampling at 30-min intervals was performed alongside the microdialysis. Because of issues with equipment availability, microdialysis was only performed in a subset of participants (n = 18). The oral glucose load comprised 75 g glucose in addition to 0.5 g ^13^C-glucose (CK Gas Ltd., Newtown Unthank, UK) to allow measurement of glucose oxidation through ^13^CO_2_ generation (see below).

Body composition analysis was performed using dual-energy X-ray absorptiometry (DXA) (GE Lunar iDXA, GE Healthcare, Chalfont Saint Giles, UK). All participants completed the abbreviated International Physical Activity Questionnaire (IPAQ). Data were summarised and are presented as ‘physical activity’ per week.

### Magnetic resonance spectroscopy and LiverMultiScan

All magnetic resonance studies were performed at the University of Oxford Centre for Clinical Magnetic Resonance Research (OCMR) using a 3 Tesla system (TIM Trio, Siemens Healthineers, Erlangen, Germany). Detailed methods are included within the Supplementary data.

Participants were then randomised by members of the investigative team using a random number block randomisation tool to either a lifestyle intervention program provided by a commercial weight management organisation (Slimming World, Alfreton, UK) or to liraglutide treatment (starting dose 0.6 mg/day, titrated over a 3-week period to 1.8 mg/day) (Novo Nordisk, Copenhagen, Denmark) with the aim of achieving matched weight loss (estimated at 5%) across both interventional arms over a 12-week period. The lifestyle intervention included an approximate 500 kcal/day deficit, decreased consumption of fat (to <30% of dietary calorie intake) alongside increased fibre intake, principally through increased consumption of vegetables and fruit. No specific advice with regards to physical activity or exercise was provided. Study interventions were commenced within 1-week of baseline assessments. Interim visits at weeks 1, 4, and 8 assessed compliance, blood test monitoring and adverse drug effects. Those who suffered side effects and were not able to tolerate a 1.8 mg dose of liraglutide had a dose reduction to 1.2 mg daily (n = 2). Liraglutide injections were given before the OGTT on the study days. All biological samples collected were processed and stored at -80 °C (where applicable) in line with all relevant ethically agreed and regulatory protocols.

After 12 weeks of intervention, all the investigations described above were repeated. Treatments were then withdrawn (GLP-1RA and lifestyle advice) and then after a further 12 weeks (24 weeks after treatment initiation) a further set of investigations was repeated (Fig. S1).

### Stable isotope measurements

#### *De novo* lipogenesis

Hepatic DNL was assessed based on the incorporation of deuterium from ^2^H_2_O in plasma water (Finnigan GasBench-II, ThermoFisher Scientific, Altrincham, UK) into VLDL-TG palmitate using gas chromatography-mass spectroscopy with monitoring ions with mass-to-charge ratios (*m/z*) of 270 (M+0) and 271 (M+1).^21^

#### Glucose oxidation

To assess glucose oxidation through ^13^CO_2_ generation, a modified OGTT incorporating ^13^C-glucose was performed. Breath samples were collected at 30-min intervals throughout the duration of the test. ^13^C/^12^C ratios in breath samples and the relative rate of whole-body fatty acid oxidation was calculated as previously described.^22^ The rate of expiration of ^13^CO_2_ in breath was calculated by multiplying the estimated VCO_2_ by the enrichment of breath CO_2_.

### Biochemical and targeted proteomic analysis

Fasting biochemical bloods (full blood count, renal function, liver chemistry, and lipid profile) were analysed through the NHS clinical laboratories at Oxford University Hospitals NHS Foundation Trust. Serum insulin was measured using a commercially available ELISA (Mercodia, Uppsala, Sweden) according to the manufacturer protocol and insulin resistance (homeostatic model assessment for insulin resistance [HOMA-IR]) was calculated using established criteria. Concentrations of non-esterified fatty acids (NEFAs), triglyceride, glucose, total cholesterol, and HDL cholesterol were measured using commercially available kits on an ILAB600/ILAB650 clinical analyser (Instrumentation Laboratory UK, Warrington, UK). Targeted proteomic analysis was performed on samples acquired in the fasting state using a commercially available platform (O-link, Uppsala, Sweden). Microdialysis samples were analysed for glucose and glycerol levels, using a mobile photometric enzyme-kinetic analyser (CMA ISCUS Flex, Solna, Sweden).

### RNA extraction and RNA sequencing

Total RNA from adipose tissue biopsies was extracted using the Tri-Reagent system (Sigma-Aldrich, Dorset, UK). Detailed methods are presented in the Supplementary data.

### Microbiome and analysis and 16S rRNA sequencing

Stool samples were collected and analysed at baseline, 12 weeks, and 24 weeks. DNA extraction and 16S sequencing was undertaken by Omega Bioservices (Norcross, GA, USA). Detailed methods are presented in the Supplementary data.

### Statistical analysis

The primary outcome for the study was the change in hepatic steatosis (baseline to 12 weeks) using magnetic resonance spectroscopy (MRS) and the study was powered to detect a 20% relative change over a 12-week intervention in both treatment arms (power = 0.8, α = 0.05). Where appropriate, AUC was calculated using the trapezoid method. Statistical analyses were undertaken using R version 4.3 (R Foundation for Statistical Computing, Vienna, Austria). Paired *t* tests (or their non-parametric equivalents if data were not normally distributed) were used to compare outcomes before and after intervention. Unpaired *t* tests (or non-parametric equivalents) were used to compare treatment arms. Where multiple comparisons were made over time, repeated measures ANOVA analyses were used. The statistical significance level for all results was set as *p* <0.05. Circulating protein levels obtained from the targeted proteomic analysis were normalised and log_2_ applied. Intensity values were analysed using LIMMA, utilising the duplicateCorrelation function to account for the related nature of the samples.

## Results

**Fig. 1:**
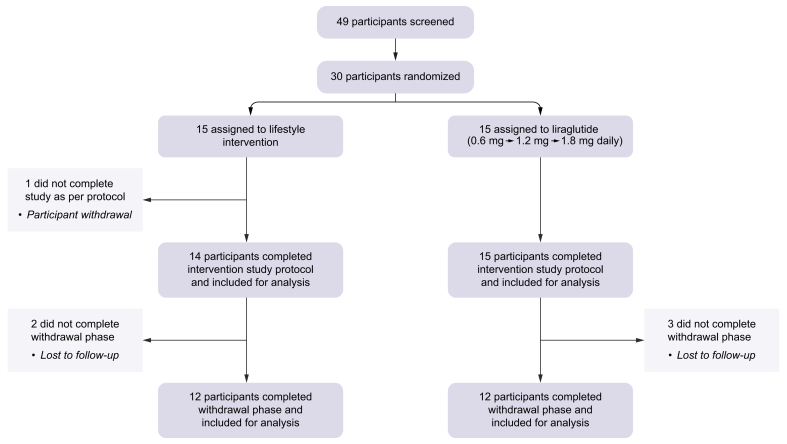
Consort diagram summarising participant recruitment and analysis.

Thirty-three of 49 volunteers who successfully passed screening were enrolled into the study between January 2017 and April 2019. Thirty-one participants (two declined after screening) were randomised to either the lifestyle or liraglutide intervention. At baseline, no participants had impaired fasting glucose, but five of 29 had impaired glucose tolerance (Table 1). Twenty-nine participants (n = 14 lifestyle, n = 15 liraglutide) completed the 12-week intervention required for evaluation of the primary study outcome. In the liraglutide treatment arm 13/15 people were able to tolerate the highest dose (1.8 mg/day) and two required a dose reduction to 1.2 mg daily. Twenty-four participants (n = 12 in both arms) were followed up at 24 weeks to evaluate the legacy effects of interventions (Fig. 1). Both groups were well-matched at baseline, except for total cholesterol and HDL cholesterol which were lower in the liraglutide-treated group. Baseline demographic and anthropometric data are presented in Table 1.

**Table 1:** Baseline parameters in 29 people with MASLD and the impact of a 12-week intervention with either lifestyle or GLP-1RA therapy with liraglutide.

	Lifestyle (n = 14)		GLP-1RA, liraglutide (n = 15)	
	Baseline	12 weeks	Baseline	12 weeks
Demographics				
Age (years)	48 ± 4		48 ± 4	
Sex (% male)	50.0		53.3	
Weight, body composition and blood pressure				
Weight (kg)	104.2 ± 5.8	99.5 ± 5.4§	106.9 ± 5.6	101.7 ± 5.4§
BMI (kg/m^2^)	36.4 ± 1.5	34.8 ± 1.4§	35.7 ± 1.7	33.9 ± 1.6§
Body fat (%)	45.0 ± 2.1	43.3 ± 2.2§	43.3 ± 2.5	41.9 ± 2.5§
Total fat mass (kg)	45.2 ± 3.4	41.9 ± 3.4§	45.3 ± 4.2	41.9 ± 4.0§
Total lean mass (kg)	54.7 ± 3.3	54.1 ± 3.1	57.6 ± 3.0	56.2 ± 3.0§
Estimated visceral adipose mass (kg)	2.4 ± 0.3	2.1 ± 0.2‡	2.4 ± 0.3	2.0 ± 0.3§
Blood pressure (mmHg) systolic (S)/diastolic (D)	S: 136 ± 4 D: 80 ± 2	S: 123 ± 2§ D: 71 ± 2§	S: 133 ± 3 D: 83 ± 2	S: 126 ± 3‡ D: 79 ± 2∗
Physical activity				
Physical activity (IPAQ), time sitting (h/day)	7.9 ± 1.0	7.6 ± 1.2	6.5 ± 1.1	6.8 ± 1.2
Physical activity (IPAQ), total weekly activity (h)	12.2 ± 4.0	9.2 ± 2.5	15.3 ± 5.1	14.2 ± 4.9
Fasting lipid profile				
Triglyceride (mmol/L)	1.5 ± 0.2	1.3 ± 0.2	1.6 ± 0.2	1.2 ± 0.1‡
Total cholesterol (mmol/L)	5.0 ± 0.3	4.7 ± 0.2	4.1 ± 0.2∗	3.8 ± 0.2†^,^‡
HDL cholesterol (mmol/L)	1.3 ± 0.1	1.2 ± 0.1§	1.0 ± 0.05†	1.0 ± 0.05∗
non-HDL cholesterol (mmol/L)	3.7 ± 0.2	3.5 ± 0.2	3.1 ± 0.2	2.8 ± 0.2∗^,^‡
NEFA (μmol/L)	517 ± 49	459 ± 43	521 ± 51	441 ± 41
Insulin and glucose metabolism				
HbA1c (mmol/mol)	37 ± 1	36 ± 1	39 ± 1	35 ± 1§
Fasting glucose (mmol/l)	4.9 ± 0.1	4.9 ± 0.1	5.0 ± 0.2	4.6 ± 0.1
Number with impaired glucose tolerance (%)	2 (13)	0	3 (21)	0
Glucose AUC over 180 min OGTT (mmol/L.min)	7.6 ± 0.5	7.4 ± 0.4	7.3 ± 0.4	6.0 ± 0.2†^,^§
Fasting insulin (pmol/L)	112 ± 18	91 ± 19‡	136 ± 24	125 ± 15
Insulin AUC over 180 min OGTT (pmol/L.min)	898 ± 111	815 ± 92	809 ± 104	795 ± 64
HOMA-IR	4.2 ± 0.8	3.3 ± 0.7‡	5.0 ± 0.8	4.3 ± 0.5
Glucose oxidation (breath^13^CO_2_ AUC) over 180 min OGTT (mmol/kg lean mass)	2.4 × 10^-4^ ± 2.0 × 10^-5^	2.3 × 10^-4^ ± 1.6 × ^-5^	2.2 × 10^-4^ ± 1.6 × 10^-5^	2.1 × 10^-4^ ± 1.8 × 10^-5^
Adipose tissue				
Subcutaneous microdialysis interstitial glucose AUC over 180 min OGTT (mmol/L.h)¶	11.7 ± 0.9	10.4 ± 1.3	12.0 ± 1.7	9.0 ± 1.4‡
Subcutaneous microdialysis interstitial glycerol AUC over 180 min OGTT (μmol/L.h)¶	547 ± 66	573 ± 69	566 ± 71	507 ± 35
ADIPO-IR (fasting NEFA × fasting insulin) (mmol/L.h × μmol/L.h)	60.3 ± 13.6	46.1 ± 12.2	77.1 ± 17.5	55.5 ± 8.8
Liver biochemistry				
ALT (IU/L)	61 ± 8	47 ± 8§	58 ± 8	46 ± 6‡
AST (IU/L)	37 ± 4	32 ± 5	36 ± 3	31 ± 4
Magnetic resonance spectroscopy and imaging				
MRS hepatic steatosis (%)	21.1 ± 2.4	13.6 ± 2.1§	24.3 ± 2.3	18.1 ± 1.9§
Intrahepatic iron (mg/g dry weight)	1.2 ± 0.1	1.1 ± 0.1§	1.2 ± 0.1	1.2 ± 0.1
Iron corrected T1 (cT1) (ms)	861 ± 23	813 ± 23§	872 ± 29	827 ± 24§
Non-invasive markers of liver fibrosis				
FIB-4 FIB-4 risk stratification; low/indeterminate/high (n)	1.1 ± 0.2 10/4/0	1.1 ± 0.1 10/4/0	0.9 ± 0.1 12/3/0	0.9 ± 0.1 13/2/0
NFS NFS risk stratification; low/indeterminate/high (n)	-1.3 ± 0.3 6/8/0	-1.2 ± 0.3 7/7/0	-1.9 ± 0.4 10/5/0	-1.9 ± 0.3 9/6/0
Hepatic DNL				
Fasting DNL (%)	12.9 ± 1.9	10.7 ± 2.1	15.2 ± 2.5	9.7 ± 1.9‡
DNL AUC over 180 min OGTT (%.min)	13.0 ± 1.9	11.5 ± 1.9	14.4 ± 2.1	10.2 ± 1.8‡

Data presented are mean ± SEM unless otherwise stated.

ALT, alanine aminotransferase; AST, aspartate aminotransferase; AUC, area under curve; DNL, *de novo* lipogenesis; FIB-4, fibrosis-4; GLP-1RA, glucagon-like peptide 1 receptor agonist; HOMA-IR, homeostatic model assessment for insulin resistance; IPAQ, International Physical Activity Questionnaire; MASLD, metabolic dysfunction-associated steatotic liver disease; MRS, magnetic resonance spectroscopy; NEFA, non-esterified fatty acid; NFS, NAFLD fibrosis score; OGTT, oral glucose tolerance test.

∗ *p* <0.05.

† *p* <0.01 lifestyle *vs.* GLP-1RA.

‡ *p* <0.05.

§ *p* <0.01 baseline *vs.* 12 weeks within group, paired or unpaired *t* tests where appropriate.

¶ Data from n = 10 (liraglutide) and n = 8 (lifestyle).

### Impact of 12 weeks of active intervention

#### Body weight, composition, physical activity, and blood pressure

**Fig. 2:**
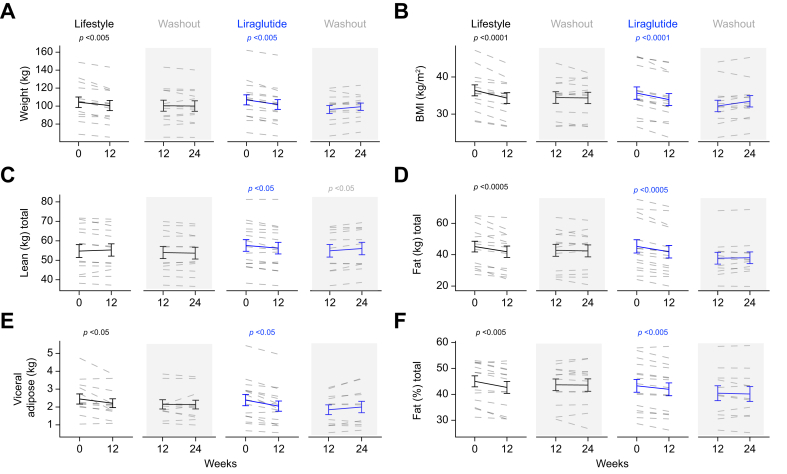
Weight and body composition changes following lifestyle or GLP-1RA induced weight loss. Lifestyle intervention and treatment with GLP-1RA for 12 weeks are associated with significant, and comparable weight loss (A and B). Total and regional fat mass are decreased and there is a small reduction in lean mass in people treated with GLP1-RA (C–F). With the exception of a modest increase in lean mass following GLP-1RA withdrawal, body composition changes are maintained 12 weeks after interventional withdrawal (paired *t* test). GLP-1RA, glucagon-like peptide 1 receptor agonist.

Both interventions achieved significant weight loss; the magnitude of weight loss was similar in both arms (-4.7 ± 1.0 *vs.* -5.2 ± 0.7 kg, lifestyle *vs*. liraglutide, *p* = 0.63) (Table 1) (Fig. 2A and B). Total weight loss was principally attributable to a reduction in total fat mass; lean mass did not change significantly in the lifestyle group but decreased in the GLP1-RA group (Table 1) (Fig. 2C and D). Percent fat mass and estimated visceral fat mass decreased in both groups, and there were no significant differences between the two arms (Table 1) (Fig. 2E and F). Systolic blood pressure improved in both groups, and diastolic blood pressure improved in the lifestyle group only, which remained lower than the GLP-1RA group at 12 weeks (Table 2). Physical activity as assessed by IPAQ data did not change in either arm (Table 1).

**Table 2:** Differentially expressed genes in abdominal subcutaneous adipose tissue biopsies after lifestyle and GLP-1RA treatment.

Gene	*p* value	Adjusted *p* value	LogFC	Gene description
**Differentially expressed genes (baseline *vs.* 12 weeks of GLP-1RA treatment)**				
*HTRA2*	8.62 × 10^-7^	0.017	2.08	HtrA serine peptidase 2
*CPA6*	3.95 × 10^-6^	0.031	2.30	Carboxypeptidase A6
*MDGA1*	4.99 × 10^-6^	0.031	2.01	MAM domain containing glycosylphosphatidylinositol anchor 1
*CDC42EP1*	6.48 × 10^-6^	0.031	2.63	CDC42 effector protein 1
*LINC00881*	1.17 × 10^-5^	0.036	1.80	Long intergenic non-protein coding RNA 881
*ZFP62*	1.46 × 10^-5^	0.036	2.17	ZFP62 zinc finger protein
*KRT73-AS1*	1.54 × 10^-5^	0.036	1.79	KRT73 antisense RNA 1
*RALGPS2*	1.61 × 10^-5^	0.036	2.04	Ral GEF with PH domain and SH3 binding motif 2
*SP8*	1.70 × 10^-5^	0.036	1.86	Sp8 transcription factor
*APCS*	2.34 × 10^-5^	0.045	1.72	Amyloid P component, serum
*SVBP*	2.59 × 10^-5^	0.045	2.09	Small vasohibin binding protein
*PTCD2*	3.07 × 10^-5^	0.049	2.32	Pentatricopeptide repeat domain 2
*SIGLEC7*	3.31 × 10^-5^	0.049	1.89	Sialic acid-binding Ig like lectin 7
*SYCP1*	3.65 × 10^-5^	0.050	2.08	Synaptonemal complex protein 1

**Differentially expressed genes (GLP-1RA *vs.* lifestyle 12 weeks after treatment withdrawal)**				
*GAB4*	1.10 × 10^-7^	0.0013	2.31	GRB2 associated binding protein family member 4
*CAMK2B*	1.33 × 10^-7^	0.013	3.01	Calcium/calmodulin-dependent protein kinase II beta
*VWDE*	1.32 × 10^-6^	0.0084	2.32	von Willebrand factor D and EGF domains
*KLHL28*	0.000005	0.026	2.20	Kelch like family member 28

#### Glucose and lipid homeostasis

The study only recruited people without a diagnosis of T2D and all people had normal fasting glucose levels at baseline, and this did not change significantly in either arm after 12 weeks of intervention. Impaired glucose tolerance resolved in five of five participants irrespective of their treatment arm. Glycated haemoglobin decreased significantly in the GLP-1RA treatment arm, but not in those undergoing lifestyle intervention (Table 1). Consistent with these data, AUC glucose across the prolonged OGTT was reduced by GLP-1RA and not by lifestyle (Table 1). In addition, subcutaneous adipose tissue interstitial glucose across the OGTT as measured by microdialysis decreased significantly in the GLP-1RA group only (Table 1). Insulin sensitivity increased as assessed by fasting insulin levels, HOMA-IR improved only in the lifestyle group. Insulin concentrations across the prolonged OGTT did not change significantly in either group, nor did glucose oxidation (as measured by ^13^CO_2_ appearance in breath) (Table 1). In the GLP-1RA treated group only, fasting triglycerides, total cholesterol, and non-HDL cholesterol all decreased. Lifestyle was associated with a small but significant decrease in HDL cholesterol (Table 1). Although fasting NEFA levels decreased in both treatment groups, this failed to reach statistical significance. There was also no significant change in adipose tissue insulin resistance as measured by adipo-IR. Adipose tissue interstitial glycerol levels across the OGTT did not change in either treatment arm (Table 1).

#### Liver biochemistry, hepatic steatosis, and DNL

**Fig. 3:**
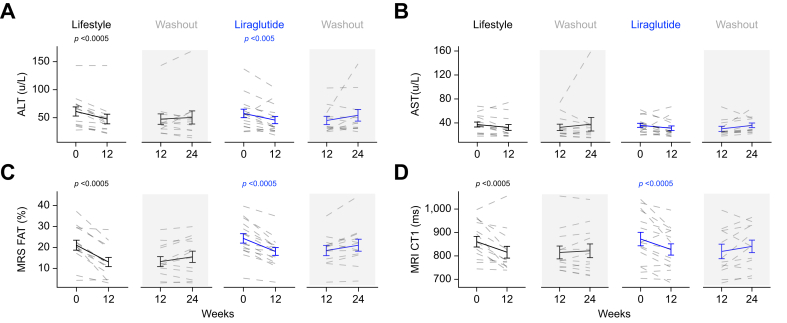
Changes in liver biochemistry, imaging and *de novo* lipogenesis following lifestyle or GLP-1RA induced weight loss. Lifestyle intervention and treatment with GLP-1RA for 12-weeks are associated with similar reductions in ALT, but not AST (A and B). Both interventions significantly decreased liver fat content as well as cT1 values on MR scanning (C and D) (paired *t* test). ALT, alanine aminotransferase; AST, aspartate aminotransferase; cT1, iron corrected T1; GLP-1RA, glucagon-like peptide 1 receptor agonist; MRS, magnetic resonance spectroscopy.

ALT levels decreased in both treatment arms. There were no significant differences between the two interventions; there were no significant changes in aspartate aminotransferase (AST) (Table 1) (Fig. 3A and B). MRS demonstrated similar reductions in hepatic steatosis in both treatment groups (Table 1) (Fig. 3C). Liver iron content decreased in the lifestyle group only, and iron corrected T1 (cT1), a biomarker of hepatic fibroinflammation, decreased to a similar extent in both groups (Table 1) (Fig. 3D, Fig. S2). Although both fasting DNL and DNL measured across the prolonged OGTT decreased in both arms, this only reached statistical significance in the GLP-1RA-treated group (Table 1).

#### Circulating proinflammatory cytokine profiles

Ninety-two proinflammatory cytokines were measured in serum samples from all participants and there were no significant differences between the two groups at baseline. When adjusted for multiple comparisons, there were no significant changes in circulating proinflammatory cytokines levels after GLP-1RA treatment or lifestyle intervention. There were no significant differences between the two arms of the study at the end of the intervention period (Supplementary data).

#### RNA sequencing of abdominal subcutaneous adipose

RNA-sequencing analysis of subcutaneous adipose tissue revealed no significant differentially expressed genes (DEGs) at baseline between the two treatment groups. After 12 weeks of intervention, there remained no significant DEGs between groups. However, 14 DEGs were identified comparing samples before and after GLP-1RA treatment (Table 2). There were no DEGs before and after the lifestyle intervention.

#### Microbiome

At baseline, analysis of the 16S microbiome RNA sequencing demonstrated no significant differences in absolute or relative abundances of microbiome phyla, notably Bacteroidetes and Firmicutes. In addition, the Firmicutes:Bacteroidetes ratio was similar in both groups. After 12 weeks of intervention with either lifestyle or liraglutide, there remained no significant differences between the groups (Fig. S3).

### Mixed effects modelling

To determine the primary drivers of improvement in hepatic steatosis, mixed effects modelling was performed including age, sex, intervention, and change in weight. Weight reduction was the only variable included in the model that had a significant impact on the reduction in MRS measured liver fat content (*p* = 0.001).

### Impact of treatment withdrawal

All treatments were withdrawn after 12 weeks. Twenty-four individuals (12 in each arm) were followed up at 24 weeks for assessment of legacy effects of either intervention. A separate analysis was then undertaken including individuals who completed all investigation visits (baseline, end of treatment, and 12 weeks post-treatment); participants with missing data were excluded from the analysis.

Following GLP1-RA withdrawal, weight increased by 1.3 ± 0.9 kg between the end of treatment and 12 weeks post-treatment, with a significant increase in lean mass (Table 3). After lifestyle withdrawal, there was a further modest reduction in weight (-0.4 ± 1.0 kg). However, when comparing weight change between arms from baseline to 12 weeks post-treatment and end of treatment to 12 weeks post-treatment, there were no significant differences. In addition, there were no significant changes in body composition when comparing the two treatment arms (Table 3) (Fig. 2).

**Table 3:** The impact of treatment withdrawal (lifestyle or GLP-1A) following 12 weeks of intervention in people with MASLD.

	Lifestyle (n = 12)		GLP-1RA, liraglutide (n = 12)	
	End of treatment	12-weeks post-intervention withdrawal	End of treatment	12-weeks post-intervention withdrawal
Weight, body composition and blood pressure				
Weight (kg)	100.4 ± 6.1	100.0 ± 5.9	97.9 ± 4.4	99.3 ± 4.1
BMI (kg/m^2^)	34.5 ± 1.6	34.4 ± 1.5	33.0 ± 1.6	33.4 ± 1.5
Body fat (%)	43.7 ± 2.2	43.6 ± 2.4	40.4 ± 2.9	40.1 ± 2.9
Total fat mass (kg)	42.8 ± 3.8	42.4 ± 3.8	37.8 ± 3.7	38.0 ± 3.7
Total lean mass (kg)	54.0 ± 3.1	53.7 ± 3.0	54.9 ± 3.2	56.0 ± 3.2§
Estimated visceral adipose mass (kg)	2.1 ± 0.3	2.1 ± 0.3	1.8 ± 0.3	2.0 ± 0.3
Blood pressure (mmHg) systolic (S)/diastolic (D)	S: 121 ± 3 D: 71 ± 3	S: 128 ± 2§ D: 77 ± 3‡	S: 127 ± 4 D: 79 ± 2	S: 133 ± 4 D: 81 ± 2
Physical activity				
Physical activity (IPAQ), time sitting (h/day)	9.3 ± 1.6	8.3 ± 1.8	5.1 ± 0.6 a	4.7 ± 0.7
Physical activity (IPAQ), total weekly activity (h)	9.6 ± 3.0	6.5 ± 1.5	17.6 ± 5.8	19.5 ± 7.0
Fasting lipid profile				
Triglyceride (mmol/L)	1.2 ± 0.2	1.5 ± 0.4	1.2 ± 0.1	1.4 ± 0.1
Total cholesterol (mmol/L)	4.6 ± 0.2	5.0 ± 0.3§	3.9 ± 0.2	4.2 ± 0.2§
HDL cholesterol (mmol/L)	1.2 ± 0.1	1.3 ± 0.1‡	1.0 ± 0.1	1.1 ± 0.1
non-HDL cholesterol (mmol/L)	3.4 ± 0.2	3.7 ± 0.3‡	2.9 ± 0.2	3.1 ± 0.2‡
NEFA (μmol/L)	437 ± 34	389 ± 47	459 ± 47	407 ± 55
Insulin and glucose metabolism				
HbA1c (mmol/mol)	36 ± 1	37 ± 1	35 ± 1	37 ± 1.0‡
Fasting glucose (mmol/L)	4.9 ± 0.1	4.7 ± 0.2‡	4.5 ± 0.1∗	5.1 ± 0.2‡
Number with impaired glucose tolerance (%)	0	0	1 (8)	0
Glucose AUC over 180 min OGTT (mmol/L.min)	7.5 ± 0.4	7.1 ± 0.4	6.1 ± 0.3†	7.1 ± 0.5
Fasting insulin (pmol/L)	92 ± 22	110 ± 31	127 ± 17	127 ± 27
Insulin AUC over 180min OGTT (pmol/L.min)	727 ± 110	685 ± 154	833 ± 75	682 ± 129
HOMA-IR	3.3 ± 0.8	3.7 ± 1.1	4.2 ± 0.6	4.4 ± 1.1
Glucose oxidation (Breath^13^CO_2_ AUC) over 180 min OGTT (mmol/kg lean mass)	2.2 × 10^-4^ ± 1.5 × 10^-5^	2.7 × 10^-4^ ± 1.8 × 10^-5^§	2.1 × 10^-4^ ± 2.0 × 10^-5^	2.4 × 10^-4^ ± 2.0 × 10^-5^
Liver biochemistry				
ALT (IU/L)	48 ± 9	50 ± 12	46 ± 7	54 ± 10
AST (IU/L)	33 ± 5	38 ± 11	31 ± 4	36 ± 4
Adipose tissue				
ADIPO-IR (fasting NEFA × fasting insulin) (mmol/L × pmol/L)	43.8 ± 14.8	54.8 ± 17.2	57.9 ± 10.2	64.2 ± 14.0
Magnetic resonance spectroscopy and imaging				
MRS hepatic steatosis (%)	14.0 ± 2.5	15.3 ± 2.9	18.5 ± 2.4	20.8 ± 3.1
Intrahepatic iron (mg/g dry weight)	1.1 ± 0.1	1.1 ± 0.1	1.3 ± 0.2	1.2 ± 0.1
Iron corrected T1 (cT1) (ms)	818 ± 30	821 ± 32	819 ± 30	845 ± 29
Non-invasive markers of liver fibrosis				
FIB-4 FIB-4 risk stratification; low/indeterminate/high (n)	1.1 ± 0.2 8/4/0	1.2 ± 0.2 8/4/0	0.9 ± 0.1 10/2/0	1.0 ± 0.1§ 10/2/0
NFS NFS risk stratification; low/indeterminate/high (n)	-1.0 ± 0.3 5/7/0	-1.0 ± 0.4 4/8/0	-2.0 ± 0.4 8/4/0	-2.0 ± 0.4 8/4/0
Hepatic DNL				
Fasting DNL (%)	9.4 ± 2.1	14.1 ± 2.6‡	9.2 ± 2.7	14.6 ± 2.3‡
DNL AUC over 180 min OGTT (%.min)	10.1 ± 2.3	16.2 ± 3.5‡	9.3 ± 2.4	13.3 ± 2.2

Data are presented as mean ± SEM unless otherwise stated.

ALT, alanine aminotransferase; AST, aspartate aminotransferase; AUC, area under curve; DNL, *de novo* lipogenesis; FIB-4, fibrosis-4; GLP-1RA, glucagon-like peptide 1 receptor agonist; HOMA-IR, homeostatic model assessment for insulin resistance; IPAQ, International Physical Activity Questionnaire; MRS, magnetic resonance spectroscopy; NEFA, non-esterified fatty acid; NFS, NAFLD fibrosis score; OGTT, oral glucose tolerance test.

∗ *p* <0.05.

† *p* <0.01 lifestyle *vs.* GLP-1RA.

‡ *p* <0.05.

§ *p* <0.01 end of treatment *vs.* 12-weeks post-treatment within group; paired or unpaired *t* tests where appropriate.

The changes in ALT, hepatic steatosis, and cT1 all persisted through 12 weeks of treatment withdrawal and there were no significant differences between treatment groups during this washout phase. However, fasting DNL in both groups, and DNL across the prolonged OGTT in the lifestyle group alone, significantly increased following treatment withdrawal (Table 3).

The reductions in glucose AUC across the OGTT and HbA1c that were observed following 12 weeks of GLP-1RA treatment, reversed completely following treatment withdrawal (Table 3). In addition, fasting glucose increased significantly in the GLP-1RA treated group while decreased in the lifestyle group. Impaired glucose tolerance recurred in one participant following GLP-1RA withdrawal. At 12 weeks post-treatment, there were no significant differences in glucose parameters between treatment arms. Similarly circulating NEFA did not change in either group and although adipo-IR increased in both groups, it did not reach statistical significance.

**Fig. 4:**
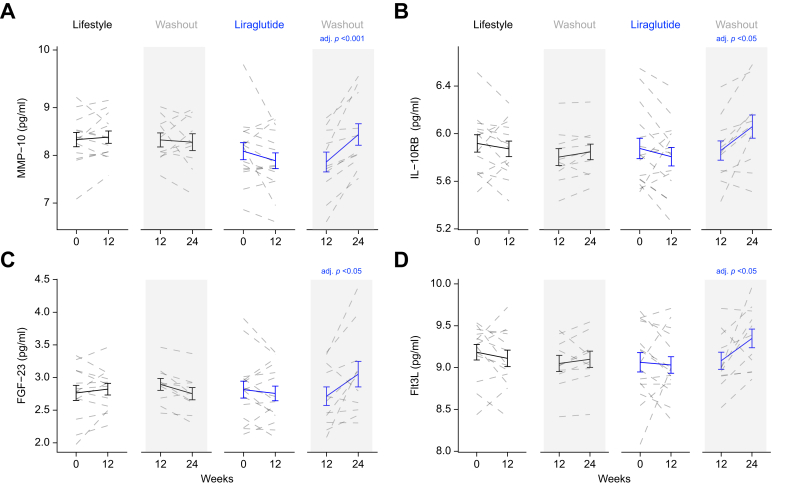
Circulating cytokine profiles following lifestyle- or GLP-1RA-induced weight loss. Lifestyle intervention and treatment with GLP-1RA for 12 weeks are not associated with changes in circulating proinflammatory cytokines. However, 12 weeks after treatment withdrawal, circulating levels of matrix metalloproteinase-10 (MMP-10) (A), interleukin-10 receptor B (IL-10RB) (B), fibroblast growth factor-23 (FGF-23) (C) and FMS-like tyrosine kinase 3 ligand (Flt3L) (D), increased significantly, only in those people who had been treated with GLP-1RA (paired *t* test). GLP-1RA, glucagon-like peptide 1 receptor agonist.

Circulating proinflammatory cytokines did not change after lifestyle withdrawal, however, after adjusting for multiple comparisons, four cytokines (matrix metalloproteinase-10 [MMP-10], interleukin-10 receptor B [IL-10RB], fibroblast growth factor 23 [FGF23], and FMS-like tyrosine kinase 3 ligand [Flt3L]) increased significantly after liraglutide withdrawal (Fig. 4) (Supplementary data).

Although there were no adipose tissue DEGs at baseline or after 12 weeks of intervention between groups, after treatment withdrawal, four DEGs were identified comparing the liraglutide and lifestyle-treated participants (Table 2).

Pooled analysis of the stool microbiome did not demonstrate robust changes. Although there was a trend to an increased abundance of Firmicutes and deceased Bacteroidetes after GLP-1RA withdrawal (with opposing effects in the lifestyle group), these did not achieve statistical significance. There was no difference in the Firmicutes:Bacteroidetes ratio between groups (Fig. S3).

## Discussion

We have demonstrated that over a 12-week intervention period, both lifestyle and GLP-1RA therapy with liraglutide achieved a comparable magnitude of weight loss, and this was associated with similar reductions in hepatic steatosis and fat mass. GLP-1RA had an additional impact to improve glucose homeostasis (in people without T2D) which was lost after treatment withdrawal. Significant reductions in DNL and improvements in fasting lipid profiles were only seen in the GLP-1RA-treated group. The beneficial impact of both interventions on the liver was maintained following the 12-week treatment washout period, although in people treated with GLP-1RA, treatment withdrawal was associated with changes in a small number of proinflammatory cytokines and transcriptional changes in adipose tissue and these may have important implications for potential weight regain.

The magnitude of weight loss with GLP-1RA therapy in this study was similar to that reported in the literature.^19^ Furthermore, the observed reduction in liver fat content (6.2%) in the GLP-1RA arm is similar to previous studies in people with MASLD,^23^^,^^24^ although this is not a consistent finding in all studies.^25^ Improvements in cT1 were significant, although lower than observed in a recent GLP-1RA/GIP trial in people with MASH, over a 52-week treatment period.^26^ The data with regards to lifestyle interventions are also comparable to the published literature. A meta-analysis of lifestyle interventions in people with MASLD identified weight loss of between 4% and14% with a corresponding reduction in liver fat of 4–10%.^27^ We observed a 7.5% reduction in liver fat content in the lifestyle arm.

To date, very few studies have tried to directly compare the impact of GLP-1RA therapy with matched weight loss. Two published studies have used higher doses of liraglutide (3 mg) with a 26-week intervention period. Immediately following treatment, and similar to our observations, the reductions in weight and liver fat content were similar, although of note, there was increased weight regain in the liraglutide-treated group compared with the lifestyle intervention arm.^28^^,^^29^

There is now an established body of evidence demonstrating the beneficial impact of GLP-1RA in people with MASLD to decrease hepatic steatosis and resolve MASH^15^^,^^16^^,^^30^^,^^31^ and also to improve cardiovascular and also liver outcomes.^32^^,^^33^ However, the impact on liver fibrosis is less clear.^16^^,^^17^ The improvements in lipid profiles that we observed specifically in the GLP-1RA-treated people may offer some explanation as to the mechanisms underpinning reductions in cardiovascular risk that are likely to occur in people with MASLD treated with these drugs (accepting that a dedicated cardiovascular outcome studies in people with MASLD, treated with GLP-1RA have not been performed).

A putative direct impact of GLP-1RA therapy on the liver remains controversial. GLP-1 has a well-established role to regulate glucose-sensitive insulin secretion from the pancreatic islet, but GLP-1 receptors are also expressed in extra-pancreatic tissues. Although some studies have suggested that the GLP-1 receptor (at the mRNA level) may be expressed in human liver, more recent studies using a variety of robust methodologies have concluded that there is no expression in human hepatocytes.^34^ This lack of functional receptor activity may explain some of the variability of the impact of GLP-1RA to limit hepatic DNL which has been reported in some, but not all studies.^14^^,^^35^ In the current study, DNL decreased in both arms, but only reached statistical significance in the GLP-1RA-treated people. It is possible that this additional benefit with GLP-1RA, over and above weight loss could reflect improved glucose handling, decreasing substrate availability (glucose) for DNL. Glucose handling worsened following GLP-1RA withdrawal, and this might explain the rebound and the observed increase in DNL, however, in the lifestyle group, after treatment withdrawal, DNL also increased despite no change in glucose homeostasis. It remains possible that the mechanisms underpinning these changes differ between treatment arms, but that in both situations, treatment withdrawal may lead to increased DNL that may fuel re-accumulation of hepatic TAG.

Weight regain remains an important and challenging clinical problem. It is often overlooked in clinical studies and occurs after all weight loss interventions. In the same way that mechanisms of weight loss differ across all interventions (as does their relative efficacy), the trajectory of weight regain may also differ. The mechanisms that contribute to weight regain are multiple and complex. There are changes in lipolytic function in adipose tissue^36^ alongside changes in lipid oxidation, alterations in circulating regulators of appetite^37^ as well as emerging data suggesting that changes in the microbiome may play a role in the predisposition to weight regain.^38^ In this study, we observed differences in circulating proinflammatory cytokines, the adipose tissue transcriptome, and the gut microbiome that only became apparent after treatment withdrawal.

The clinical significance of the changes that we observed in the circulating cytokine profiles after GLP-1RA withdrawal remain to be determined. MMP-10 (stomelysin-2) is highly expressed in epithelial cells. Although mice with genetic deletion to not display a clear metabolic phenotype,^39^ MMP-10 has been implicated in the development of hepatocellular carcinoma.^40^^,^^41^ FGF-23 is principally regarded as a bone-derived regulator of phosphate and vitamin D homeostasis. Positive associations between circulating FGF-23 levels and hepatic steatosis, adiposity, and circulating hyperlipidaemia have been described^42^^,^^43^ and therefore following weight loss intervention withdrawal, a differential impact on circulating FGF-23 levels could have a metabolic impact. Flt3L has complex immune regulatory functions and its role in metabolic disease has not been explored. A single study has reported lower circulating levels in people with diabetic retinopathy (compared with healthy control participants),^44^ with evidence for decreasing aqueous humour levels after treatment for diabetic macular oedema using anti-VEGF therapy.^45^ Similarly, the role of IL-10RB in regulating metabolic phenotype is largely unknown. However, both circulating and cerebrospinal fluid levels are positively associated with BMI^46^^,^^47^ although whether this is a cause or consequence cannot be determined.

Our analysis of the microbiome was compromised by limited sample numbers necessitating a pooled analysis. However, following intervention withdrawal, we did observe a trend towards increased Firmicutes and decreased Bacteroidetes, specifically following GLP-1RA (but not lifestyle) treatment. There is evidence from rodent studies to suggest that GLP-1RA treatment can impact the microbiome,^48^ but clinical studies are sparse and although some have suggested that the impact of liraglutide may be modest (in comparison with metformin),^49^ others have demonstrated more robust effects.^50^ However, in the studies published to date, the specific impact of weight loss, as distinct from effects on blood glucose control, cannot be disentangled. Of interest, many studies have reported that an increased Firmicutes:Bacteroidetes ratio (as we observed following GLP-1RA withdrawal) is associated with obesity, and this may have implications for future weight regain in this group.^51^

Lifestyle intervention had no impact on subcutaneous abdominal adipose tissue gene expression profiles. However, GLP-1RA treatment was associated with a small number of gene expression changes (Table 2). Most of the identified DEGs have not been examined in the context of MASLD, however, in people with MASLD, altered natural killer cell expression of Siglec-7 (Sialic acid-binding immunoglobulin-like lectin-7), that is associated with compromised NK cell function has been described.^52^ Four DEGs between treatment arms were identified following treatment withdrawal at 24 weeks. Calcium/calmodulin-dependent protein kinase II beta (CAMK2B) was significantly increased (>8-fold) in the liraglutide treatment group. Although the precise impact of this is not clear, it is interesting to note that genetic inhibition of CAMK2 signalling specifically in adipose tissue (through conditional genetic deletion of other CAM2K subunits, delta and gamma) improves adipose insulin sensitivity and glucose tolerance^53^ and this may contribute to the rebound effects that we observed after GLP-1RA withdrawal.

The current study does have some limitations. Although this was a relatively small study over a short duration, it incorporated a dedicated washout period (which is often omitted in clinical experimental studies) alongside deep metabolic phenotyping to provide a multisystem assessment of the impact of intervention and treatment withdrawal. There was no placebo-treated group to act as comparator to both GLP-RA and lifestyle intervention. Although established liver and cardiometabolic parameters were assessed, the outcome measures did not include a liver biopsy which continues to be the gold standard assessment of MASLD stage and severity (including MASH and fibrosis). Over this duration, it is unlikely that significant changes in liver fibrosis would have been observed and therefore non-invasive assessments to measure fibrosis specifically, were not included. The study only included people without T2D, but this was felt to be a crucial element of the study design in attempting to control for variables (other than weight) that might influence MASLD progression. In addition, although the formal lifestyle intervention was withdrawn, it is entirely plausible that elements of the lifestyle programme were continued by the participants during the washout phase (contrasting with liraglutide withdrawal), and this could explain differences in the propensity to weight regain and changes in metabolic variables measured at the end of the washout phase.

In conclusion, we have demonstrated that weight loss is the major contributor to the improvement in hepatic steatosis. We did observe differences in glucose handling, lipid profiles and DNL that might favour GLP-1RA treatment and perhaps explain improvements in cardiovascular risk, but this could be off-set by the impact of treatment withdrawal. This also brings into focus the clinical impact of intermittent use of GLP-1RA treatment, but the long-term consequences of this need to be explored in larger scale studies of longer duration.

The data from this study advocate that weight loss should remain the primary focus to improve outcomes for people with MASLD and that pharmacotherapy, including the use of GLP1-RAs may be important tools in trying to achieve this, as recommended in recent international practice guidance.^54^ With either intervention, there were significant benefits both metabolically and specifically to the liver. Importantly, our observations suggest that in contrast to ongoing intervention, treatment withdrawal may have a differential impact on individuals with MASLD. The precise consequences of this need to be explored in more detail, but there could be clinical implications for weight maintenance or regain.

## Abbreviations

ALT, alanine aminotransferase; AST, aspartate aminotransferase; cT1, iron corrected T1; DEGs, differentially expressed genes; DNL, *de novo* lipogenesis; DXA, dual-energy X-ray absorptiometry; GLP-1, glucagon-like peptide 1; GLP-1RA, glucagon-like peptide 1 receptor agonist; HOMA-IR, homeostatic model assessment for insulin resistance; IPAQ, International Physical Activity Questionnaire; *m*/*z*, mass-to-charge ratio; MASH, metabolic dysfunction-associated steatohepatitis; MASLD, metabolic dysfunction-associated steatotic liver disease; MRS, magnetic resonance spectroscopy; NAFLD, non-alcoholic fatty liver disease; NEFA, non-esterified fatty acid; NHS, National Health Service; OGTT, oral glucose tolerance test; T2D, type 2 diabetes; TAG, triacylglycerol.

## Financial support

Medical Research Council (MR/P011462/1 to JWT; MR/W019000/1 and MR/V034049/1 to DRW); National Institute for Health and Care Research (NIHR) Oxford Biomedical Research Centre (to JWT and DWR) and NIHR Oxford Health Biomedical Research Centre (NIHR203316); Novo Nordisk Fellowship, University of Oxford (to AM); British Heart Foundation (FS/15/56/31645 and FS/SBSRF/21/31013 senior fellowship to LH). FEM was funded by a Sir Henry Dale Fellowship awarded jointly by the 10.13039/100010269Wellcome Trust and the 10.13039/501100000288Royal Society (221805/Z/20/Z). The views expressed are those of the authors and not necessarily those of the National Health Service, the NIHR, or the Department of Health.

## Authors’ contributions

Conceptualisation: SN, JFC, JWT. Methodology: AM, TP, SM, FEM, HT-B, LH. Formal analysis: AM, TP, TC, SM, FEM, HT-B, JD, KS. Investigation: AM, NO, SW. Resources: LH, DWR, JFC, SN, JWT. Data curation: AM, TP, HT-B, LH, JWT. Writing – original draft: AM, JWT. Writing – review and editing, all authors. Supervision: SN, LH, JWT. Project administration: AA, LH, JWT. Funding acquisition, AA, LH, DWR, SN, JWT.

## Data availability statement

Data, materials, and methods from this study are included in the Supplementary material. The sequencing data and associated analysis have been deposited under project ID PRJEB66353 and are available on GitHub at https://github.com/toryn13/LILIpaper. Additional details can be made available from the corresponding author upon request.

## Conflicts of interest

JWT has been an advisory board member for Novo Nordisk and a member of a data and safety monitoring committee for Novartis; JFC has been an advisory board member for Novo Nordisk and Intercept; consultancy for Alnylam; FEM, LH, TC, JD, and KS have nothing to declare. SM and HTB are employees at Perspectum, the company that developed LiverMultiScan. HTB is also a shareholder at Perspectum. SN is a founder and shareholder of Perspectum.

Please refer to the accompanying ICMJE disclosure forms for further details.

## References

[1] Younossi Z.M., Golabi P., Paik J.M. (2023). The global epidemiology of nonalcoholic fatty liver disease (NAFLD) and nonalcoholic steatohepatitis (NASH): a systematic review. Hepatology.

[2] Stepanova M., Kabbara K., Mohess D. (2022). Nonalcoholic steatohepatitis is the most common indication for liver transplantation among the elderly: data from the United States Scientific Registry of Transplant Recipients. Hepatol Commun.

[3] Paik J.M., Golabi P., Younossi Y. (2020). Changes in the global burden of chronic liver diseases from 2012 to 2017: the growing impact of NAFLD. Hepatology.

[4] Dulai P.S., Singh S., Patel J. (2017). Increased risk of mortality by fibrosis stage in nonalcoholic fatty liver disease: systematic review and meta-analysis. Hepatology.

[5] de Avila L., Henry L., Paik J.M. (2023). Nonalcoholic fatty liver disease is independently associated with higher all-cause and cause-specific mortality. Clin Gastroenterol Hepatol.

[6] Taylor R.S., Taylor R.J., Bayliss S. (2020). Association between fibrosis stage and outcomes of patients with nonalcoholic fatty liver disease: a systematic review and meta-analysis. Gastroenterology.

[7] Harrison S.A., Bedossa P., Guy C.D. (2024). A phase 3, randomized, controlled trial of resmetirom in NASH with liver fibrosis. N Engl J Med.

[8] Marjot T., Moolla A., Cobbold J.F. (2020). Nonalcoholic fatty liver disease in adults: current concepts in etiology, outcomes, and management. Endocr Rev.

[9] Ard J., Fitch A., Fruh S. (2021). Weight loss and maintenance related to the mechanism of action of glucagon-like peptide 1 receptor agonists. Adv Ther.

[10] Bray J.J.H., Foster-Davies H., Salem A. (2021). Glucagon-like peptide-1 receptor agonists improve biomarkers of inflammation and oxidative stress: a systematic review and meta-analysis of randomised controlled trials. Diabetes Obes Metab.

[11] Bethel M.A., Patel R.A., Merrill P. (2018). Cardiovascular outcomes with glucagon-like peptide-1 receptor agonists in patients with type 2 diabetes: a meta-analysis. Lancet Diabetes Endocrinol.

[12] Yoshiji S., Minamino H., Tanaka D. (2022). Effects of glucagon-like peptide-1 receptor agonists on cardiovascular and renal outcomes: a meta-analysis and meta-regression analysis. Diabetes Obes Metab.

[13] Armstrong M.J., Houlihan D.D., Rowe I.A. (2013). Safety and efficacy of liraglutide in patients with type 2 diabetes and elevated liver enzymes: individual patient data meta-analysis of the LEAD program. Aliment Pharmacol Ther.

[14] Armstrong M.J., Hull D., Guo K. (2016). Glucagon-like peptide 1 decreases lipotoxicity in non-alcoholic steatohepatitis. J Hepatol.

[15] Armstrong M.J., Gaunt P., Aithal G.P. (2016). Liraglutide safety and efficacy in patients with non-alcoholic steatohepatitis (LEAN): a multicentre, double-blind, randomised, placebo-controlled phase 2 study. Lancet.

[16] Newsome P.N., Buchholtz K., Cusi K. (2021). A placebo-controlled trial of subcutaneous semaglutide in nonalcoholic steatohepatitis. N Engl J Med.

[17] Loomba R., Abdelmalek M.F., Armstrong M.J. (2023). Semaglutide 2·4 mg once weekly in patients with non-alcoholic steatohepatitis-related cirrhosis: a randomised, placebo-controlled phase 2 trial. Lancet Gastroenterol Hepatol.

[18] Plainsboro N.J. ESSENCE Phase 3 trial results demonstrating statistically significant and superior improvements with semaglutide 2.4 mg in people with MASH presented at AASLD 2024 - The Liver Meeting. Novo Nordisk USA. https://www.novonordisk-us.com/media/news-archive/news-details.html?id=171986.

[19] Xie Z., Yang S., Deng W. (2022). Efficacy and safety of liraglutide and semaglutide on weight loss in people with obesity or overweight: a systematic review. Clin Epidemiol.

[20] Wilding J.P.H., Batterham R.L., Davies M. (2022). Weight regain and cardiometabolic effects after withdrawal of semaglutide: the STEP 1 trial extension. Diabetes Obes Metab.

[21] Pramfalk C., Pavlides M., Banerjee R. (2015). Sex-specific differences in hepatic fat oxidation and synthesis may explain the higher propensity for NAFLD in men. J Clin Endocrinol Metab.

[22] Parry S.A., Rosqvist F., Cornfield T. (2021). Oxidation of dietary linoleate occurs to a greater extent than dietary palmitate in vivo in humans. Clin Nutr.

[23] Smits M.M., Tonneijck L., Muskiet M.H. (2016). Twelve week liraglutide or sitagliptin does not affect hepatic fat in type 2 diabetes: a randomised placebo-controlled trial. Diabetologia.

[24] Cuthbertson D.J., Irwin A., Gardner C.J. (2012). Improved glycaemia correlates with liver fat reduction in obese, type 2 diabetes, patients given glucagon-like peptide-1 (GLP-1) receptor agonists. PLoS One.

[25] Tang A., Rabasa-Lhoret R., Castel H. (2015). Effects of insulin glargine and liraglutide therapy on liver fat as measured by magnetic resonance in patients with type 2 diabetes: a randomized trial. Diabetes Care.

[26] Loomba R., Hartman M.L., Lawitz E.J. (2024). Tirzepatide for metabolic dysfunction-associated steatohepatitis with liver fibrosis. N Engl J Med.

[27] Thoma C., Day C.P., Trenell M.I. (2012). Lifestyle interventions for the treatment of non-alcoholic fatty liver disease in adults: a systematic review. J Hepatol.

[28] Khoo J., Hsiang J.C., Taneja R. (2019). Randomized trial comparing effects of weight loss by liraglutide with lifestyle modification in non-alcoholic fatty liver disease. Liver Int.

[29] Khoo J., Hsiang J., Taneja R. (2017). Comparative effects of liraglutide 3 mg vs structured lifestyle modification on body weight, liver fat and liver function in obese patients with non-alcoholic fatty liver disease: a pilot randomized trial. Diabetes Obes Metab.

[30] Vedtofte L., Bahne E., Foghsgaard S. (2020). One year's treatment with the glucagon-like peptide 1 receptor agonist liraglutide decreases hepatic fat content in women with nonalcoholic fatty liver disease and prior gestational diabetes mellitus in a randomized, placebo-controlled trial. J Clin Med.

[31] Shiomi M., Tanaka Y., Takada T. (2020). Determining whether the effect of liraglutide on non-alcoholic fatty liver disease depends on reductions in the body mass index. JGH Open.

[32] Engström A., Wintzell V., Melbye M. (2024). Association of glucagon-like peptide-1 receptor agonists with serious liver events among patients with type 2 diabetes: a Scandinavian cohort study. Hepatology.

[33] Krishnan A., Schneider C.V., Hadi Y. (2024). Cardiovascular and mortality outcomes with GLP-1 receptor agonists vs other glucose-lowering drugs in individuals with NAFLD and type 2 diabetes: a large population-based matched cohort study. Diabetologia.

[34] Ast J., Broichhagen J., Hodson D.J. (2021). Reagents and models for detecting endogenous GLP1R and GIPR. EBioMedicine.

[35] Matikainen N., Söderlund S., Björnson E. (2019). Liraglutide treatment improves postprandial lipid metabolism and cardiometabolic risk factors in humans with adequately controlled type 2 diabetes: a single-centre randomized controlled study. Diabetes Obes Metab.

[36] Kasher-Meron M., Youn D.Y., Zong H. (2019). Lipolysis defect in white adipose tissue and rapid weight regain. Am J Physiol Endocrinol Metab.

[37] van Baak M.A., Mariman E.C.M. (2023). Obesity-induced and weight-loss-induced physiological factors affecting weight regain. Nat Rev Endocrinol.

[38] Hjorth M.F., Christensen L., Kjølbæk L. (2020). Pretreatment Prevotella-to-Bacteroides ratio and markers of glucose metabolism as prognostic markers for dietary weight loss maintenance. Eur J Clin Nutr.

[39] Kassim S.Y., Gharib S.A., Mecham B.H. (2007). Individual matrix metalloproteinases control distinct transcriptional responses in airway epithelial cells infected with *Pseudomonas aeruginosa*. Infect Immun.

[40] García-Irigoyen O., Latasa M.U., Carotti S. (2015). Matrix metalloproteinase 10 contributes to hepatocarcinogenesis in a novel crosstalk with the stromal derived factor 1/C-X-C chemokine receptor 4 axis. Hepatology.

[41] Sze K.M., Chu G.K., Lee J.M. (2013). C-terminal truncated hepatitis B virus x protein is associated with metastasis and enhances invasiveness by C-Jun/matrix metalloproteinase protein 10 activation in hepatocellular carcinoma. Hepatology.

[42] Hu X., Yang L., Yu W. (2020). Association of serum fibroblast growth factor 23 levels with the presence and severity of hepatic steatosis is independent of sleep duration in patients with diabetes. Diabetes Metab Syndr Obes.

[43] Hu X., Ma X., Luo Y. (2018). Associations of serum fibroblast growth factor 23 levels with obesity and visceral fat accumulation. Clin Nutr.

[44] Hang H., Yuan S., Yang Q. (2014). Multiplex bead array assay of plasma cytokines in type 2 diabetes mellitus with diabetic retinopathy. Mol Vis.

[45] Mastropasqua R., D'Aloisio R., Di Nicola M. (2018). Relationship between aqueous humor cytokine level changes and retinal vascular changes after intravitreal aflibercept for diabetic macular edema. Sci Rep.

[46] Larsson A., Carlsson L., Lind A.L., Gordh T. (2015). The body mass index (BMI) is significantly correlated with levels of cytokines and chemokines in cerebrospinal fluid. Cytokine.

[47] Ponce-de-Leon M., Linseisen J., Peters A., Linkohr B. (2022). Novel associations between inflammation-related proteins and adiposity: a targeted proteomics approach across four population-based studies. Transl Res.

[48] Liu Q., Cai B.Y., Zhu L.X. (2020). Liraglutide modulates gut microbiome and attenuates nonalcoholic fatty liver in db/db mice. Life Sci.

[49] Ying X., Rongjiong Z., Kahaer M. (2023). Therapeutic efficacy of liraglutide versus metformin in modulating the gut microbiota for treating type 2 diabetes mellitus complicated with nonalcoholic fatty liver disease. Front Microbiol.

[50] Shang J., Liu F., Zhang B. (2021). Liraglutide-induced structural modulation of the gut microbiota in patients with type 2 diabetes mellitus. PeerJ.

[51] Crovesy L., Masterson D., Rosado E.L. (2020). Profile of the gut microbiota of adults with obesity: a systematic review. Eur J Clin Nutr.

[52] Sakamoto Y., Yoshio S., Doi H. (2021). Increased frequency of dysfunctional Siglec-7. Front Immunol.

[53] Dai W., Choubey M., Patel S. (2021). Adipocyte CAMK2 deficiency improves obesity-associated glucose intolerance. Mol Metab.

[54] (2024). EASL–EASD–EASO Clinical Practice Guidelines on the management of metabolic dysfunction-associated steatotic liver disease (MASLD). J Hepatol.

