# Supplementary material for: Randomised trial comparing weight loss through lifestyle and GLP-1 receptor agonist therapy in people with MASLD
Source: JHEP Rep. 2025 Feb 21;7(5):101363. doi: 10.1016/j.jhepr.2025.101363 (PMC12060445; doi:10.1016/j.jhepr.2025.101363)
Supplement: Multimedia component 2 [file mmc2.docx]

**JHEP Reports**

**CTAT methods**

Tables for a “Complete, Transparent, Accurate and Timely account” (CTAT) are now mandatory for all revised submissions. The aim is to enhance the reproducibility of methods.

- Only include the parts relevant to your study
- Refer to the CTAT in the main text as ‘Supplementary CTAT Table’
- Do not add subheadings
- Add as many rows as needed to include all information
- Only include one item per row

**If the CTAT form is not relevant to your study, please outline the reasons why:**

|  |
| --- |

- 1. **Antibodies**

| **Name** | **Citation** | **Supplier** | **Cat no.** | **Clone no.** |
| --- | --- | --- | --- | --- |
| n/a |  |  |  |  |

- 1. **Cell lines**

| **Name** | **Citation** | **Supplier** | **Cat no.** | **Passage no.** | **Authentication test method** |
| --- | --- | --- | --- | --- | --- |
| n/a |  |  |  |  |  |

- 1. **Organisms**

| **Name** | **Citation** | **Supplier** | **Strain** | **Sex** | **Age** | **Overall n number** |
| --- | --- | --- | --- | --- | --- | --- |
| n/a |  |  |  |  |  |  |

- 1. **Sequence based reagents**

| **Name** | **Sequence** | **Supplier** |
| --- | --- | --- |
| n/a |  |  |

- 1. **Biological samples**

| **Description** | **Source** | **Identifier** |
| --- | --- | --- |
| Serum, | Human samples as part of clinical study |  |
| Adipose tissue | Human samples as part of clinical study |  |
| Stool | Human samples as part of clinical study |  |
| Adipose microdialysate | Human samples as part of clinical study |  |

- 1. **Deposited data**

| **Name of repository** | **Identifier** | **Link** |
| --- | --- | --- |
| **Github** | PRJEB66353 | <https://github.com/toryn13/LILIpaper>. |

- 1. **Software**

| **Software name** | **Manufacturer** | **Version** |
| --- | --- | --- |
| R | n/a | 4.2.2 |
| DADA2 |  | 1.26.0 |
| LIMMA |  | 3.52.3 |

- 1. **Other (*e.g*. drugs, proteins, vectors etc.)**

| n/a |  |  |
| --- | --- | --- |
|  |  |  |

- 1. **Please provide the details of the corresponding methods author for the manuscript:**

| Dr Toryn Poolman, t.poolman@ucl.ac.uk |
| --- |

**2.0 Please confirm for randomised controlled trials all versions of the clinical protocol are included in the submission. These will be published online as supplementary information.**

| The clinical trial protocol will be added as supplementary material |
| --- |
