# Supplementary material for: Randomised trial comparing weight loss through lifestyle and GLP-1 receptor agonist therapy in people with MASLD
Source: JHEP Rep. 2025 Feb 21;7(5):101363. doi: 10.1016/j.jhepr.2025.101363 (PMC12060445; doi:10.1016/j.jhepr.2025.101363)
Supplement: Multimedia component 4 [file mmc4.pdf]

Randomised trial comparing weight loss through lifestyle and GLP-1 receptor agonist therapy in people with MASLD

Ahmad Moolla[1], Toryn Poolman[1,2], Nantia Othonos[1], Jiawen Dong[1], Kieran Smith[1], Thomas Cornfield[1], Sarah White[1], David W. Ray[1,3,4], Sofia Mouchti[5], Ferenc E. Mózes[6], Helena Thomaides-Brears[5], Stefan Neubauer[6], Jeremy F. Cobbold[7,8], Leanne Hodson[1], Jeremy W. Tomlinson[1,*]

JHEP Reports **2025**. vol. 7 | 1–12

Check for updates

Background & Aims: Glucagon-like peptide 1 receptor agonist (GLP-1RA) therapies deliver histological benefit in people with metabolic dysfunction-associated steatotic liver disease (MASLD). Multiple mechanisms may be important including weight loss, improved glycaemic control and putative direct tissue-specific actions. Following cessation of GLP1-RA therapy, weight regain is common. To dissect the mechanisms underpinning their benefits, we conducted a prospective, randomised, experimental medicine study in people with MASLD, comparing GLP-1RA treatment (liraglutide) to matched lifestyle-induced weight loss and assessed the impact of treatment withdrawal.

Methods: Twenty-nine participants with MASLD, without type 2 diabetes underwent metabolic phenotyping including measurement *de novo* lipogenesis (DNL), liver magnetic resonance imaging, body composition, adipose tissue RNA sequencing, circulating proteome, and stool microbiome analysis. Participants were randomised to lifestyle (∼500 kcal energy deficit) or GLP1-RA treatment for 12 weeks, after which investigations were repeated, and treatment stopped; investigations were also repeated 12 weeks after treatment withdrawal.

Results: Matched weight loss was achieved in both arms. Body composition changes, reductions in alanine aminotransferase, liver steatosis, and disease activity were similar following both treatments. GLP-1RA treatment, but not lifestyle, improved glucose handling, fasting lipids, and significantly deceased DNL. The subcutaneous adipose transcriptome, circulating proteome profile and stool microbiome were not different between groups after treatment. However, 12 weeks after GLP1-RA (but not lifestyle) withdrawal, circulating MMP-10, IL10RB, FGF-23, and Flt3L were elevated, alongside dysregulated adipose gene expression.

Conclusions: Although matched weight loss through lifestyle or GLP-1RA have comparable effects on hepatic steatosis, GLP-1RA treatment had additional metabolic benefits on glucose homeostasis, lipid profiles, and DNL. However, GLP-1RA withdrawal may adversely impact the circulating proteome, adipose tissue gene expression, and the stool microbiome, predisposing to weight regain.

Clinical Trials Registration: This study is registered at EudraCT (2016-002045-36).

© 2025 The Authors. Published by Elsevier B.V. on behalf of European Association for the Study of the Liver (EASL). This is an open access article under the CC BY license (http://creativecommons.org/licenses/by/4.0/).

Introduction

Current estimates suggest that non-alcoholic fatty liver disease (NAFLD), more recently termed metabolic dysfunction-associated steatotic liver disease (MASLD), affects up to 30% of unselected populations with a prevalence that has risen rapidly over the past 15 years.[1] MASLD is now the most common chronic liver disease, the second leading worldwide indication for liver transplantation (and the leading indication in some populations[2]), and is tightly associated with type 2 diabetes (T2D) and obesity.[3] Although the more advanced stages are associated with liver-specific morbidity and mortality, there is increased cardiovascular risk across all stages and severity of MASLD which is highest in people with advanced fibrosis.[4–6] Despite the significant disease burden, only a single agent has been granted FDA approval[7] and the current mainstays of treatment for MASLD remain weight reduction, achieved through dietary and lifestyle measures, alongside aggressive cardiovascular risk reduction.[8]

Current evidence suggests that agents with the ability to induce significant weight loss and offer cardiovascular risk protection have clinical utility in the treatment of people with MASLD. Glucagon-like peptide 1 (GLP-1) receptor agonists (GLP-1RA) have a range of metabolically beneficial actions, acting peripherally and centrally to reduce appetite and promote satiety resulting in significant weight loss.[9] In addition, they enhance glucose-dependent insulin secretion from the pancreatic islets, improving glycaemic control in people with T2D. They also have important anti-inflammatory actions.[10] The cardioprotective impact of GLP-1RA therapy is now established with several large cardiovascular outcome trials

* Corresponding author. Address: Oxford Centre for Diabetes, Endocrinology & Metabolism, University of Oxford, Churchill Hospital, Headington, Oxford, OX3 7LJ, UK. Tel.: +44 186 585 7359; fax: +44 186 585 7213.
E-mail address: jeremy.tomlinson@ocdem.ox.ac.uk (J.W. Tomlinson).
https://doi.org/10.1016/j.jhepr.2025.101363

demonstrating clear benefits and together strongly suggest a class effect of GLP-1RA therapy.[11,12] GLP-1RA therapy is safe and well tolerated in most people and has a good side-effect profile suggesting no major concerns with respect to long-term use.

Specifically in the context of MASLD, the GLP-1RA, liraglutide, decreases alanine aminotransferase (ALT) in a dose-dependent manner[13] as well as limiting hepatic *de novo* lipogenesis (DNL),[14] a critical driver of hepatic triacylglycerol (TAG) accumulation. The LEAN study was the first to demonstrate (using histological endpoints) the ability of liraglutide to resolve metabolic dysfunction-associated steatohepatitis (MASH) in people with biopsy-proven disease, with and without T2D.[15] More recently, semaglutide, a more potent GLP1-RA, has achieved resolution of MASH in up to 59% of people and this was associated with a 13% reduction in weight.[16] However, in people with MASLD-cirrhosis, it failed to improve fibrosis, but was safe and well tolerated and conveyed significant metabolic improvements (weight reduction, improved glucose control, and lipid profiles).[17] Although not yet in the peer reviewed literature, there is emerging evidence to suggest that semaglutide may have a beneficial impact on liver fibrosis in the phase III ESSENCE trial.[18]

Over a 12-week intervention period, liraglutide (doses up to 3 mg daily) induces weight loss of ~5%,[19] however, it remains unclear as to whether the benefits conferred by GLP-1RA therapy in people with MASLD (and without co-existent T2D) are solely a result of weight loss. Weight loss is associated with significant improvements in MASLD and other metabolic variables, but it is plausible that there are additional benefits (over and above the magnitude of weight loss) mediated by GLP-1RA therapy. We have therefore, undertaken a prospective, randomised, controlled study in people with MASLD (but without T2D to limit the potential confounding impact of alterations in glycaemic control) to determine if matched weight loss (achieved through lifestyle intervention) has comparable effects to GLP-1RA therapy in people with MASLD. Treatment with pharmacotherapy for weight loss is often administered for a limited and defined time, and following cessation, weight regain is frequent.[20] We have therefore included an exploration of mechanisms that may differ following treatment withdrawal that may have relevance for weight regain.

Patients and methods

We conducted a single-centre, open-label, prospective, randomised phase IIa clinical trial to evaluate the effects of the GLP-1RA, liraglutide, compared with lifestyle-induced weight loss in people with a diagnosis of progressed MASLD over a period of 24 weeks. The clinical study protocol received full ethical approval from the East of England – Cambridgeshire and Hertfordshire Research Ethics Committee (REC ref. 16/EE/0403), and the National Health Service (NHS) Health Research Authority. The trial was sponsored and monitored by the Clinical Trials and Research Governance research support unit at University of Oxford, was registered as a Clinical Trial of an Investigational Medicinal Product with the UK Medicines and Healthcare Products Regulatory Agency and was registered with the European Clinical Trials Database (EudraCT Number 2016-002045-36). All participants provided informed written consent before participation.

Clinical protocol

Individuals aged 18–75 years with a diagnosis of progressed MASLD, defined as steatosis on imaging or histology along with ALT >19 IU/L for females or >30 IU/L for males, or liver biopsy showing MASH or liver fibrosis, or vibration-controlled transient elastography (FibroScan, Echosens, Paris, France) liver stiffness measurement >8 kPa. Liver biopsy histological scoring and liver stiffness measurements are presented in Table S1. People with an established diagnosis of T2D were excluded to remove any confounding effects of concomitant antidiabetic medications or alterations in glycaemic control.

Participants underwent baseline investigations including anthropometric and blood pressure measurements, fasting blood tests, assessment of DNL using deuterated water (see below), and a subcutaneous abdominal adipose tissue biopsy. Aspiration of adipocytes (~1 g of tissue) was achieved using a needle and syringe and liposuction following administration of local anaesthetic. Samples were snap frozen in liquid nitrogen before being stored at -80 °C before RNA extraction and RNA sequencing.

Following the completion of the investigations in the fasting state, a prolonged (180 min) oral glucose tolerance test (OGTT) was performed. At the start of the OGTT, a microdialysis catheter (CMA Microdialysis, Solna, Sweden) was inserted under local anaesthetic (2 ml, 1% lidocaine) into the subcutaneous adipose tissue, 5–10 cm lateral to the umbilicus to allow sampling of adipose tissue interstitial fluid. Using a microdialysis pump (CMA 106), the microdialysate solution (physiological sterile saline solution) was introduced into the catheter (perfusion rate = 0.3 µl/min) and samples collected at 30-min intervals until the completion of the OGTT. Serum sampling at 30-min intervals was performed alongside the microdialysis. Because of issues with equipment availability, microdialysis was only performed in a subset of participants (n = 18). The oral glucose load comprised 75 g glucose in addition to 0.5 g ^{13}C-glucose (CK Gas Ltd., Newtown Unthank, UK) to allow measurement of glucose oxidation through $^{13}CO_2$ generation (see below).

Body composition analysis was performed using dual-energy X-ray absorptiometry (DXA) (GE Lunar iDXA, GE Healthcare, Chalfont Saint Giles, UK). All participants completed the abbreviated International Physical Activity Questionnaire (IPAQ). Data were summarised and are presented as ‘physical activity’ per week.

Magnetic resonance spectroscopy and LiverMultiScan

All magnetic resonance studies were performed at the University of Oxford Centre for Clinical Magnetic Resonance Research (OCMR) using a 3 Tesla system (TIM Trio, Siemens Healthineers, Erlangen, Germany). Detailed methods are included within the Supplementary data.

Participants were then randomised by members of the investigative team using a random number block randomisation tool to either a lifestyle intervention program provided by a commercial weight management organisation (Slimming World, Alfreton, UK) or to liraglutide treatment (starting dose 0.6 mg/day, titrated over a 3-week period to 1.8 mg/day) (Novo Nordisk, Copenhagen, Denmark) with the aim of achieving matched weight loss (estimated at 5%) across both interventional arms over a 12-week period. The lifestyle intervention

included an approximate 500 kcal/day deficit, decreased consumption of fat (to <30% of dietary calorie intake) alongside increased fibre intake, principally through increased consumption of vegetables and fruit. No specific advice with regards to physical activity or exercise was provided. Study interventions were commenced within 1-week of baseline assessments. Interim visits at weeks 1, 4, and 8 assessed compliance, blood test monitoring and adverse drug effects. Those who suffered side effects and were not able to tolerate a 1.8 mg dose of liraglutide had a dose reduction to 1.2 mg daily (n = 2). Liraglutide injections were given before the OGTT on the study days. All biological samples collected were processed and stored at -80 °C (where applicable) in line with all relevant ethically agreed and regulatory protocols.

After 12 weeks of intervention, all the investigations described above were repeated. Treatments were then withdrawn (GLP-1RA and lifestyle advice) and then after a further 12 weeks (24 weeks after treatment initiation) a further set of investigations was repeated (Fig. S1).

Stable isotope measurements

De novo *lipogenesis*

Hepatic DNL was assessed based on the incorporation of deuterium from ^2H_2O in plasma water (Finnigan GasBench-II, ThermoFisher Scientific, Altrincham, UK) into VLDL-TG palmitate using gas chromatography-mass spectroscopy with monitoring ions with mass-to-charge ratios (*m/z*) of 270 (M+0) and 271 (M+1).[21]

Glucose oxidation

To assess glucose oxidation through $^{13}CO_2$ generation, a modified OGTT incorporating ^{13}C-glucose was performed. Breath samples were collected at 30-min intervals throughout the duration of the test. $^{13}C/^{12}C$ ratios in breath samples and the relative rate of whole-body fatty acid oxidation was calculated as previously described.[22] The rate of expiration of $^{13}CO_2$ in breath was calculated by multiplying the estimated VCO_2 by the enrichment of breath CO_2.

Biochemical and targeted proteomic analysis

Fasting biochemical bloods (full blood count, renal function, liver chemistry, and lipid profile) were analysed through the NHS clinical laboratories at Oxford University Hospitals NHS Foundation Trust. Serum insulin was measured using a commercially available ELISA (Mercodia, Uppsala, Sweden) according to the manufacturer protocol and insulin resistance (homeostatic model assessment for insulin resistance [HOMA-IR]) was calculated using established criteria. Concentrations of non-esterified fatty acids (NEFAs), triglyceride, glucose, total cholesterol, and HDL cholesterol were measured using commercially available kits on an ILAB600/ILAB650 clinical analyser (Instrumentation Laboratory UK, Warrington, UK). Targeted proteomic analysis was performed on samples acquired in the fasting state using a commercially available platform (O-link, Uppsala, Sweden). Microdialysis samples were analysed for glucose and glycerol levels, using a mobile photometric enzyme-kinetic analyser (CMA ISCUS Flex, Solna, Sweden).

RNA extraction and RNA sequencing

Total RNA from adipose tissue biopsies was extracted using the Tri-Reagent system (Sigma-Aldrich, Dorset, UK). Detailed methods are presented in the Supplementary data.

Microbiome and analysis and 16S rRNA sequencing

Stool samples were collected and analysed at baseline, 12 weeks, and 24 weeks. DNA extraction and 16S sequencing was undertaken by Omega Bioservices (Norcross, GA, USA). Detailed methods are presented in the Supplementary data.

Statistical analysis

The primary outcome for the study was the change in hepatic steatosis (baseline to 12 weeks) using magnetic resonance spectroscopy (MRS) and the study was powered to detect a 20% relative change over a 12-week intervention in both treatment arms (power = 0.8, α = 0.05). Where appropriate, AUC was calculated using the trapezoid method. Statistical analyses were undertaken using R version 4.3 (R Foundation for Statistical Computing, Vienna, Austria). Paired *t* tests (or their non-parametric equivalents if data were not normally distributed) were used to compare outcomes before and after intervention. Unpaired *t* tests (or non-parametric equivalents) were used to compare treatment arms. Where multiple comparisons were made over time, repeated measures ANOVA analyses were used. The statistical significance level for all results was set as p <0.05. Circulating protein levels obtained from the targeted proteomic analysis were normalised and $\log_2$ applied. Intensity values were analysed using LIMMA, utilising the duplicateCorrelation function to account for the related nature of the samples.

Results

Thirty-three of 49 volunteers who successfully passed screening were enrolled into the study between January 2017 and April 2019. Thirty-one participants (two declined after screening) were randomised to either the lifestyle or liraglutide intervention. At baseline, no participants had impaired fasting glucose, but five of 29 had impaired glucose tolerance (Table 1). Twenty-nine participants (n = 14 lifestyle, n = 15 liraglutide) completed the 12-week intervention required for evaluation of the primary study outcome. In the liraglutide treatment arm 13/15 people were able to tolerate the highest dose (1.8 mg/day) and two required a dose reduction to 1.2 mg daily. Twenty-four participants (n = 12 in both arms) were followed up at 24 weeks to evaluate the legacy effects of interventions (Fig. 1). Both groups were well-matched at baseline, except for total cholesterol and HDL cholesterol which were lower in the liraglutide-treated group. Baseline demographic and anthropometric data are presented in Table 1.

Impact of 12 weeks of active intervention

Body weight, composition, physical activity, and blood pressure

Both interventions achieved significant weight loss; the magnitude of weight loss was similar in both arms (-4.7 ± 1.0 *vs.* -5.2 ± 0.7 kg, lifestyle *vs.* liraglutide, p = 0.63) (Table 1) (Fig. 2A and B). Total weight loss was principally attributable to a reduction in total fat mass; lean mass did not change

Table 1. Baseline parameters in 29 people with MASLD and the impact of a 12-week intervention with either lifestyle or GLP-1RA therapy with liraglutide.

	Lifestyle (n = 14)		GLP-1RA, liraglutide (n = 15)	
	**Baseline**	**12 weeks**	**Baseline**	**12 weeks**
Demographics				
Age (years)	48 ± 4		48 ± 4	
Sex (% male)	50.0		53.3	
Weight, body composition and blood pressure				
Weight (kg)	104.2 ± 5.8	99.5 ± 5.4[§]	106.9 ± 5.6	101.7 ± 5.4[§]
BMI (kg/m^2)	36.4 ± 1.5	34.8 ± 1.4[§]	35.7 ± 1.7	33.9 ± 1.6[§]
Body fat (%)	45.0 ± 2.1	43.3 ± 2.2[§]	43.3 ± 2.5	41.9 ± 2.5[§]
Total fat mass (kg)	45.2 ± 3.4	41.9 ± 3.4[§]	45.3 ± 4.2	41.9 ± 4.0[§]
Total lean mass (kg)	54.7 ± 3.3	54.1 ± 3.1	57.6 ± 3.0	56.2 ± 3.0[§]
Estimated visceral adipose mass (kg)	2.4 ± 0.3	2.1 ± 0.2[‡]	2.4 ± 0.3	2.0 ± 0.3[§]
Blood pressure (mmHg) systolic (S)/diastolic (D)	S: 136 ± 4<br>D: 80 ± 2	S: 123 ± 2[§]<br>D: 71 ± 2[§]	S: 133 ± 3<br>D: 83 ± 2	S: 126 ± 3[‡]<br>D: 79 ± 2[*]
Physical activity				
Physical activity (IPAQ), time sitting (h/day)	7.9 ± 1.0	7.6 ± 1.2	6.5 ± 1.1	6.8 ± 1.2
Physical activity (IPAQ), total weekly activity (h)	12.2 ± 4.0	9.2 ± 2.5	15.3 ± 5.1	14.2 ± 4.9
Fasting lipid profile				
Triglyceride (mmol/L)	1.5 ± 0.2	1.3 ± 0.2	1.6 ± 0.2	1.2 ± 0.1[‡]
Total cholesterol (mmol/L)	5.0 ± 0.3	4.7 ± 0.2	4.1 ± 0.2[*]	3.8 ± 0.2[†,‡]
HDL cholesterol (mmol/L)	1.3 ± 0.1	1.2 ± 0.1[§]	1.0 ± 0.05[†]	1.0 ± 0.05[*]
non-HDL cholesterol (mmol/L)	3.7 ± 0.2	3.5 ± 0.2	3.1 ± 0.2	2.8 ± 0.2[*,‡]
NEFA (μmol/L)	517 ± 49	459 ± 43	521 ± 51	441 ± 41
Insulin and glucose metabolism				
HbA1c (mmol/mol)	37 ± 1	36 ± 1	39 ± 1	35 ± 1[§]
Fasting glucose (mmol/l)	4.9 ± 0.1	4.9 ± 0.1	5.0 ± 0.2	4.6 ± 0.1
Number with impaired glucose tolerance (%)	2 (13)	0	3 (21)	0
Glucose AUC over 180 min OGTT (mmol/L.min)	7.6 ± 0.5	7.4 ± 0.4	7.3 ± 0.4	6.0 ± 0.2[†,§]
Fasting insulin (pmol/L)	112 ± 18	91 ± 19[‡]	136 ± 24	125 ± 15
Insulin AUC over 180 min OGTT (pmol/L.min)	898 ± 111	815 ± 92	809 ± 104	795 ± 64
HOMA-IR	4.2 ± 0.8	3.3 ± 0.7[‡]	5.0 ± 0.8	4.3 ± 0.5
Glucose oxidation (breath $^{13}CO_2$ AUC) over 180 min OGTT (mmol/kg lean mass)	2.4 × 10^{-4} ± 2.0 × 10^{-5}	2.3 × 10^{-4} ± 1.6 × $^{-5}$	2.2 × 10^{-4} ± 1.6 × 10^{-5}	2.1 × 10^{-4} ± 1.8 × 10^{-5}
Adipose tissue				
Subcutaneous microdialysis interstitial glucose AUC over 180 min OGTT (mmol/L.h)[¶]	11.7 ± 0.9	10.4 ± 1.3	12.0 ± 1.7	9.0 ± 1.4[‡]
Subcutaneous microdialysis interstitial glycerol AUC over 180 min OGTT (μmol/L.h)[¶]	547 ± 66	573 ± 69	566 ± 71	507 ± 35
ADIPO-IR (fasting NEFA × fasting insulin) (mmol/L.h × μmol/L.h)	60.3 ± 13.6	46.1 ± 12.2	77.1 ± 17.5	55.5 ± 8.8
Liver biochemistry				
ALT (IU/L)	61 ± 8	47 ± 8[§]	58 ± 8	46 ± 6[‡]
AST (IU/L)	37 ± 4	32 ± 5	36 ± 3	31 ± 4
Magnetic resonance spectroscopy and imaging				
MRS hepatic steatosis (%)	21.1 ± 2.4	13.6 ± 2.1[§]	24.3 ± 2.3	18.1 ± 1.9[§]
Intrahepatic iron (mg/g dry weight)	1.2 ± 0.1	1.1 ± 0.1[§]	1.2 ± 0.1	1.2 ± 0.1
Iron corrected T1 (cT1) (ms)	861 ± 23	813 ± 23[§]	872 ± 29	827 ± 24[§]
Non-invasive markers of liver fibrosis				
FIB-4	1.1 ± 0.2	1.1 ± 0.1	0.9 ± 0.1	0.9 ± 0.1
FIB-4 risk stratification; low/indeterminate/high (n)	10/4/0	10/4/0	12/3/0	13/2/0
NFS	-1.3 ± 0.3	-1.2 ± 0.3	-1.9 ± 0.4	-1.9 ± 0.3
NFS risk stratification; low/indeterminate/high (n)	6/8/0	7/7/0	10/5/0	9/6/0
Hepatic DNL				
Fasting DNL (%)	12.9 ± 1.9	10.7 ± 2.1	15.2 ± 2.5	9.7 ± 1.9[‡]
DNL AUC over 180 min OGTT (%.min)	13.0 ± 1.9	11.5 ± 1.9	14.4 ± 2.1	10.2 ± 1.8[‡]

Data presented are mean ± SEM unless otherwise stated.

ALT, alanine aminotransferase; AST, aspartate aminotransferase; AUC, area under curve; DNL, *de novo* lipogenesis; FIB-4, fibrosis-4; GLP-1RA, glucagon-like peptide 1 receptor agonist; HOMA-IR, homeostatic model assessment for insulin resistance; IPAQ, International Physical Activity Questionnaire; MASLD, metabolic dysfunction-associated steatotic liver disease; MRS, magnetic resonance spectroscopy; NEFA, non-esterified fatty acid; NFS, NAFLD fibrosis score; OGTT, oral glucose tolerance test.

[*]*p* <0.05.

[†]*p* <0.01 lifestyle *vs.* GLP-1RA.

[‡]*p* <0.05.

[§]*p* <0.01 baseline *vs.* 12 weeks within group, paired or unpaired *t* tests where appropriate.

[¶]Data from n = 10 (liraglutide) and n = 8 (lifestyle).

significantly in the lifestyle group but decreased in the GLP1-RA group (Table 1) (Fig. 2C and D). Percent fat mass and estimated visceral fat mass decreased in both groups, and there were no significant differences between the two arms (Table 1) (Fig. 2E and F). Systolic blood pressure improved in both groups, and diastolic blood pressure improved in the

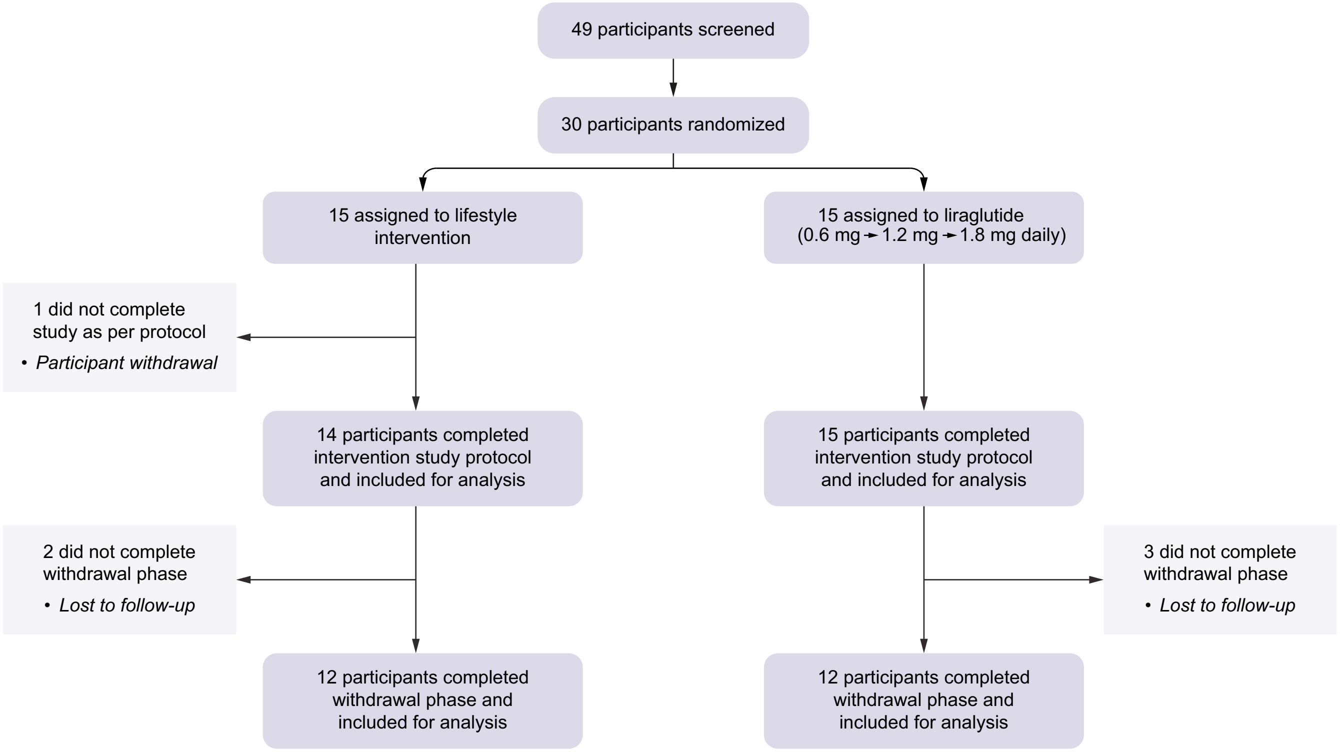

Fig. 1. Consort diagram summarising participant recruitment and analysis.

lifestyle group only, which remained lower than the GLP-1RA group at 12 weeks (Table 2). Physical activity as assessed by IPAQ data did not change in either arm (Table 1).

Glucose and lipid homeostasis

The study only recruited people without a diagnosis of T2D and all people had normal fasting glucose levels at baseline, and this did not change significantly in either arm after 12 weeks of intervention. Impaired glucose tolerance resolved in five of five participants irrespective of their treatment arm. Glycated haemoglobin decreased significantly in the GLP-1RA treatment arm, but not in those undergoing lifestyle intervention (Table 1). Consistent with these data, AUC glucose across the prolonged OGTT was reduced by GLP-1RA and not by lifestyle (Table 1). In addition, subcutaneous adipose tissue interstitial glucose across the OGTT as measured by microdialysis decreased significantly in the GLP-1RA group only (Table 1). Insulin sensitivity increased as assessed by fasting insulin levels, HOMA-IR improved only in the lifestyle group. Insulin concentrations across the prolonged OGTT did not change significantly in either group, nor did glucose oxidation (as measured by $^{13}CO_2$ appearance in breath) (Table 1). In the GLP-1RA treated group only, fasting triglycerides, total cholesterol, and non-HDL cholesterol all decreased. Lifestyle was associated with a small but significant decrease in HDL cholesterol (Table 1). Although fasting NEFA levels decreased in both treatment groups, this failed to reach statistical significance. There was also no significant change in adipose tissue insulin resistance as measured by adipo-IR. Adipose tissue interstitial glycerol levels across the OGTT did not change in either treatment arm (Table 1).

Liver biochemistry, hepatic steatosis, and DNL

ALT levels decreased in both treatment arms. There were no significant differences between the two interventions; there were no significant changes in aspartate aminotransferase (AST) (Table 1) (Fig. 3A and B). MRS demonstrated similar reductions in hepatic steatosis in both treatment groups (Table 1) (Fig. 3C). Liver iron content decreased in the lifestyle group only, and iron corrected T1 (cT1), a biomarker of hepatic fibroinflammation, decreased to a similar extent in both groups (Table 1) (Fig. 3D, Fig. S2). Although both fasting DNL and DNL measured across the prolonged OGTT decreased in both arms, this only reached statistical significance in the GLP-1RA-treated group (Table 1).

Circulating proinflammatory cytokine profiles

Ninety-two proinflammatory cytokines were measured in serum samples from all participants and there were no significant differences between the two groups at baseline. When adjusted for multiple comparisons, there were no significant changes in circulating proinflammatory cytokines levels after GLP-1RA treatment or lifestyle intervention. There were no significant differences between the two arms of the study at the end of the intervention period (Supplementary data).

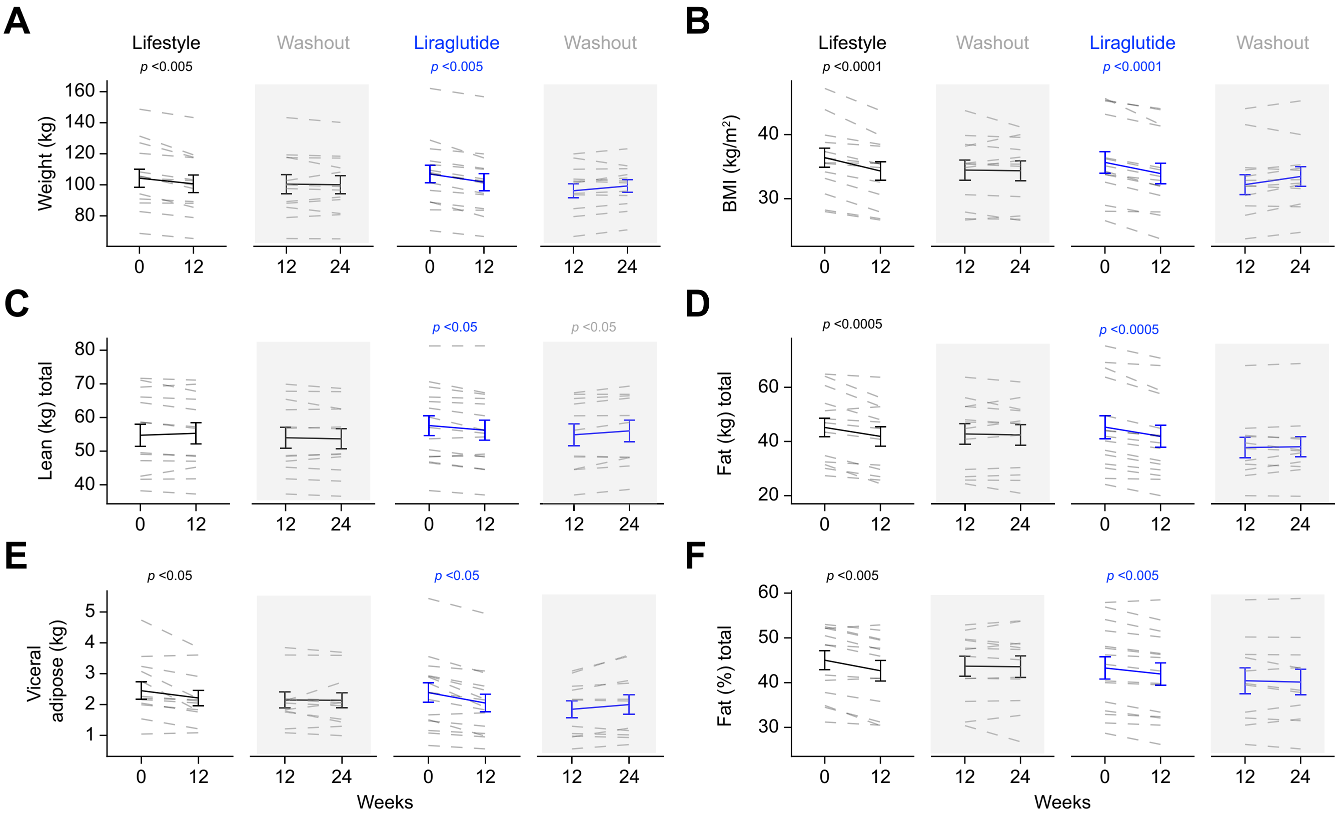

Fig. 2. Weight and body composition changes following lifestyle or GLP-1RA induced weight loss. Lifestyle intervention and treatment with GLP-1RA for 12 weeks are associated with significant, and comparable weight loss (A and B). Total and regional fat mass are decreased and there is a small reduction in lean mass in people treated with GLP1-RA (C–F). With the exception of a modest increase in lean mass following GLP-1RA withdrawal, body composition changes are maintained 12 weeks after interventional withdrawal (paired *t* test). GLP-1RA, glucagon-like peptide 1 receptor agonist.

RNA sequencing of abdominal subcutaneous adipose

RNA-sequencing analysis of subcutaneous adipose tissue revealed no significant differentially expressed genes (DEGs) at baseline between the two treatment groups. After 12 weeks of intervention, there remained no significant DEGs between groups. However, 14 DEGs were identified comparing samples before and after GLP-1RA treatment (Table 2). There were no DEGs before and after the lifestyle intervention.

Microbiome

At baseline, analysis of the 16S microbiome RNA sequencing demonstrated no significant differences in absolute or relative

Table 2. Differentially expressed genes in abdominal subcutaneous adipose tissue biopsies after lifestyle and GLP-1RA treatment.

Gene	*p* value	Adjusted *p* value	LogFC	Gene description
**Differentially expressed genes (baseline *vs.* 12 weeks of GLP-1RA treatment)**				
HTRA2	8.62×10^{-7}	0.017	2.08	HtrA serine peptidase 2
CPA6	3.95×10^{-6}	0.031	2.30	Carboxypeptidase A6
MDGA1	4.99×10^{-6}	0.031	2.01	MAM domain containing glycosylphosphatidylinositol anchor 1
CDC42EP1	6.48×10^{-6}	0.031	2.63	CDC42 effector protein 1
LINC00881	1.17×10^{-5}	0.036	1.80	Long intergenic non-protein coding RNA 881
ZFP62	1.46×10^{-5}	0.036	2.17	ZFP62 zinc finger protein
KRT73-AS1	1.54×10^{-5}	0.036	1.79	KRT73 antisense RNA 1
RALGPS2	1.61×10^{-5}	0.036	2.04	Ral GEF with PH domain and SH3 binding motif 2
SP8	1.70×10^{-5}	0.036	1.86	Sp8 transcription factor
APCS	2.34×10^{-5}	0.045	1.72	Amyloid P component, serum
SVBP	2.59×10^{-5}	0.045	2.09	Small vasohibin binding protein
PTCD2	3.07×10^{-5}	0.049	2.32	Pentatricopeptide repeat domain 2
SIGLEC7	3.31×10^{-5}	0.049	1.89	Sialic acid-binding Ig like lectin 7
SYCP1	3.65×10^{-5}	0.050	2.08	Synaptonemal complex protein 1
**Differentially expressed genes (GLP-1RA *vs.* lifestyle 12 weeks after treatment withdrawal)**				
GAB4	1.10×10^{-7}	0.0013	2.31	GRB2 associated binding protein family member 4
CAMK2B	1.33×10^{-7}	0.013	3.01	Calcium/calmodulin-dependent protein kinase II beta
VWDE	1.32×10^{-6}	0.0084	2.32	von Willebrand factor D and EGF domains
KLHL28	0.000005	0.026	2.20	Kelch like family member 28

abundances of microbiome phyla, notably Bacteroidetes and Firmicutes. In addition, the Firmicutes:Bacteroidetes ratio was similar in both groups. After 12 weeks of intervention with either lifestyle or liraglutide, there remained no significant differences between the groups (Fig. S3).

Mixed effects modelling

To determine the primary drivers of improvement in hepatic steatosis, mixed effects modelling was performed including age, sex, intervention, and change in weight. Weight reduction was the only variable included in the model that had a significant impact on the reduction in MRS measured liver fat content (p = 0.001).

Impact of treatment withdrawal

All treatments were withdrawn after 12 weeks. Twenty-four individuals (12 in each arm) were followed up at 24 weeks for assessment of legacy effects of either intervention. A separate analysis was then undertaken including individuals who completed all investigation visits (baseline, end of treatment, and 12 weeks post-treatment); participants with missing data were excluded from the analysis.

Following GLP1-RA withdrawal, weight increased by 1.3 ± 0.9 kg between the end of treatment and 12 weeks post-treatment, with a significant increase in lean mass (Table 3). After lifestyle withdrawal, there was a further modest reduction in weight (-0.4 ± 1.0 kg). However, when comparing weight change between arms from baseline to 12 weeks post-treatment and end of treatment to 12 weeks post-treatment, there were no significant differences. In addition, there were no significant changes in body composition when comparing the two treatment arms (Table 3) (Fig. 2).

The changes in ALT, hepatic steatosis, and cT1 all persisted through 12 weeks of treatment withdrawal and there were no significant differences between treatment groups during this washout phase. However, fasting DNL in both groups, and DNL across the prolonged OGTT in the lifestyle group alone, significantly increased following treatment withdrawal (Table 3).

The reductions in glucose AUC across the OGTT and HbA1c that were observed following 12 weeks of GLP-1RA treatment, reversed completely following treatment withdrawal (Table 3). In addition, fasting glucose increased significantly in the GLP-1RA treated group while decreased in the lifestyle group. Impaired glucose tolerance recurred in one participant following GLP-1RA withdrawal. At 12 weeks post-treatment, there were no significant differences in glucose parameters between treatment arms. Similarly circulating NEFA did not change in either group and although adipo-IR increased in both groups, it did not reach statistical significance.

Circulating proinflammatory cytokines did not change after lifestyle withdrawal, however, after adjusting for multiple comparisons, four cytokines (matrix metalloproteinase-10 [MMP-10], interleukin-10 receptor B [IL-10RB], fibroblast growth factor 23 [FGF23], and FMS-like tyrosine kinase 3 ligand [Flt3L]) increased significantly after liraglutide withdrawal (Fig. 4) (Supplementary data).

Although there were no adipose tissue DEGs at baseline or after 12 weeks of intervention between groups, after treatment withdrawal, four DEGs were identified comparing the liraglutide and lifestyle-treated participants (Table 2).

Pooled analysis of the stool microbiome did not demonstrate robust changes. Although there was a trend to an increased abundance of Firmicutes and deceased Bacteroidetes after GLP-1RA withdrawal (with opposing effects in the lifestyle group), these did not achieve statistical significance. There was no difference in the Firmicutes:Bacteroidetes ratio between groups (Fig. S3).

Discussion

We have demonstrated that over a 12-week intervention period, both lifestyle and GLP-1RA therapy with liraglutide achieved a comparable magnitude of weight loss, and this was associated with similar reductions in hepatic steatosis and fat mass. GLP-1RA had an additional impact to improve glucose homeostasis (in people without T2D) which was lost after treatment withdrawal. Significant reductions in DNL and improvements in fasting lipid profiles were only seen in the GLP-1RA-treated group. The beneficial impact of both interventions on the liver was maintained following the 12-week treatment washout period, although in people treated with GLP-1RA, treatment withdrawal was associated with changes in a small number of proinflammatory cytokines and transcriptional changes in adipose tissue and these may have important implications for potential weight regain.

The magnitude of weight loss with GLP-1RA therapy in this study was similar to that reported in the literature.[19] Furthermore, the observed reduction in liver fat content (6.2%) in the GLP-1RA arm is similar to previous studies in people with MASLD,[23,24] although this is not a consistent finding in all studies.[25] Improvements in cT1 were significant, although lower than observed in a recent GLP-1RA/GIP trial in people with MASH, over a 52-week treatment period.[26] The data with regards to lifestyle interventions are also comparable to the published literature. A meta-analysis of lifestyle interventions in people with MASLD identified weight loss of between 4% and14% with a corresponding reduction in liver fat of 4–10%.[27] We observed a 7.5% reduction in liver fat content in the lifestyle arm.

To date, very few studies have tried to directly compare the impact of GLP-1RA therapy with matched weight loss. Two published studies have used higher doses of liraglutide (3 mg) with a 26-week intervention period. Immediately following treatment, and similar to our observations, the reductions in weight and liver fat content were similar, although of note, there was increased weight regain in the liraglutide-treated group compared with the lifestyle intervention arm.[28,29]

There is now an established body of evidence demonstrating the beneficial impact of GLP-1RA in people with MASLD to decrease hepatic steatosis and resolve MASH[15,16,30,31] and also to improve cardiovascular and also liver outcomes.[32,33] However, the impact on liver fibrosis is less clear.[16,17] The improvements in lipid profiles that we observed specifically in the GLP-1RA-treated people may offer some explanation as to the mechanisms underpinning reductions in cardiovascular risk that are likely to occur in people with MASLD treated with these drugs (accepting that a dedicated cardiovascular outcome studies in people with MASLD, treated with GLP-1RA have not been performed).

Table 3. The impact of treatment withdrawal (lifestyle or GLP-1A) following 12 weeks of intervention in people with MASLD.

	Lifestyle (n = 12)		GLP-1RA, liraglutide (n = 12)	
	End of treatment	12-weeks post-intervention withdrawal	End of treatment	12-weeks post-intervention withdrawal
Weight, body composition and blood pressure				
Weight (kg)	100.4 ± 6.1	100.0 ± 5.9	97.9 ± 4.4	99.3 ± 4.1
BMI (kg/m^2)	34.5 ± 1.6	34.4 ± 1.5	33.0 ± 1.6	33.4 ± 1.5
Body fat (%)	43.7 ± 2.2	43.6 ± 2.4	40.4 ± 2.9	40.1 ± 2.9
Total fat mass (kg)	42.8 ± 3.8	42.4 ± 3.8	37.8 ± 3.7	38.0 ± 3.7
Total lean mass (kg)	54.0 ± 3.1	53.7 ± 3.0	54.9 ± 3.2	56.0 ± 3.2[§]
Estimated visceral adipose mass (kg)	2.1 ± 0.3	2.1 ± 0.3	1.8 ± 0.3	2.0 ± 0.3
Blood pressure (mmHg) systolic (S)/diastolic (D)	S: 121 ± 3<br>D: 71 ± 3	S: 128 ± 2[§]<br>D: 77 ± 3[‡]	S: 127 ± 4<br>D: 79 ± 2	S: 133 ± 4<br>D: 81 ± 2
Physical activity				
Physical activity (IPAQ), time sitting (h/day)	9.3 ± 1.6	8.3 ± 1.8	5.1 ± 0.6 a	4.7 ± 0.7
Physical activity (IPAQ), total weekly activity (h)	9.6 ± 3.0	6.5 ± 1.5	17.6 ± 5.8	19.5 ± 7.0
Fasting lipid profile				
Triglyceride (mmol/L)	1.2 ± 0.2	1.5 ± 0.4	1.2 ± 0.1	1.4 ± 0.1
Total cholesterol (mmol/L)	4.6 ± 0.2	5.0 ± 0.3[§]	3.9 ± 0.2	4.2 ± 0.2[§]
HDL cholesterol (mmol/L)	1.2 ± 0.1	1.3 ± 0.1[‡]	1.0 ± 0.1	1.1 ± 0.1
non-HDL cholesterol (mmol/L)	3.4 ± 0.2	3.7 ± 0.3[‡]	2.9 ± 0.2	3.1 ± 0.2[‡]
NEFA (µmol/L)	437 ± 34	389 ± 47	459 ± 47	407 ± 55
Insulin and glucose metabolism				
HbA1c (mmol/mol)	36 ± 1	37 ± 1	35 ± 1	37 ± 1.0[‡]
Fasting glucose (mmol/L)	4.9 ± 0.1	4.7 ± 0.2[‡]	4.5 ± 0.1[*]	5.1 ± 0.2[‡]
Number with impaired glucose tolerance (%)	0	0	1 (8)	0
Glucose AUC over 180 min OGTT (mmol/L.min)	7.5 ± 0.4	7.1 ± 0.4	6.1 ± 0.3[†]	7.1 ± 0.5
Fasting insulin (pmol/L)	92 ± 22	110 ± 31	127 ± 17	127 ± 27
Insulin AUC over 180min OGTT (pmol/L.min)	727 ± 110	685 ± 154	833 ± 75	682 ± 129
HOMA-IR	3.3 ± 0.8	3.7 ± 1.1	4.2 ± 0.6	4.4 ± 1.1
Glucose oxidation (Breath$^{13}CO_2$ AUC) over 180 min OGTT (mmol/kg lean mass)	$2.2 \times 10^{-4} \pm 1.5 \times 10^{-5}$	$2.7 \times 10^{-4} \pm 1.8 \times 10^{-5}$[§]	$2.1 \times 10^{-4} \pm 2.0 \times 10^{-5}$	$2.4 \times 10^{-4} \pm 2.0 \times 10^{-5}$
Liver biochemistry				
ALT (IU/L)	48 ± 9	50 ± 12	46 ± 7	54 ± 10
AST (IU/L)	33 ± 5	38 ± 11	31 ± 4	36 ± 4
Adipose tissue				
ADIPO-IR (fasting NEFA × fasting insulin) (mmol/L × pmol/L)	43.8 ± 14.8	54.8 ± 17.2	57.9 ± 10.2	64.2 ± 14.0
Magnetic resonance spectroscopy and imaging				
MRS hepatic steatosis (%)	14.0 ± 2.5	15.3 ± 2.9	18.5 ± 2.4	20.8 ± 3.1
Intrahepatic iron (mg/g dry weight)	1.1 ± 0.1	1.1 ± 0.1	1.3 ± 0.2	1.2 ± 0.1
Iron corrected T1 (cT1) (ms)	818 ± 30	821 ± 32	819 ± 30	845 ± 29
Non-invasive markers of liver fibrosis				
FIB-4	1.1 ± 0.2	1.2 ± 0.2	0.9 ± 0.1	1.0 ± 0.1[§]
FIB-4 risk stratification; low/indeterminate/high (n)	8/4/0	8/4/0	10/2/0	10/2/0
NFS	-1.0 ± 0.3	-1.0 ± 0.4	-2.0 ± 0.4	-2.0 ± 0.4
NFS risk stratification; low/indeterminate/high (n)	5/7/0	4/8/0	8/4/0	8/4/0
Hepatic DNL				
Fasting DNL (%)	9.4 ± 2.1	14.1 ± 2.6[‡]	9.2 ± 2.7	14.6 ± 2.3[‡]
DNL AUC over 180 min OGTT (%.min)	10.1 ± 2.3	16.2 ± 3.5[‡]	9.3 ± 2.4	13.3 ± 2.2

Data are presented as mean ± SEM unless otherwise stated.

ALT, alanine aminotransferase; AST, aspartate aminotransferase; AUC, area under curve; DNL, *de novo* lipogenesis; FIB-4, fibrosis-4; GLP-1RA, glucagon-like peptide 1 receptor agonist; HOMA-IR, homeostatic model assessment for insulin resistance; IPAQ, International Physical Activity Questionnaire; MRS, magnetic resonance spectroscopy; NEFA, non-esterified fatty acid; NFS, NAFLD fibrosis score; OGTT, oral glucose tolerance test.

[*] $p < 0.05$.

[†] $p < 0.01$ lifestyle *vs.* GLP-1RA.

[‡] $p < 0.05$.

[§] $p < 0.01$ end of treatment *vs.* 12-weeks post-treatment within group; paired or unpaired *t* tests where appropriate.

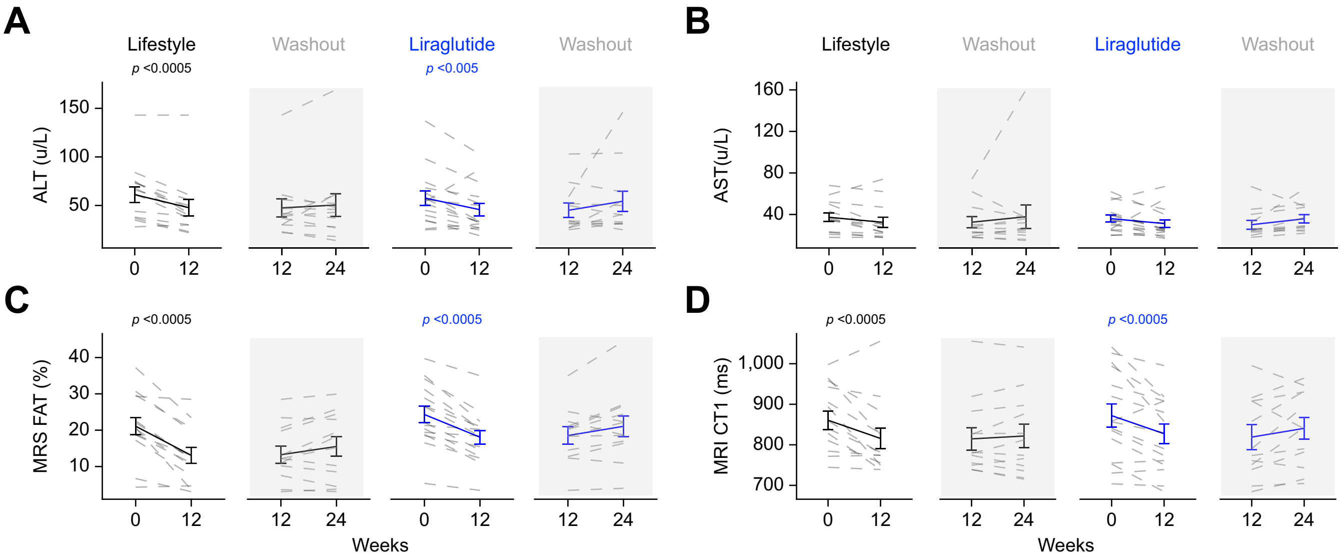

Fig. 3. Changes in liver biochemistry, imaging and *de novo* lipogenesis following lifestyle or GLP-1RA induced weight loss. Lifestyle intervention and treatment with GLP-1RA for 12-weeks are associated with similar reductions in ALT, but not AST (A and B). Both interventions significantly decreased liver fat content as well as cT1 values on MR scanning (C and D) (paired *t* test). ALT, alanine aminotransferase; AST, aspartate aminotransferase; cT1, iron corrected T1; GLP-1RA, glucagon-like peptide 1 receptor agonist; MRS, magnetic resonance spectroscopy.

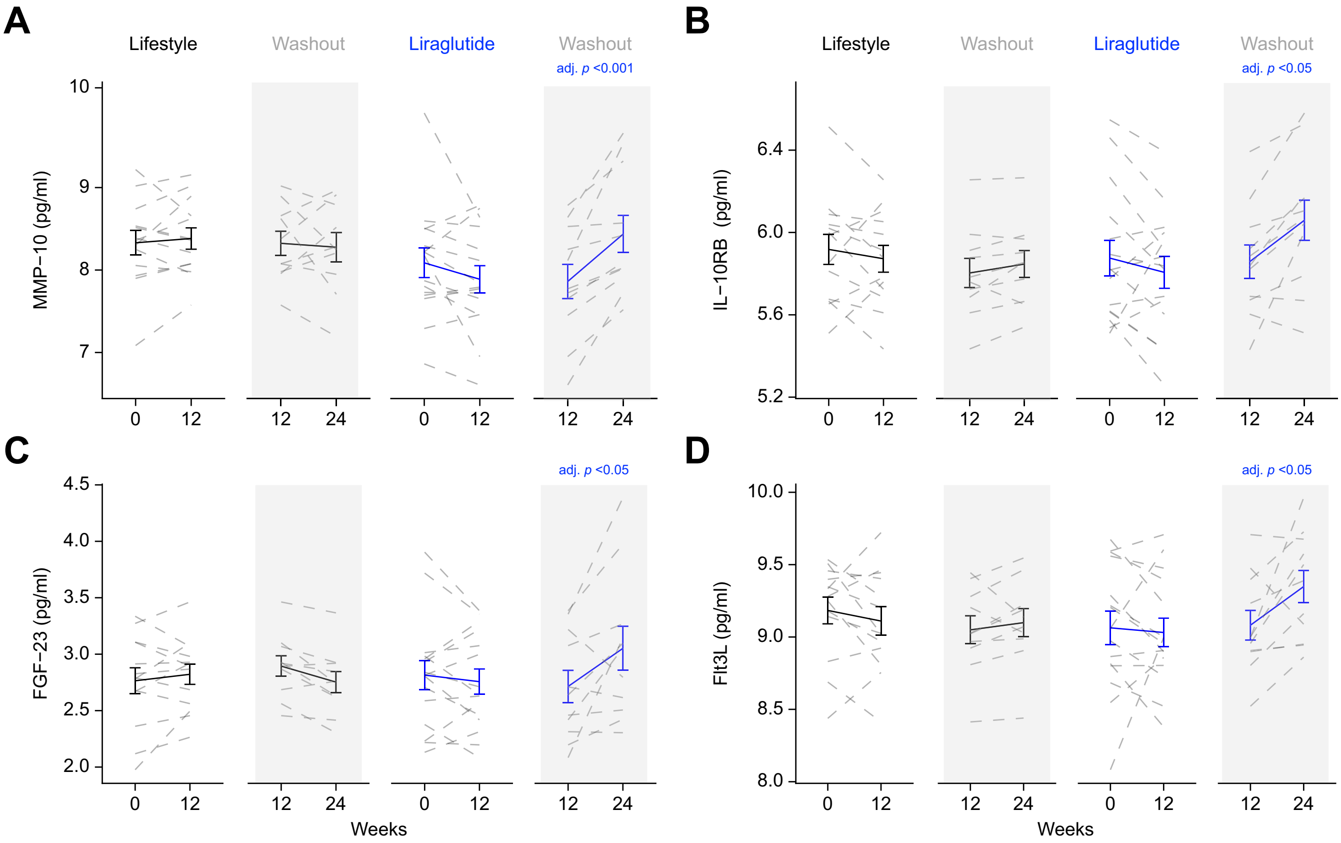

Fig. 4. Circulating cytokine profiles following lifestyle- or GLP-1RA-induced weight loss. Lifestyle intervention and treatment with GLP-1RA for 12 weeks are not associated with changes in circulating proinflammatory cytokines. However, 12 weeks after treatment withdrawal, circulating levels of matrix metalloproteinase-10 (MMP-10) (A), interleukin-10 receptor B (IL-10RB) (B), fibroblast growth factor-23 (FGF-23) (C) and FMS-like tyrosine kinase 3 ligand (Flt3L) (D), increased significantly, only in those people who had been treated with GLP-1RA (paired *t* test). GLP-1RA, glucagon-like peptide 1 receptor agonist.

A putative direct impact of GLP-1RA therapy on the liver remains controversial. GLP-1 has a well-established role to regulate glucose-sensitive insulin secretion from the pancreatic islet, but GLP-1 receptors are also expressed in extra-pancreatic tissues. Although some studies have suggested that the GLP-1 receptor (at the mRNA level) may be expressed in human liver, more recent studies using a variety of robust methodologies have concluded that there is no expression in human hepatocytes.[34] This lack of functional receptor activity may explain some of the variability of the impact of GLP-1RA to limit hepatic DNL which has been reported in some, but not all studies.[14,35] In the current study, DNL decreased in both arms, but only reached statistical significance in the GLP-1RA-treated people. It is possible that this additional benefit with GLP-1RA, over and above weight loss could reflect improved glucose handling, decreasing substrate availability (glucose) for DNL. Glucose handling worsened following GLP-1RA withdrawal, and this might explain the rebound and the observed increase in DNL, however, in the lifestyle group, after treatment withdrawal, DNL also increased despite no change in glucose homeostasis. It remains possible that the mechanisms underpinning these changes differ between treatment arms, but that in both situations, treatment withdrawal may lead to increased DNL that may fuel re-accumulation of hepatic TAG.

Weight regain remains an important and challenging clinical problem. It is often overlooked in clinical studies and occurs after all weight loss interventions. In the same way that mechanisms of weight loss differ across all interventions (as does their relative efficacy), the trajectory of weight regain may also differ. The mechanisms that contribute to weight regain are multiple and complex. There are changes in lipolytic function in adipose tissue[36] alongside changes in lipid oxidation, alterations in circulating regulators of appetite[37] as well as emerging data suggesting that changes in the microbiome may play a role in the predisposition to weight regain.[38] In this study, we observed differences in circulating proinflammatory cytokines, the adipose tissue transcriptome, and the gut microbiome that only became apparent after treatment withdrawal.

The clinical significance of the changes that we observed in the circulating cytokine profiles after GLP-1RA withdrawal remain to be determined. MMP-10 (stomelysin-2) is highly expressed in epithelial cells. Although mice with genetic deletion to not display a clear metabolic phenotype,[39] MMP-10 has been implicated in the development of hepatocellular carcinoma.[40,41] FGF-23 is principally regarded as a bone-derived regulator of phosphate and vitamin D homeostasis. Positive associations between circulating FGF-23 levels and hepatic steatosis, adiposity, and circulating hyperlipidaemia have been described[42,43] and therefore following weight loss intervention withdrawal, a differential impact on circulating FGF-23 levels could have a metabolic impact. Flt3L has complex immune regulatory functions and its role in metabolic disease has not been explored. A single study has reported lower circulating levels in people with diabetic retinopathy (compared with healthy control participants),[44] with evidence for decreasing aqueous humour levels after treatment for diabetic macular oedema using anti-VEGF therapy.[45] Similarly, the role of IL-10RB in regulating metabolic phenotype is largely unknown. However, both circulating and cerebrospinal fluid levels are positively associated with BMI[46,47] although whether this is a cause or consequence cannot be determined.

Our analysis of the microbiome was compromised by limited sample numbers necessitating a pooled analysis. However, following intervention withdrawal, we did observe a trend towards increased Firmicutes and decreased Bacteroidetes, specifically following GLP-1RA (but not lifestyle) treatment. There is evidence from rodent studies to suggest that GLP-1RA treatment can impact the microbiome,[48] but clinical studies are sparse and although some have suggested that the impact of liraglutide may be modest (in comparison with metformin),[49] others have demonstrated more robust effects.[50] However, in the studies published to date, the specific impact of weight loss, as distinct from effects on blood glucose control, cannot be disentangled. Of interest, many studies have reported that an increased Firmicutes:Bacteroidetes ratio (as we observed following GLP-1RA withdrawal) is associated with obesity, and this may have implications for future weight regain in this group.[51]

Lifestyle intervention had no impact on subcutaneous abdominal adipose tissue gene expression profiles. However, GLP-1RA treatment was associated with a small number of gene expression changes (Table 2). Most of the identified DEGs have not been examined in the context of MASLD, however, in people with MASLD, altered natural killer cell expression of Siglec-7 (Sialic acid-binding immunoglobulin-like lectin-7), that is associated with compromised NK cell function has been described.[52] Four DEGs between treatment arms were identified following treatment withdrawal at 24 weeks. Calcium/calmodulin-dependent protein kinase II beta (CAMK2B) was significantly increased (>8-fold) in the liraglutide treatment group. Although the precise impact of this is not clear, it is interesting to note that genetic inhibition of CAMK2 signalling specifically in adipose tissue (through conditional genetic deletion of other CAM2K subunits, delta and gamma) improves adipose insulin sensitivity and glucose tolerance[53] and this may contribute to the rebound effects that we observed after GLP-1RA withdrawal.

The current study does have some limitations. Although this was a relatively small study over a short duration, it incorporated a dedicated washout period (which is often omitted in clinical experimental studies) alongside deep metabolic phenotyping to provide a multisystem assessment of the impact of intervention and treatment withdrawal. There was no placebo-treated group to act as comparator to both GLP-RA and lifestyle intervention. Although established liver and cardiometabolic parameters were assessed, the outcome measures did not include a liver biopsy which continues to be the gold standard assessment of MASLD stage and severity (including MASH and fibrosis). Over this duration, it is unlikely that significant changes in liver fibrosis would have been observed and therefore non-invasive assessments to measure fibrosis specifically, were not included. The study only included people without T2D, but this was felt to be a crucial element of the study design in attempting to control for variables (other than weight) that might influence MASLD progression. In addition, although the formal lifestyle intervention was withdrawn, it is entirely plausible that elements of the lifestyle programme were continued by the participants during the washout phase (contrasting with liraglutide withdrawal), and this could explain differences in the propensity to weight regain and changes in metabolic variables measured at the end of the washout phase.

In conclusion, we have demonstrated that weight loss is the major contributor to the improvement in hepatic steatosis. We did observe differences in glucose handling, lipid profiles and DNL that might favour GLP-1RA treatment and perhaps explain improvements in cardiovascular risk, but this could be off-set by the impact of treatment withdrawal. This also brings into focus the clinical impact of intermittent use of GLP-1RA treatment, but the long-term consequences of this need to be explored in larger scale studies of longer duration.

The data from this study advocate that weight loss should remain the primary focus to improve outcomes for people with MASLD and that pharmacotherapy, including the use of GLP1-RAs may be important tools in trying to achieve this, as recommended in recent international practice guidance.[54] With either intervention, there were significant benefits both metabolically and specifically to the liver. Importantly, our observations suggest that in contrast to ongoing intervention, treatment withdrawal may have a differential impact on individuals with MASLD. The precise consequences of this need to be explored in more detail, but there could be clinical implications for weight maintenance or regain.

Affiliations

[1]*Oxford Centre for Diabetes Endocrinology & Metabolism (OCDEM), NIHR Oxford Biomedical Research Centre, University of Oxford, Churchill Hospital, Oxford, UK;* [2]*Structural and Molecular Biology, Faculty of Life Sciences, University College London, London, UK;* [3]*NIHR Oxford Health Biomedical Research Centre, University of Oxford, Oxford, UK;* [4]*Oxford Kavli Centre for Nanoscience Discovery, University of Oxford, Oxford, UK;* [5]*Perspectum, Gemini One, Oxford, UK;* [6]*Oxford Centre for Clinical Magnetic Resonance Research (OCMR), John Radcliffe Hospital, University of Oxford, Oxford, UK;* [7]*Department of Gastroenterology and Hepatology, Oxford University Hospitals, Oxford, UK;* [8]*NIHR Oxford Biomedical Research Centre, Oxford, UK*

Abbreviations

ALT, alanine aminotransferase; AST, aspartate aminotransferase; cT1, iron corrected T1; DEGs, differentially expressed genes; DNL, *de novo* lipogenesis; DXA, dual-energy X-ray absorptiometry; GLP-1, glucagon-like peptide 1; GLP-1RA, glucagon-like peptide 1 receptor agonist; HOMA-IR, homeostatic model assessment for insulin resistance; IPAQ, International Physical Activity Questionnaire; *m/z*, mass-to-charge ratio; MASH, metabolic dysfunction-associated steatohepatitis; MASLD, metabolic dysfunction-associated steatotic liver disease; MRS, magnetic resonance spectroscopy; NAFLD, non-alcoholic fatty liver disease; NEFA, non-esterified fatty acid; NHS, National Health Service; OGTT, oral glucose tolerance test; T2D, type 2 diabetes; TAG, triacylglycerol.

Financial support

Medical Research Council (MR/P011462/1 to JWT; MR/W019000/1 and MR/V034049/1 to DRW); National Institute for Health and Care Research (NIHR) Oxford Biomedical Research Centre (to JWT and DWR) and NIHR Oxford Health Biomedical Research Centre (NIHR203316); Novo Nordisk Fellowship, University of Oxford (to AM); British Heart Foundation (FS/15/56/31645 and FS/SBSRF/21/31013 senior fellowship to LH). FEM was funded by a Sir Henry Dale Fellowship awarded jointly by the Wellcome Trust and the Royal Society (221805/Z/20/Z). The views expressed are those of the authors and not necessarily those of the National Health Service, the NIHR, or the Department of Health.

Conflicts of interest

JWT has been an advisory board member for Novo Nordisk and a member of a data and safety monitoring committee for Novartis; JFC has been an advisory board member for Novo Nordisk and Intercept; consultancy for Alnylam; FEM, LH, TC, JD, and KS have nothing to declare. SM and HTB are employees at Perspectum, the company that developed LiverMultiScan. HTB is also a shareholder at Perspectum. SN is a founder and shareholder of Perspectum.

Please refer to the accompanying ICMJE disclosure forms for further details.

Authors' contributions

Conceptualisation: SN, JFC, JWT. Methodology: AM, TP, SM, FEM, HT-B, LH. Formal analysis: AM, TP, TC, SM, FEM, HT-B, JD, KS. Investigation: AM, NO, SW. Resources: LH, DWR, JFC, SN, JWT. Data curation: AM, TP, HT-B, LH, JWT. Writing – original draft: AM, JWT. Writing – review and editing, all authors. Supervision: SN, LH, JWT. Project administration: AA, LH, JWT. Funding acquisition, AA, LH, DWR, SN, JWT.

Data availability statement

Data, materials, and methods from this study are included in the Supplementary material. The sequencing data and associated analysis have been deposited under project ID PRJEB66353 and are available on GitHub at https://github.com/toryn13/LILIpaper. Additional details can be made available from the corresponding author upon request.

Supplementary data

Supplementary data to this article can be found online at https://doi.org/10.1016/j.jhepr.2025.101363.

References

[1] Younossi ZM, Golabi P, Paik JM, et al. The global epidemiology of nonalcoholic fatty liver disease (NAFLD) and nonalcoholic steatohepatitis (NASH): a systematic review. Hepatology 2023;77:1335–1347.

[2] Stepanova M, Kabbara K, Mohess D, et al. Nonalcoholic steatohepatitis is the most common indication for liver transplantation among the elderly: data from the United States Scientific Registry of Transplant Recipients. Hepatol Commun 2022;6:1506–1515.

[3] Paik JM, Golabi P, Younossi Y, et al. Changes in the global burden of chronic liver diseases from 2012 to 2017: the growing impact of NAFLD. Hepatology 2020;72:1605–1616.

[4] Dulai PS, Singh S, Patel J, et al. Increased risk of mortality by fibrosis stage in nonalcoholic fatty liver disease: systematic review and meta-analysis. Hepatology 2017;65:1557–1565.

[5] de Avila L, Henry L, Paik JM, et al. Nonalcoholic fatty liver disease is independently associated with higher all-cause and cause-specific mortality. Clin Gastroenterol Hepatol 2023;21:2588–2596.

[6] Taylor RS, Taylor RJ, Bayliss S, et al. Association between fibrosis stage and outcomes of patients with nonalcoholic fatty liver disease: a systematic review and meta-analysis. Gastroenterology 2020;158:1611. 25.e1612.

[7] Harrison SA, Bedossa P, Guy CD, et al. A phase 3, randomized, controlled trial of resmetirom in NASH with liver fibrosis. N Engl J Med 2024;390:497–509.

[8] Marjot T, Moolla A, Cobbold JF, et al. Nonalcoholic fatty liver disease in adults: current concepts in etiology, outcomes, and management. Endocr Rev 2020;41:bnz009.

[9] Ard J, Fitch A, Fruh S, et al. Weight loss and maintenance related to the mechanism of action of glucagon-like peptide 1 receptor agonists. Adv Ther 2021;38:2821–2839.

[10] Bray JJH, Foster-Davies H, Salem A, et al. Glucagon-like peptide-1 receptor agonists improve biomarkers of inflammation and oxidative stress: a systematic review and meta-analysis of randomised controlled trials. Diabetes Obes Metab 2021;23:1806–1822.

[11] Bethel MA, Patel RA, Merrill P, et al. Cardiovascular outcomes with glucagon-like peptide-1 receptor agonists in patients with type 2 diabetes: a meta-analysis. Lancet Diabetes Endocrinol 2018;6:105–113.

[12] Yoshiji S, Minamino H, Tanaka D, et al. Effects of glucagon-like peptide-1 receptor agonists on cardiovascular and renal outcomes: a meta-analysis and meta-regression analysis. Diabetes Obes Metab 2022;24:1029–1037.

[13] Armstrong MJ, Houlihan DD, Rowe IA, et al. Safety and efficacy of liraglutide in patients with type 2 diabetes and elevated liver enzymes: individual patient data meta-analysis of the LEAD program. Aliment Pharmacol Ther 2013;37:234–242.

[14] Armstrong MJ, Hull D, Guo K, et al. Glucagon-like peptide 1 decreases lipotoxicity in non-alcoholic steatohepatitis. J Hepatol 2016;64:399–408.

[15] Armstrong MJ, Gaunt P, Aithal GP, et al. Liraglutide safety and efficacy in patients with non-alcoholic steatohepatitis (LEAN): a multicentre, double-blind, randomised, placebo-controlled phase 2 study. Lancet 2016;387:679–690.
[16] Newsome PN, Buchholtz K, Cusi K, et al. A placebo-controlled trial of subcutaneous semaglutide in nonalcoholic steatohepatitis. N Engl J Med 2021;384:1113–1124.
[17] Loomba R, Abdelmalek MF, Armstrong MJ, et al. Semaglutide 2·4 mg once weekly in patients with non-alcoholic steatohepatitis-related cirrhosis: a randomised, placebo-controlled phase 2 trial. Lancet Gastroenterol Hepatol 2023;8:511–522.
[18] Plainsboro NJ. ESSENCE Phase 3 trial results demonstrating statistically significant and superior improvements with semaglutide 2.4 mg in people with MASH presented at AASLD 2024 - The Liver Meeting. Novo Nordisk USA. https://www.novonordisk-us.com/media/news-archive/news-details.html?id=171986. [Accessed 1 January 2025].
[19] Xie Z, Yang S, Deng W, et al. Efficacy and safety of liraglutide and semaglutide on weight loss in people with obesity or overweight: a systematic review. Clin Epidemiol 2022;14:1463–1476.
[20] Wilding JPH, Batterham RL, Davies M, et al. Weight regain and cardiometabolic effects after withdrawal of semaglutide: the STEP 1 trial extension. Diabetes Obes Metab 2022;24:1553–1564.
[21] Pramfalk C, Pavlides M, Banerjee R, et al. Sex-specific differences in hepatic fat oxidation and synthesis may explain the higher propensity for NAFLD in men. J Clin Endocrinol Metab 2015;100:4425–4433.
[22] Parry SA, Rosqvist F, Cornfield T, et al. Oxidation of dietary linoleate occurs to a greater extent than dietary palmitate in vivo in humans. Clin Nutr 2021;40:1108–1114.
[23] Smits MM, Tonneijck L, Muskiet MH, et al. Twelve week liraglutide or sitagliptin does not affect hepatic fat in type 2 diabetes: a randomised placebo-controlled trial. Diabetologia 2016;59:2588–2593.
[24] Cuthbertson DJ, Irwin A, Gardner CJ, et al. Improved glycaemia correlates with liver fat reduction in obese, type 2 diabetes, patients given glucagon-like peptide-1 (GLP-1) receptor agonists. PLoS One 2012;7:e50117.
[25] Tang A, Rabasa-Lhoret R, Castel H, et al. Effects of insulin glargine and liraglutide therapy on liver fat as measured by magnetic resonance in patients with type 2 diabetes: a randomized trial. Diabetes Care 2015;38:1339–1346.
[26] Loomba R, Hartman ML, Lawitz EJ, et al. Tirzepatide for metabolic dysfunction-associated steatohepatitis with liver fibrosis. N Engl J Med 2024;391:299. 10.
[27] Thoma C, Day CP, Trenell MI. Lifestyle interventions for the treatment of non-alcoholic fatty liver disease in adults: a systematic review. J Hepatol 2012;56:255–266.
[28] Khoo J, Hsiang JC, Taneja R, et al. Randomized trial comparing effects of weight loss by liraglutide with lifestyle modification in non-alcoholic fatty liver disease. Liver Int 2019;39:941–949.
[29] Khoo J, Hsiang J, Taneja R, et al. Comparative effects of liraglutide 3 mg vs structured lifestyle modification on body weight, liver fat and liver function in obese patients with non-alcoholic fatty liver disease: a pilot randomized trial. Diabetes Obes Metab 2017;19:1814–1817.
[30] Vedtofte L, Bahne E, Foghsgaard S, et al. One year's treatment with the glucagon-like peptide 1 receptor agonist liraglutide decreases hepatic fat content in women with nonalcoholic fatty liver disease and prior gestational diabetes mellitus in a randomized, placebo-controlled trial. J Clin Med 2020;9:3213.
[31] Shiomi M, Tanaka Y, Takada T, et al. Determining whether the effect of liraglutide on non-alcoholic fatty liver disease depends on reductions in the body mass index. JGH Open 2020;4:995–1001.
[32] Engström A, Wintzell V, Melbye M, et al. Association of glucagon-like peptide-1 receptor agonists with serious liver events among patients with type 2 diabetes: a Scandinavian cohort study. Hepatology 2024;79:1401–1411.
[33] Krishnan A, Schneider CV, Hadi Y, et al. Cardiovascular and mortality outcomes with GLP-1 receptor agonists vs other glucose-lowering drugs in individuals with NAFLD and type 2 diabetes: a large population-based matched cohort study. Diabetologia 2024;67:483–493.
[34] Ast J, Broichhagen J, Hodson DJ. Reagents and models for detecting endogenous GLP1R and GIPR. EBioMedicine 2021;74:103739.
[35] Matikainen N, Söderlund S, Björnson E, et al. Liraglutide treatment improves postprandial lipid metabolism and cardiometabolic risk factors in humans with adequately controlled type 2 diabetes: a single-centre randomized controlled study. Diabetes Obes Metab 2019;21:84–94.
[36] Kasher-Meron M, Youn DY, Zong H, et al. Lipolysis defect in white adipose tissue and rapid weight regain. Am J Physiol Endocrinol Metab 2019;317:E185–E193.
[37] van Baak MA, Mariman ECM. Obesity-induced and weight-loss-induced physiological factors affecting weight regain. Nat Rev Endocrinol 2023;19:655–670.
[38] Hjorth MF, Christensen L, Kjølbæk L, et al. Pretreatment Prevotella-to-Bacteroides ratio and markers of glucose metabolism as prognostic markers for dietary weight loss maintenance. Eur J Clin Nutr 2020;74:338–347.
[39] Kassim SY, Gharib SA, Mecham BH, et al. Individual matrix metalloproteinases control distinct transcriptional responses in airway epithelial cells infected with *Pseudomonas aeruginosa*. Infect Immun 2007;75:5640–5650.
[40] García-Irigoyen O, Latasa MU, Carotti S, et al. Matrix metalloproteinase 10 contributes to hepatocarcinogenesis in a novel crosstalk with the stromal derived factor 1/C-X-C chemokine receptor 4 axis. Hepatology 2015;62:166–178.
[41] Sze KM, Chu GK, Lee JM, et al. C-terminal truncated hepatitis B virus x protein is associated with metastasis and enhances invasiveness by C-Jun/matrix metalloproteinase protein 10 activation in hepatocellular carcinoma. Hepatology 2013;57:131–139.
[42] Hu X, Yang L, Yu W, et al. Association of serum fibroblast growth factor 23 levels with the presence and severity of hepatic steatosis is independent of sleep duration in patients with diabetes. Diabetes Metab Syndr Obes 2020;13:1171–1178.
[43] Hu X, Ma X, Luo Y, et al. Associations of serum fibroblast growth factor 23 levels with obesity and visceral fat accumulation. Clin Nutr 2018;37:223–228.
[44] Hang H, Yuan S, Yang Q, et al. Multiplex bead array assay of plasma cytokines in type 2 diabetes mellitus with diabetic retinopathy. Mol Vis 2014;20:1137–1145.
[45] Mastropasqua R, D'Aloisio R, Di Nicola M, et al. Relationship between aqueous humor cytokine level changes and retinal vascular changes after intravitreal aflibercept for diabetic macular edema. Sci Rep 2018;8:16548.
[46] Larsson A, Carlsson L, Lind AL, Gordh T, et al. The body mass index (BMI) is significantly correlated with levels of cytokines and chemokines in cerebrospinal fluid. Cytokine 2015;76:514–518.
[47] Ponce-de-Leon M, Linseisen J, Peters A, Linkohr B, et al. Novel associations between inflammation-related proteins and adiposity: a targeted proteomics approach across four population-based studies. Transl Res 2022;242:93–104.
[48] Liu Q, Cai BY, Zhu LX, et al. Liraglutide modulates gut microbiome and attenuates nonalcoholic fatty liver in db/db mice. Life Sci 2020;261:118457.
[49] Ying X, Rongjiong Z, Kahaer M, et al. Therapeutic efficacy of liraglutide versus metformin in modulating the gut microbiota for treating type 2 diabetes mellitus complicated with nonalcoholic fatty liver disease. Front Microbiol 2023;14:1088187.
[50] Shang J, Liu F, Zhang B, et al. Liraglutide-induced structural modulation of the gut microbiota in patients with type 2 diabetes mellitus. PeerJ 2021;9:e11128.
[51] Crovesy L, Masterson D, Rosado EL. Profile of the gut microbiota of adults with obesity: a systematic review. Eur J Clin Nutr 2020;74:1251–1262.
[52] Sakamoto Y, Yoshio S, Doi H, et al. Increased frequency of dysfunctional Siglec-7. Front Immunol 2021;12:603133.
[53] Dai W, Choubey M, Patel S, et al. Adipocyte CAMK2 deficiency improves obesity-associated glucose intolerance. Mol Metab 2021;53:101300.
[54] EASL–EASD–EASO Clinical Practice Guidelines on the management of metabolic dysfunction-associated steatotic liver disease (MASLD). J Hepatol 2024;81:492–542.

Keywords: MASLD; GLP-1; Liraglutide; Weight loss.
Received 25 October 2024; received in revised form 6 February 2025; accepted 11 February 2025; Available online 21 February 2025

Journal of Hepatology, Volume *7*

Supplemental information

Randomised trial comparing weight loss through lifestyle and GLP-1 receptor agonist therapy in people with MASLD

Ahmad Moolla, Toryn Poolman, Nantia Othonos, Jiawen Dong, Kieran Smith, Thomas Cornfield, Sarah White, David W. Ray, Sofia Mouchti, Ferenc E. Mózes, Helena Thomaides-Brears, Stefan Neubauer, Jeremy F. Cobbold, Leanne Hodson, and Jeremy W. Tomlinson

Randomised trial comparing weight loss through lifestyle and GLP-1 receptor agonist therapy in people with MASLD

Ahmad Moolla, Toryn Poolman, Nantia Othonos, Jiawen Dong, Kieran Smith, Thomas Cornfield, Sarah White, David W. Ray, Sofia Mouchti, Ferenc E. Mózes, Helena Thomaides-Brears, Stefan Neubauer, Jeremy F. Cobbold, Leanne Hodson, Jeremy W. Tomlinson

Table of contents

Supplementary methods……………………………………………………………….………2

Supplementary figures…………………………………………………………….…….31

Supplementary tables…………………………………………………………….…..34

References……………………………………………………………………………... 42

1. Supplementary methods

a. Clinical study protocol

ABBREVIATIONS

AE	Adverse event
AR	Adverse reaction
CI	Chief Investigator
CRF	Case Report Form
CRU	Clinical Research Unit
CT	Clinical Trials
CTA	Clinical Trials Authorisation
CTRG	Clinical Trials and Research Governance
DMC/DMSC	Data Monitoring Committee / Data Monitoring and Safety Committee
DSUR	Development Safety Update Report
DNL	De novo lipogenesis
GCP	Good Clinical Practice
GLP-1	Glucagon-like peptide 1
GP	General Practitioner
IB	Investigators Brochure
ICF	Informed Consent Form
ICH	International Conference on Harmonisation
IMP	Investigational Medicinal Product
IRB	Independent Review Board
MHRA	Medicines and Healthcare products Regulatory Agency
MRI/S	Magnetic resonance imaging/spectroscopy
NAFLD	Non-alcoholic fatty liver disease
NASH	Non-alcoholic steatohepatitis
NHS	National Health Service
NRES	National Research Ethics Service
PI	Principal Investigator
PIL	Participant/ Patient Information Leaflet
R&D	NHS Trust R&D Department
REC	Research Ethics Committee

SAE	Serious Adverse Event
SAR	Serious Adverse Reaction
SDV	Source Data Verification
SMPC	Summary of Medicinal Product Characteristics
SOP	Standard Operating Procedure
SUSAR	Suspected Unexpected Serious Adverse Reactions
TMF	Trial Master File
TSG	Oxford University Hospitals Trust / University of Oxford Trials Safety Group
T2DM	Type II Diabetes Mellitus
13C	13C Labelled Isotope

OBJECTIVES AND OUTCOME MEASURES

**Objectives**		**Outcome Measures**	**Timepoint(s) of evaluation**
**Primary**	To determine whether the benefits of Liraglutide treatment in NASH are independent of weight loss	Fat and inflammation measured using multi-parametric MR imaging (abdominal MRI)	At scanning day 1 (baseline) and scanning day 2 (after 12 weeks of treatment) and at scanning day 3 (12 weeks after treatment withdrawal)
**Secondary**	Change in rate of hepatic de-novo lipogenesis	Measurement of incorporation of $^{2}H_2$ palmitate from $^{2}H_2O$ into very low density lipoprotein (VLDL) triglyceride using gas chromatography mass spectrometry (GCMS).	At study day 1 (baseline) and at study day two (after 12 weeks of treatment) and at study day 3 (12 weeks after treatment withdrawal)
	Change in circulating liver transaminases	Liver function blood testing	Baseline bloods at screening and consent visit, at

			intermediate visit and at study day 2 and 3.
	Change in hepatic and peripheral insulin sensitivity and glucose disposal	Glucose disposal and insulin secretion across a prolonged oral glucose tolerance test incorporating measurement of glucose fate using ^{13}C-glucose	At study day 1 (baseline) and at study day two (after three months of treatment) and at study day 3 (12 weeks after treatment withdrawal)
	Change in adipose tissue insulin sensitivity	Adipose microdialysate analysis and adipose tissue gene expression and as part of prolonged oral glucose tolerance tests.	At study day 1 (baseline) and at study day two (after three months of treatment) and at study day 3 (12 weeks after treatment withdrawal)
	Change in whole body oxidation	Measurement of incorporation of ^{13}C in breath CO_2 using GC-C-IRMS.	At study day 1 (baseline) and at study day two (after three months of treatment) and at study day 3 (12 weeks after treatment withdrawal)
	Change in BMI and general health	Serial BMI measurements, diet review, and exercise data (pedometer or accelerometer), blood tests, blood pressure, urine and faecal composition	Baseline measurements at screening and consent, and then at intermediate visits and at study days 2 and 3.
	Change in body composition	Adipose tissue mass and distribution measured using dual energy x-ray absorptiometry (DXA)	At study day 1 (baseline) and at study day two (after three months of treatment) and at study day 3 (12 weeks

			after treatment withdrawal)
	Change in urinary markers of liver disease	Urinary metabolites	At study day 1 (baseline) and at study day two (after three months of treatment) and at study day 3 (12 weeks after treatment withdrawal)
	Change in faecal markers of liver disease	Faecal metabolites and microbiome	At study day 1 (baseline) and at study day two (after three months of treatment) and at study day 3 (12 weeks after treatment withdrawal)
	Sustained effect of GLP-1 or weight loss intervention on liver inflammation	Multi-parametric MR scan (abdominal MRI)	24 weeks from baseline (12 weeks after treatment withdrawal)

TRIAL DESIGN

We will conduct an open label, randomized phase IIa study to evaluate the effect of Liraglutide compared to lifestyle induced weight loss on liver inflammation (non-alcoholic steatohepatitis, NASH).

Participants with NASH will be randomised to a Lifestyle intervention programme arm or to a Liraglutide treatment arm. Both arms will complete 12 weeks of the therapy to which they are randomised and will undergo identical investigations at baseline, at 12 weeks (completion of treatment) and at a further follow-up visit at 24 weeks.

This programme of work will elucidate whether the beneficial effects of Liraglutide seen in patients with NASH are mediated via weight loss only or via other beneficial effects.

**Visit**	**Purpose**	**Duration**

**Screening and Consent**	Screening and consent<br><br>Initial investigations including height, weight<br><br>Baseline blood tests, urine and faeces sample<br><br>Complete exercise questionnaire	1 hour
**Scanning Day 1**	Liver and abdominal MRI scan<br><br>Instructions for study day 1 including how to drink heavy water and sample containers provided to collect urine and faeces samples.	1 hour
**Study Day 1**	Investigations including glucose tolerance test, DXA scan, fat tissue sampling and biopsy, regular blood and breath tests.<br><br>Bring previous week's pedometer and food diary data to visit.	4 hours
	**Randomisation to either liraglutide or lifestyle intervention arms at the end of Study Day 1**	
**Intermediate Visit 1 (Week 1)**	Weight, blood test, chance to answer questions	1 hour
**Intermediate Visit 2 (Week 4)**	Weight, blood test, chance to answer questions	1 hour
**Intermediate Visit 3 (Week 8)**	Weight, blood test, chance to answer questions	1 hour
**Scanning Day 2**	Liver and abdominal MRI scan	1 hour

	Instructions for study day 2 including how to drink heavy water and sample containers provided to collect urine and faeces samples.	
**Study Day 2**	Investigations including glucose tolerance test, DXA scan, fat tissue sampling and biopsy, regular blood and breath tests.<br>Bring previous week's pedometer and food diary data to visit.<br>Complete exercise questionnaire.	4 hours
**Scanning Day 3**	Liver and abdominal MRI scan<br>Instructions for study day 3 including how to drink heavy water and sample containers provided to collect urine and faeces samples.	1 hour
**Study Day 3**	Investigations including glucose tolerance test, DXA scan, fat tissue sampling and biopsy, regular blood and breath tests<br>Bring previous week's pedometer and food diary data to visit.<br>Complete exercise questionnaire.	4 hours

PARTICIPANT IDENTIFICATION

Trial Participants

This trial aims to recruit obese patients with biopsy proven non-alcoholic fatty liver disease (non-alcoholic steatohepatitis, NASH), but without type 2 diabetes

25 volunteers will be randomized to Lifestyle induced weight loss programme or Liraglutide treatment

Inclusion Criteria

The following criteria apply to both arms and to each volunteer who has a successful screening visit will be randomised to one of these arms:

- Non-diabetic (HbA1c <48mmol/mol)
- BMI 25-40kg/m^2
- Age 18-75years
- Diagnosis of NAFLD (hepatic steatosis on imaging or histology)
- ALT ≥ 1.5 ULN & <10 ULN on 2 occasions >7 days apart and / or

- Historical liver biopsy showing NASH and/or ≥F1 fibrosis or NFS ≥ -1.455 OR Fib-4 ≥ 1.3 OR Fibroscan ≥8kPa.
- Non-pregnant, not planning pregnancy
- BP<160/100mmHg – stable antihypertensive therapy for 6 months
- No weight change >10% within the preceding 3 months
- Normal renal function
- No contraindications to Lifestyle or Liraglutide

Exclusion Criteria

The participant may not enter the study if ANY of the following apply:

- Age <18 or >75years
- Body mass index <25 or >40kg/m^2
- A diagnosis of diabetes (type 1 or type 2)
- Use of anti-diabetic or weight loss medications or a GLP-1 agonist such as Liraglutide)
- Contra-indication to Liraglutide
- A blood haemoglobin <120mg/dL
- History of alcoholism or a greater than recommended alcohol intake (Recommendations > 21 drinks on average per week for men and > 14 drinks on average per week for women
- Pregnant or nursing mothers
- History of severe claustrophobia
- Presence of metallic implants, pacemaker that are contra-indications to MRI scanning
- Haemorrhagic disorders
- Anticoagulant treatment
- Other co-morbidities that in the eyes of the investigators may affect data collection
- Any medical condition in the opinion of the investigator that might impact upon safety or validity of the results

TRIAL PROCEDURES

Recruitment

Subjects will be recruited from local advertisements placed within the Oxford University Hospitals and GP surgeries, as well as relevant liver disease clinics at Oxford University Hospitals NHS Foundation Trust, for example the NAFLD clinic run jointly by Professor J Tomlinson and Dr J Cobbold. Potential participants from these clinics will be identified through search of relevant liver disease databases and contacted by letter and followed up with a telephone call or in person during relevant outpatient clinics.

Adverts may also be placed on social and other electronic media such as Twitter and Facebook. Participants who fulfil the inclusion criteria and who are interested in being part of the study will be

provided with an information leaflet and given at least 24-hours to consider participation in the study. The participants will be contacted via phone to answer any potential questions prior to attendance at the screening and consent visit.

Screening and Eligibility Assessment

Volunteers and participants will be seen in the Oxford Centre for Diabetes Endocrinology and Metabolism (OCDEM) or in the NALFD clinic (blue outpatients at the John Radcliffe Hospital) to determine their suitability for the study and to answer any questions. If they agree to be part of the study, informed written consent will be taken. The participant will then have a medical history taken and physical medical examination, baseline height and weight measurements, blood pressure, baseline blood tests including for genetic testing and bioimpedance analysis to assess body water and fat composition. Urine and stool samples will be collected. We will ask women of childbearing age to sign a statement confirming they are not pregnant and in cases of uncertainty we would offer a pregnancy test or withdrawal from the study.

All participants will be provided with an International Physical Activity Questionnaire (IPAQ) at screening to be completed prior to each study day and also provided with a pedometer (or accelerometer) to monitor their daily exercise levels with the aim of achieving 10,000 steps per day as recommended in national NHS guidance.

Informed Consent

The participant must personally sign and date the latest approved version of the Informed Consent form before any study specific procedures are performed. Only the investigators named above in section 2 or their appropriately trained deputies will consent participants. Consent will be taken at the *screening and consent visit*. An additional appointment will be scheduled to re-discuss the study prior to taking consent if needed.

Written and verbal versions of the Participant Information and Informed Consent will be presented to the participants detailing no less than: the exact nature of the study; what it will involve for the participant; the implications and constraints of the protocol; the known side effects and any risks involved in taking part. It will be clearly stated that the participant is free to withdraw from the study at any time for any reason without prejudice to future care, and with no obligation to give the reason for withdrawal.

The participant will be allowed as much time as wished to consider the information, and the opportunity to question the Investigator, their GP or other independent parties to decide whether they will participate in the study. Written Informed Consent will then be obtained by means of participant dated signature and dated signature of the person who presented and obtained the Informed Consent. The person who obtained the consent must be suitably qualified and experienced, and have been authorised to do so by the Chief/Principal Investigator. A copy of the signed Informed Consent will be given to the participant. The original signed form will be retained at the study site.

The maximum duration between screening and participation will be 8 weeks. As long as participants fulfil the inclusion criteria they will be eligible.

Randomisation, blinding and code-breaking

The study is not blinded. A randomisation schedule will be drawn up by an independent investigator (to avoid bias) not linked to the study via an electronic Excel based randomization tool. Randomization will be in blocks of 6 patients (3 to Lifestyle intervention and 3 to Liraglutide)

Baseline Assessments

Baseline assessments at screening and consent visit:

- Height and weight measurements,
- Baseline blood tests (including full blood count, renal profile and liver function tests and for genetic testing for genes related to NAFLD)
- Bioimpedance analysis to assess body water and fat composition
- Urine and stool sample
- IPAQ questionnaire and 24hr pedometer data

Baseline assessment at scanning day 1 and study day 1, before Liraglutide or lifestyle intervention:

- Hepatic steatosis / inflammation as measured by abdominal magnetic resonance imaging / liver multi scan (scanning day 1)
- De novo lipogenesis measurement by of incorporation of $^{2}H_{2}$ palmitate from $^{2}H_{2}O$ into very low density lipoprotein (VLDL) triglyceride using gas chromatography mass spectrometry (GCMS).
- Liver function and other blood testing, urine and stool collection
- Hepatic insulin sensitivity (triglyceride suppression) and glucose disposal and utilization as measured across a prolonged oral glucose tolerance test
- Adipose tissue insulin sensitivity as measured by adipose microdialysate analysis and adipose tissue gene expression (from biopsy)
- Whole body glucose oxidation measurement by incorporation of ^{13}C in breath CO_2 using GC-C-IRMS.
- Body composition as measured by dual energy absorptiometry (DXA)

Subsequent visits and study procedures

Scanning Day 1

After the screening visit, participants satisfying the inclusion criteria will be invited to come for magnetic resonance imaging where we will measure liver and abdominal fat content, inflammation and fibrosis. This will be done at the University of Oxford Centre for Clinical Magnetic Resonance

Research (OCMR) at the John Radcliffe Hospital site (Appendix A 19.1). Patients will have been asked to fast for 4 hours prior to the scan. After the MRI scan, participants will be provided with deuterated 'heavy' water and given clear verbal and written instructions on how to take this the evening before attending for study day 1 (See Study Day Preparation Sheet). Participants will be asked to avoid excessive exercise, alcohol and foods naturally enriched with ^{13}C (e.g cornflakes) for at least 24 hours prior to the Study Day. Participants will be reminded to fast from 8pm the night before their study day.

Study Day 1 (within 1 week of Scanning Day 1,)

Within 1-week of the MRI scan, participants will be invited to a study day at the Clinical Research Unit (CRU) at OCDEM beginning at 0800.

Simple measurements including, blood pressure and weight will be performed. Additionally, an adipose tissue mass and distribution will be measured using dual energy x-ray absorptiometry (DXA scan, Appendix A 19.2). A urine sample and faecal sample (if possible) will also be collected which will allow for metabolomic assessment of the participant's NAFLD.

To assess hepatic fatty acid synthesis, participants will consume 2 pre-specified doses of deuterated 'heavy water' ($^{2}H_2O$ (CK Gas Ltd)) the evening prior to the study day at 1800 and 2200 respectively. The dose (volume) of heavy water to be consumed at 1800 and 2200 will be calculated as follows:

- *Volume of heavy water (ml) = [(total body water (kg) X 3) / 2]*

Furthermore, from 1800 onwards until the end of the study day visit, participants will be provided with and asked to drink water enriched with deuterated water at a concentration of 4g/l to be drunk as required in lieu of any other fluids. Clear instructions on how to take the doses and mobile numbers for researchers will have been given to the participants at their scanning day 1. This system has worked well in previous studies and negates the need for participants to attend another visit to pick up heavy water.

On the study day, an adipose tissue microdialysis catheter will be inserted under local anaesthetic into the abdominal subcutaneous adipose tissue to measure adipose tissue specific effects (Appendix A 19.3) and samples taken every 30 minutes. An adipose tissue biopsy from the subcutaneous abdominal depot will be performed for gene expression analysis that will complement the dynamic assessments of metabolic function. (Appendix A 19.4)

A single venous cannulae will be inserted into the dorsum of the hand for blood sampling. The hand will be kept warmed using a heated blanket. A 3-way tap with extension tubing will be attached. We will ensure that blood can be taken easily from this cannula. 0.9 % NaCl will be infused slowly into the blood sampling cannula via a standard giving set and pump to maintain the patency of the cannula. Baseline bloods will be taken prior to starting any infusions.

Baseline blood tests will be taken (t=0 min) for measurements including background 13C-glucose enrichment as well as insulin, non-esterified fatty acids, liver chemistry and lipid profiles. A baseline breath sample will also be taken. An oral glucose load (75g glucose + 0.5g ^{13}C-glucose) will be dissolved in water and administrated orally. Additional breath and blood samples will be taken at t=5, 15, 30, 60, 90, 120, 150 and 180minutes for measurement including glucose, insulin, NEFA and lactate. (Appendix A 19.5).

Once the glucose tolerance test has been completed, the cannula will be removed and the participant can eat and drink and will be provided with a meal. A taxi will be arranged for the participant to go home.

Participants will be randomized to either the Lifestyle intervention programme or Liraglutide treatment at the end of Study Day 1. For those randomised to Liraglutide, this will be prescribed by the trial investigators and dispensed by the Oxford University Hospitals NHS Trust Pharmacy for the duration of therapy. The Liraglutide will be up-titrated stepwise as per routine usual clinical practice to a dose of 1.8mg over a period of 2 weeks (see schematic, section 6) by the trial investigators. For those undergoing the lifestyle intervention programme, for participant convenience, this intervention will be a commercially available programme (Weight Watchers, Slimming World or similar) based within the patient's home locality.

Intermediate Visits - Weeks 1, 4 and 8

Participants will return for intermediate visits at 1, 4 and 8 weeks for review, blood tests and to undergo basic investigations. They will have the chance to report back on any challenges they are facing during the intervention phase including a check on compliance with therapy or intervention. They will be given the opportunity to ask any questions and to ensure that they remain in good health. For those on Liraglutide treatment, injection sites will be checked to ensure no local complications and participants will be asked about injection techniques to ensure correct administration. Investigations will include weight, blood pressure, bioimpedance analysis, full blood count, renal and liver function tests.

Scanning Day 2 (performed at 12 weeks +/- 3 days)

Participants will undergo identical assessment to that described for scanning day 1.

Study Day 2 (performed at 12 weeks +/- 3 days, Appendix B)

Participants will undergo identical assessment to that described in study day 1 including adipose tissue biopsy and microdialysis, prolonged oral glucose tolerance test, DXA body composition analysis. In the Liraglutide arm of the study, Liraglutide will have been taken on the morning of the investigations.

The participant will stop the Liraglutide or Lifestyle intervention after these investigations at 12 weeks.

Scanning Day 3 (performed at 24 weeks +/- 3 days)

Participants will undergo identical assessment to that described for scanning day 1.

Study Day 3 (performed at 24 weeks +/- 3 days, Appendix B)

Participants will undergo identical assessments to that described in study day 1 including adipose tissue biopsy and microdialysis, prolonged oral glucose tolerance test, DXA body composition analysis.

Prolonged oral glucose tolerance test (Study day protocol)

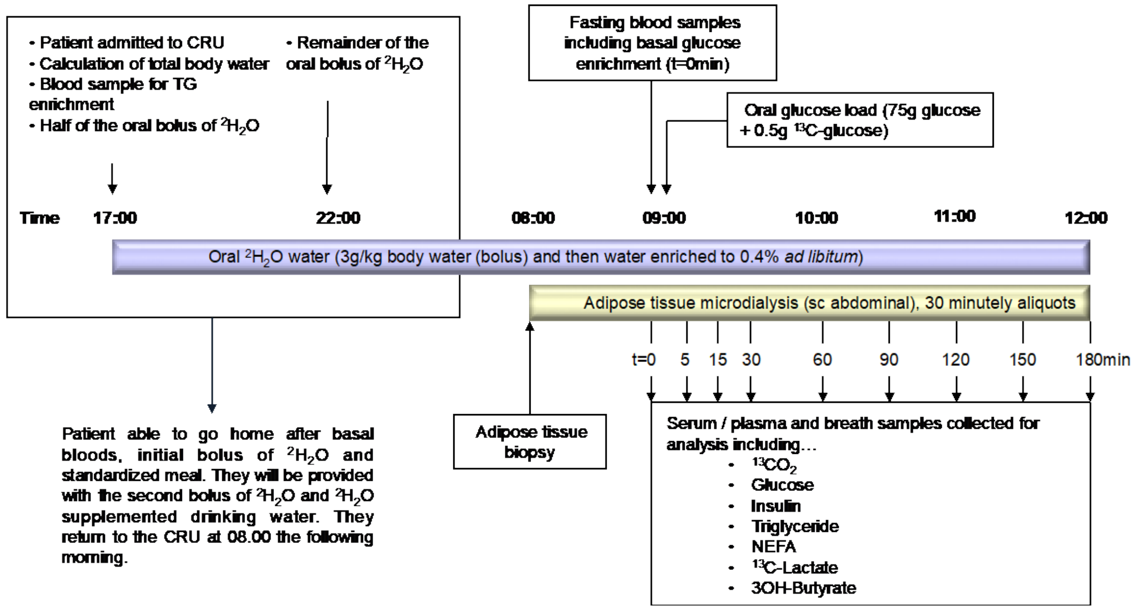

Sample Handling

All samples will be collected at the screening and consent visit, study day 1, intermediate visits and study days 2 and 3.

Routine bloods (cholesterol profile, HbA1C, LFTs, FBC, Renal profile) will be sent to laboratory in the Churchill Hospital for processing on the same day of sampling. Samples for other blood tests including insulin (calculation of isotope glucose) and genetic testing, adipose tissue biopsies and microdialysis samples will be stored in a -80C freezer in the research laboratory in the OCDEM building until analysis

The blood volume that will be collected per visit is approximately 180mls for study days 1, 2 and 3, and 20ml for each intermediate visit and screening. Over the duration of the study, approximately 620ml of blood will be collected. For the adipose tissue microdialysis samples, the volumes collected per sample will be < 1mL. For adipose tissue biopsies, the amount collected will be similar to half a little finger nail for each of two biopsies.

All human samples will be securely stored throughout the duration of the study. At the end of the research, samples and biological material which are not “relevant material” for the purposes of the Human Tissue Act will be stored for up to 10 years. All other samples and biological materials will

be disposed of in accordance with guidelines. Any samples stored after the research has ended may be made available for future research testing but only after having obtained appropriate informed consent from participants.

Members of Professor Jeremy Tomlinson's laboratory will have access to the freezers where the samples will be stored. They will also help process the experiments/studies on the samples. Access to the research laboratory and freezers is secure and only accessible with an accredited swipe card issued from the University. With participant consent, samples may be transferred to collaborators outside of the University of Oxford, including collaborators outside of the UK. The samples will only contain subject numbers and will contain no personal details for the participant which would link them to the sample.

Discontinuation/Withdrawal of Participants from Trial Treatment

Each participant has the right to withdraw from the study at any time. In addition, the Investigators may discontinue a participant from the study at any time if the Investigators considers it necessary for any reason including:

- Ineligibility (either arising during the study or retrospectively having been overlooked at screening)
- Significant protocol deviation
- Significant non-compliance with drug regimen or study requirements
- Withdrawal of consent
- Loss to follow up

The reason for withdrawal will be recorded in the CRF. If the participant is withdrawn due to an adverse event, the Investigator will arrange for follow-up visits or telephone calls until the adverse event has resolved or stabilised and with the participant's consent, inform their GP.

Definition of End of Trial

The end of study is the end of study day 3 at 24 weeks for the last participant.

INVESTIGATIONAL MEDICINAL PRODUCT (IMP)

1. ***IMP Description***

Name of IMP: Liraglutide (Victoza)

Dosage: To start at 0.6mg daily and titrated weekly by 0.6mg up to a final dose of 1.8mg daily (This is identical to how Liraglutide is commenced and dosed when used in routine clinical practice)

Route of administration: Subcutaneous injection (as per routine clinical practice)

Dosage form, packaging, and labelling: As per routine clinical practice (this study is not blinded)

NB: Liraglutide also has a European wide licence for use in patients with obesity at a higher dose of 3mg (Saxenda). As trial participants are obese, they would be eligible for Liraglutide therapy irrespective of this trial.

Storage of IMP

Liraglutide will be dispensed from the Hospital pharmacy and patients will be advised how to store this medication safely and securely at home as per all routine guidance for Liraglutide therapy when used in clinical practice.

Compliance with Trial Treatment

Compliance with Liraglutide will be assessed via discussion with participants during the intermediate study visits and documented in the participants' study files. Participants will asked to keep a diary card with the Liraglutide and bring this to study visits. Compliance with intervention will be defined as at least 80% of doses being administered. At the end of the intervention period, participants will be asked to bring all unused or part-used medication/vials and packaging for checking and safe disposal.

Accountability of the Trial Treatment

All participants recruited will undergo either Liraglutide treatment or a Lifestyle intervention programme to achieve weight loss dependent on local availability of the programme. Both interventions are routinely used in clinical practice to promote weight loss and are known to be both safe and effective. Based on previously published data, these interventions have been specifically designed to allow participants to achieve approximately a 5% loss of body weight at 12 weeks from commencement.

Liraglutide

Liraglutide is administered via a subcutaneous injection once daily. Participants in the Liraglutide arm will be taught in person via demonstration how to safely prepare and safely self-inject the medication at the same time each day by an experienced health professional from our Clinical Research Facility to ensure they are competent and confident in doing so independently. Participants will be warned about the potential adverse side effects (mainly transient gastrointestinal disturbances). If side effects do occur, volunteers will be asked to contact the study investigators to determine a plan of action such as to reduce the dose to the maximum dose tolerated without significant adverse effects. In the unlikely event that they are unable to tolerate any dose of Liraglutide, they will be withdrawn from the study.

Liraglutide will be prescribed for a minimum of 1 month to coincide with each follow-up visit and will be re-prescribed during the follow-up intermediate study visits as required. This will ensure regular and timely monitoring and an opportunity to check compliance.

Lifestyle Intervention Programme / Commercial Weight Loss Programmes

There is good evidence that commercial weight loss programmes for those who are overweight are effective in achieving weight loss, whilst also being cost-effective for health services. (Hartmann-Boyce, 2014). Programmes that have been studied in the UK include those run by Weight Watchers and Slimming World and interestingly, these appear to promote greater weight loss than services run directly within primary care. These programmes employ structured education methods to promote changes in lifestyle incorporating changes to diet, exercise advice and providing behavioural counselling to achieve weight loss. Regular follow-up, ideally weekly, also appears to be important with adequate time provided to allow meaningful interactions and where opportunity for group based therapy may also exist.

For participants in the Lifestyle arm to achieve the 5% weight loss desired at 12 weeks, they will be enrolled into a commercial weight loss programme which employs a combinations of the methods (diet, exercise and behaviour) as outlined above. The precise programme chosen will depend on availability in each participants' local area to help ensure convenience and compliance.

No serious adverse effects are anticipated in the Lifestyle intervention arm though participants will be regularly monitored both by the weight loss programme (weekly) and will also have access to the study investigators (by phone and email) as required should the need arise. Additionally, the intermediate study visits at week 1, 4 and 8 are also partly designed to help identify any challenges participants may be facing and to assist as required.

Concomitant Medication

Contraindicated medications will include any weight loss or anti-diabetic medication. See inclusion, exclusion and withdrawal criteria.

Post-trial Treatment

There will not be provision of the IMP beyond the trial period.

SAFETY REPORTING

As this is a research study, it will be explained to participants that the research intention does not directly focus on improving their individual health. Nonetheless, it is very likely that participants in both arms may well gain health benefits from partaking in the study as weight loss is the mainstay of treatment for NASH.

Potential risks and side effects of the interventions, particularly to those commencing Liraglutide, will be explained to participants prior to commencing therapy. Serious adverse events will be recorded by the investigators as part of the study. Liraglutide is widely used in routine clinical practice to treat both obesity and diabetes and is generally well tolerated and safe. Possible side effects that may be anticipated in advance from Liraglutide treatment include transient gastrointestinal disturbance, rash and hypersensitivity reactions.

Whilst all serious adverse events will be reported, these may not be necessarily related to Liraglutide or the Lifestyle intervention programme. All studies will be performed in a Clinical Research Unit fully equipped for dealing with emergencies. There is a potential for clotting or infection associated with the indwelling cannulae but again this can be minimised by good clinical practice and we have never observed such effects in many hundreds of metabolic studies.

Venepuncture for the screening sample may cause momentary discomfort. Insertion of a venous cannula under local anaesthetic on the study day normally only causes momentary discomfort because of the 'sting' of the local anaesthetic. Cannulation sites may bruise and there is a possible risk for clotting or infection. We will minimise these risks by the use of good clinical practice and sterile techniques.

The evening prior to and during the study days, we are asking the participants to drink 'heavy water' (water containing stable isotope). With the consumption of 'heavy water' there is a possibility that participants may experience transient dizziness which has been attributed to a perturbation of the specific gravity of vestibular fluid. To help avoid this, we will split the dose in two portions, to be taken 2 hours apart. During the overnight phase and over the course of the study day, the heavy water will be in a dilute form (mixed with filtered water) and it is highly unlikely that this will cause any perturbation to the specific gravity of vestibular fluid. We wish to stress that the use of the stable isotopes in small doses is completely harmless in terms of negative health effects and it is NOT radioactive.

The total amount of blood taken during the study day will be at maximum 180ml, approximately half what might be given at a normal blood donation. It is safe to lose this amount of blood during the study day and there are no adverse effects to expect from this procedure. At the screening visit and on each subsequent study day we will assess the volunteer's haemoglobin status to ensure there is no anaemia. We will as a precaution advise participants to refrain from blood donation prior to and for 90 days after the study. During the course of the study day we will ensure participants stay well hydrated and before leaving the CRU at the end of the study we will ensure that they have had a light meal. We will also provide advice regarding the 24-48 hour period after the study to ensure that they consume sufficient non-alcoholic fluid, food and do not undertake any strenuous exercise or activities.

The dual energy x-ray absorptiometry (DXA) scans planned on the 3 study days will involve exposure to a small radiation dose. These scans use a much lower level of radiation than a standard X-ray examination – less than two days' exposure when compared to natural background radiation (NBR) already present in the general environment. By comparison, a chest X-ray uses the equivalent of about five days' exposure to NBR, and a flight to North America is equivalent to about a week's exposure to NBR. As such, these scans are very safe procedures and scan operators can also remain within the same environment as the participant. As radiation exposure

can cause harm to the unborn foetus, female participants will be asked to sign a statement confirming they are not pregnant at screening and on the study day. In cases of uncertainty, we would offer a pregnancy test.

MRI is a safe and non-invasive technique, which does not involve ionising radiation. Risks associated with the magnetic field will be removed by excluding potential participants with ferromagnetic objects in their bodies (e.g. metal implants, vessel clips, shrapnel injuries) or with implanted devices which may be damaged by the magnet (e.g. heart pacemakers). All people entering the scanner room will be screened for such objects. While most people do not experience discomfort in a MRI environment, the enclosed space of the scanner can potentially feel uncomfortable, especially for more elderly subjects. Discomfort from lying still for a long period of time will be minimised with comfortable padding and positioning. Whilst in the scanner, subjects would be able to use the alarm button if they wish to communicate with the operator or to interrupt the scanning. People with a history of claustrophobia would be excluded from participation in the study. All participants would be introduced carefully to the scanner and allowed to leave at any stage, should they wish to do so. Once in the scanner, participants will be able to indicate immediately if they wish the scanning to cease by squeezing a bulb placed in their hands, or by requesting it verbally. As the MR scanner is noisy, subjects would be given ear-plugs and acoustically shielded headphones with advice on how to wear these to minimise the noise.

The stable isotopes, including the heavy water, will be obtained from CK Gas Ltd. Any stable isotope that we introduce directly into the blood will have been sterility and pyrogenicity tested.

Definitions

Adverse Event (AE)	Any untoward medical occurrence in a participant to whom a medicinal product has been administered, including occurrences which are not necessarily caused by or related to that product.
Adverse Reaction (AR)	An untoward and unintended response in a participant to an investigational medicinal product which is related to any dose administered to that participant.<br>The phrase "response to an investigational medicinal product" means that a causal relationship between a trial medication and an AE is at least a reasonable possibility, i.e. the relationship cannot be ruled out.<br>All cases judged by either the reporting medically qualified professional or the Sponsor as having a reasonable suspected causal relationship to the trial medication qualify as adverse reactions.

Serious Adverse Event (SAE)	A serious adverse event is any untoward medical occurrence that:<br>• results in death<br>• is life-threatening<br>• requires inpatient hospitalisation or prolongation of existing hospitalisation<br>• results in persistent or significant disability/incapacity<br>• consists of a congenital anomaly or birth defect.<br>Other ‘important medical events’ may also be considered serious if they jeopardise the participant or require an intervention to prevent one of the above consequences.<br>NOTE: The term "life-threatening" in the definition of "serious" refers to an event in which the participant was at risk of death at the time of the event; it does not refer to an event which hypothetically might have caused death if it were more severe.
Serious Adverse Reaction (SAR)	An adverse event that is both serious and, in the opinion of the reporting Investigator, believed with reasonable probability to be due to one of the trial treatments, based on the information provided.
Suspected Unexpected Serious Adverse Reaction (SUSAR)	A serious adverse reaction, the nature and severity of which is not consistent with the information about the medicinal product in question set out:<br>• in the case of a product with a marketing authorisation, in the summary of product characteristics (SmPC) for that product<br>• in the case of any other investigational medicinal product, in the investigator’s brochure (IB) relating to the trial in question.

NB: to avoid confusion or misunderstanding of the difference between the terms “serious” and “severe”, the following note of clarification is provided: “Severe” is often used to describe intensity of a specific event, which <u>may</u> be of relatively minor medical significance. “Seriousness” is the regulatory definition supplied above.

Any pregnancy occurring during the clinical trial and the outcome of the pregnancy should be recorded and followed up for congenital abnormality or birth defect, at which point it would fall within the definition of “serious”.

Causality

The relationship of each adverse event to the trial medication must be determined by a medically qualified individual according to the following definitions:

Related: The adverse event follows a reasonable temporal sequence from trial medication administration. It cannot reasonably be attributed to any other cause.

Not Related: The adverse event is probably produced by the participant's clinical state or by other modes of therapy administered to the participant.

Procedures for Recording Adverse Events

The IMP's (Liraglutide) side effects are well known under its clinical licences and it is generally well tolerated. As such only serious adverse events will be recorded during the study and non-serious adverse events will not be collected. All serious adverse events occurring during the trial that are observed by the Investigators or reported by the participant, will be recorded on the CRF, whether or not attributed to trial medication.

The following information will be recorded: description, date of onset and end date, severity, assessment of relatedness to trial medication, other suspect drug or device and action taken. Follow-up information should be provided as necessary.

The severity of events will be assessed on the following scale: 1 = mild, 2 = moderate, 3 = severe.

AEs considered related to the trial medication as judged by a medically qualified investigator or the Sponsor will be followed either until resolution, or the event is considered stable.

It will be left to the Investigator's clinical judgment to decide whether or not an AE is of sufficient severity to require the participant's removal from treatment. A participant may also voluntarily withdraw from treatment due to what he or she perceives as an intolerable AE. If either of these occurs, the participant must undergo an end of trial assessment and be given appropriate care under medical supervision until symptoms cease, or the condition becomes stable.

Reporting Procedures for Serious Adverse Events

All SAEs (other than those defined in the protocol as not requiring reporting) must be reported on the SAE reporting form to CTRG/R&D within 24 hours of the Site Study Team becoming aware of the event. CTRG/R&D will perform an initial check of the report, request any additional information, and ensure it is reviewed by the Medical Monitor on a weekly basis. It will also be reviewed at the next Trial Safety Group meeting. All SAE information must be recorded on an SAE form and faxed, or scanned and emailed, to CTRG/R&D. Additional and further requested information (follow-up or corrections to the original case) will be detailed on a new SAE Report Form and faxed/emailed to CTRG/R&D.

Expectedness

Expectedness will be determined according to the Summary of Product Characteristics for Liraglutide.

SUSAR Reporting

All SUSARs will be reported by the CI to the relevant Competent Authority and to the REC and other parties as applicable. For fatal and life-threatening SUSARS, this will be done no later than

7 calendar days after the Sponsor or delegate is first aware of the reaction. Any additional relevant information will be reported within 8 calendar days of the initial report. All other SUSARs will be reported within 15 calendar days.

Safety Monitoring Committee

The Oxford University Hospitals Trust / University of Oxford Trials Safety Group (TSG) will conduct a review of all SAEs for the trial during the intervention period and for 30 days after this is complete. The aims of this committee include:

- To pick up any trends, such as increases in un/expected events, and take appropriate action
- To seek additional advice or information from investigators where required
- To evaluate the risk of the trial continuing and take appropriate action where necessary

Development Safety Update Reports

The CI will submit (in addition to the expedited reporting above) DSURs throughout the clinical trial, or on request, to the Competent Authority (MHRA in the UK), Ethics Committee, Host NHS Trust and Sponsor.

STATISTICS

This study has undergone an independent statistical review by Ms Ruth Coleman who is independent of the study investigators to ensure it is adequately structured and powered to achieve meaningful results.

Description of Statistical Methods

Paired t tests or nonparametric equivalents will be used to compare outcomes before and after individual treatments. Unpaired t-tests will be used to test across and compare interventions. Where multiple comparisons are made, we will use repeated measures ANOVA analysis to determine the effects on each intervention.

The Number of Participants

Lifestyle intervention: A wide range of trials have been published which show that commercial lifestyle and weight loss programmes are successful at assisting participants in achieving weight loss. 5% weight loss is generally accepted as both an achievable and significant amount to bring about health benefits. Both Jebb et al (2011) and Jolly et al (2011) reported large trials investigating weight loss treatments and in their findings showed that commercial weight loss programmes are able to promote a 5% reduction in weight at 12 weeks.

Liraglutide intervention: Liraglutide is used in routine clinical practice to treat both obesity and diabetes. Previously published studies have shown that a dose of 1.8mg of Liraglutide daily is able

to decrease weight by approximately 5% (n=90) (Astrup, 2009). Furthermore, one of our recent studies (Armstrong, 2015) performed in a cohort of 7 volunteers who received Liraglutide 1.8mg daily for 12 weeks showed a similar weight loss of around 5%.

This study has thus been carefully designed to achieve an approximately equal percentage (5%) of weight loss in both the Liraglutide and lifestyle arms to allow a direct comparison of the benefits they provide in the treatment of NASH, and in particular to answer the question of whether the beneficial effects of Liraglutide to treat NASH are independent or additional to the benefits of weight loss.

A sample size of n=25 (to account for a 10% drop-out rate, incomplete sample collection, accurate matching of weight loss, sample loss during analysis), will allow us to observe a minimum of 20% reduction in liver fat as measured by MR imaging, based on weight loss of 5% at 12 weeks.

When considering our metabolic studies, these sample sizes will also ensure sufficient statistical power as based on our previous experiences of (Armstrong, 2015). This study, examining the impact of GLP-1 analogue therapy *vs.* placebo in volunteers with NAFLD (n=7 in each arm) used very similar metabolic variables that we plan to use. Rates of *de novo* lipogenesis were decreased by 26±16% measured using ^2H_2O incorporation into palmitate. We expect to observe a similar magnitude (and variability) of response in our study. Our sample size of n=25 will allow us to detect at least a 25% change in rate of DNL (power =0.8, α=0.05).

The entry criteria for the study are specific (volunteers with NAFLD/NASH). Oxford University Hospitals has a large gastroenterology department, with a dedicated NAFLD clinic and considerable experience in running research studies.

The Level of Statistical Significance

It is envisaged that the level of significance to be used will be 5%.

Criteria for the Termination of the Trial

The general effects and safety profiles of both interventions are well characterised and thus it is very unlikely that the trial will be terminated early. Nevertheless, if any new serious safety concerns are raised, the investigators will raise these as outlined via the governance structures outlined in section 10.

Procedure for Accounting for Missing, Unused, and Spurious Data.

Where such data or lack of exists, the investigators will employ standard and accepted statistical methods to normalise data or exclude data where appropriate. Where necessary, this will be discussed with an independent statistician.

Inclusion in Analysis

All randomised participants will be included in the analysis. Analysis of outcome measures will include:

- Imaging analysis: of liver inflammation using multiparametric magnetic resonance techniques
- Physiological analyses: weight, BMI, self-reported health, IPAQ questionnaire, pedometer.
- Biochemical analyses: mainly lipid metabolites and other clinically relevant blood tests as well as hormones and isotopic enrichment of the tracers administered and urine and faecal analysis.
- Gene expression in adipose tissue.
- Genetic analysis, including for PNPLA3 and TM6SF2 gene variants will be performed. These genes are known to be involved in fat metabolism in the liver. Testing does not form part of routine clinical care and will not have any clinical implications for the participant or their family members.

If any participants withdraw from the study, samples and data collected would not usually be destroyed and would be used in the analysis unless requested by the individual.

Procedures for Reporting any Deviation(s) from the Original Statistical Plan

The CI will retain authority on devising and revising, including deviation from the original plans. Where necessary, any deviation(s) will be discussed with an independent statistician. Statistical methods employed will also be described and justified in the final report and academic publications as appropriate.

DATA MANAGEMENT

Source Data

Source documents are where data are first recorded, and from which participants' CRF data are obtained. These include, but are not limited to, hospital records (from which medical history and previous and concurrent medication may be summarised into the CRF), clinical and office charts, laboratory and pharmacy records, diaries, microfiches, radiographs, and correspondence.

CRF entries will be considered source data if the CRF is the site of the original recording (e.g. there is no other written or electronic record of data). All documents will be stored safely in confidential conditions. On all trial-specific documents, other than the signed consent, the participant will be referred to by the trial participant number/code, not by name.

Access to Data

Direct access will be granted to authorised representatives from the Sponsor, host institution and the regulatory authorities to permit trial-related monitoring, audits and inspections.

Data Recording and Record Keeping

All trial data will be entered on to paper CRFs and/or electronic software databases. This will be in accordance with ICH GCP (Section 5.5) which requires that electronic data entry systems are validated and that Standard Operating Procedures are maintained.

The participants will be identified by a unique trial specific number and/or code in any database. The name and any other identifying detail will NOT be included in any trial data electronic file.

Participant identifiers will be securely stored in OCDEM and on encrypted and password protected where necessary. Entry to OCDEM is under swipe access. Personal data will be anonymised before entering onto any laptop computers. The laptop computers used by the study members are also password encrypted. Personal data will be kept on file for 12 months after the study has ended. Anonymised data will be kept indefinitely.

Data from patient participants will be anonymised but identifiable, such that should any information become available as part of the investigations being performed in the study that might be relevant to the health of the individual, they can be appropriately informed. Volunteers are made aware of this in the PIS. No published data will be identifiable to the volunteers.

QUALITY ASSURANCE PROCEDURES

The study will be conducted in accordance with the current approved protocol, ICH GCP, relevant regulations and standard operating procedures. Regular monitoring will be performed by CTRG according to GCP. Data will be evaluated for compliance with the protocol and accuracy in relation to source documents. Following written standard operating procedures, the monitors will verify that the clinical trial is conducted and data are generated, documented and reported in compliance with the protocol, GCP and the applicable regulatory requirements.

Regular monitoring will also be performed in line with the above by our Clinical Research Unit (CRU) at OCDEM according to GCP. SERIOUS BREACHES

The Medicines for Human Use (Clinical Trials) Regulations contain a requirement for the notification of "serious breaches" to the MHRA within 7 days of the Sponsor becoming aware of the breach.

A serious breach is defined as “A breach of GCP or the trial protocol which is likely to affect to a significant degree –

(a) the safety or physical or mental integrity of the subjects of the trial; or

(b) the scientific value of the trial”.

In the event that a serious breach is suspected the Sponsor must be contacted within 1 working day. In collaboration with the C.I., the serious breach will be reviewed by the Sponsor and, if

appropriate, the Sponsor will report it to the REC committee, Regulatory authority and the NHS host organisation within seven calendar days.

ETHICAL AND REGULATORY CONSIDERATIONS

Declaration of Helsinki

The Investigator will ensure that this trial is conducted in accordance with the principles of the Declaration of Helsinki.

Guidelines for Good Clinical Practice

The Investigator will ensure that this trial is conducted in accordance with relevant regulations and with Good Clinical Practice.

Approvals

The protocol, informed consent form, participant information sheet and any proposed advertising material will be submitted to an appropriate Research Ethics Committee (REC), regulatory authorities (MHRA in the UK), and host institution(s) for written approval.

The Investigator will submit and, where necessary, obtain approval from the above parties for all substantial amendments to the original approved documents.

Reporting

The CI shall submit once a year throughout the clinical trial, or on request, an Annual Progress Report to the REC, host organisation and Sponsor. In addition, an End of Trial notification and final report will be submitted to the MHRA, the REC, host organisation and Sponsor.

Participant Confidentiality

The trial staff will ensure that the participants' anonymity is maintained. The participants will be identified only by a participant ID number on all trial documents and any electronic database, with the exception of the CRF, where participant initials may be added. All documents will be stored securely and only accessible by trial staff and authorised personnel. The trial will comply with the Data Protection Act, which requires data to be anonymised as soon as it is practical to do so.

Expenses and Benefits

Both the Liraglutide therapy and cost of the lifestyle intervention will be fully funded for all participants. All volunteers will be reimbursed for reasonable travel costs with presentation of all the relevant receipts (or provided a taxi to and from Study days 1,2 and 3). In addition, to compensate for loss of earnings and the inconvenience caused, participants will be offered £270 for completion of the study (£35 for each study day, £30 for each scanning day, £25 for each intermediate visit). Payment will be made at the end of the individual volunteer's participation in the study.

Other Ethical Considerations

It is possible that significant clinical findings may be found for volunteers. Any significant clinical information that is obtained as a result of this study will be conveyed directly to the volunteers in a confidential face-to-face consultation and appropriate referral to NHS clinical services with the consent of the patient would then be made.

All of the procedures that make up this study have been performed extensively by the research group and are known to be safe. Participants will be asked to avoid excessive exercise and alcohol 24 hours before study day as these factors are known to significantly alter metabolism. Participants should also avoid certain foods which may interfere with sample analysis 24 hours before attending the study days. The foods participants should avoid are those naturally high in 13C and include cornflakes, popcorn, corn, foods rich in corn-starch such as shortbreads and rich in fructose corn syrup such as sweetened breakfast cereals. We would ask participants to fast from 8.00pm the night before the study days. This may interfere with their daily routine but will not have any health impacts.

FINANCE AND INSURANCE

Funding

This trial will be financed through a University of Oxford Novo Nordisk Clinical Fellowship Grant.

Insurance

The University has a specialist insurance policy in place which would operate in the event of any participant suffering harm as a result of their involvement in the research (Newline Underwriting Management Ltd, at Lloyd’s of London). NHS indemnity operates in respect of the clinical treatment that is provided.

PUBLICATION POLICY

Study results will be disseminated via presentation at national and international medical and research conferences and with publications sent to peer review journals.

The Investigators will be involved in reviewing drafts of the manuscripts, abstracts, press releases and any other publications arising from the study. Authors will acknowledge the study funding in resulting publications.

A copy of the final report / publication will be provided to the study participants on request.

Standard Operating Procedures

Liver (Abdominal) MRI/S

Participants would attend Oxford Centre for Clinical Magnetic Resonance Research (OCMR) at the John Radcliffe Hospital, having fasted for >4 hours. The appointment for the OCMR assessment will last approximately 30-60 minutes, during which an abdominal MRI scan and FibroScan will be performed. A full MRI safety questionnaire will be completed and signed by patient prior to scanning.

University of Oxford have developed MRI techniques which allow for the quantitative assessment of relevant liver parameters. ^{1}H MR spectroscopy (^{1}H-MRS) allows for the absolute quantification of fat deposition in the liver (steatosis). We have found excellent correlation between ^{1}H-MRS, T1 mapping and T2* mapping and liver biopsy findings including steatosis in the RIAL study(Banerjee et al. 2014).

MRI–data will be analysed, using software tools available on the scanner console. For each patient, a single Region of Interest (ROI) will be selected on the transverse maps, corresponding to segment 8 of the liver (from where most percutaneous biopsies are taken). MRS data will be analysed offline, using software including AMARES in the jMRUI package and customised software running within MATLAB 2010b.

DXA Scan

Our Clinical Research Unit at OCDEM has access to a dedicated DXA scanner which is extensively used for research purposes to determine body fat and muscle distribution. During the scan, the subject lies on a bed and the scanner passes over the body. The scan is quick, not claustrophobic and uses only very low dose x-rays (approximately 1/4th of a single chest x-ray). All female participants will sign a declaration confirming they are not pregnant. In cases of uncertainty a pregnancy test will be performed.

Our department has standardised reporting techniques to analyse and report scans using standard software tools and these will be utilised to ensure consistency and to allow direct comparison of serial scans for each participant and between participants during the study.

Adipose tissue microdialysis

Adipose tissue microdialysis allows the sampling of interstitial fluid intermediary metabolites including glycerol as a marker of adipose tissue lipolysis. The suppression of glycerol release by insulin provides an assessment of adipose tissue insulin sensitivity

Requirements

CMA microdialysis pump (CMA 106/107), microdialysis vials, microdilaysate solution, CMA syringes, CMA60 microidilaysis catheter, dressing pack and local anaestheic (1% lidocaine, with needle and syringe), betadine solution, tegaderm dressing

A single microdialysis catheter (CMA60, CMA Microdialysis ltd) is inserted under local anesthetic (1ml of 1% lidocaine) into the subcutaneous adipose tissue 5cm to one side of the umbilicus. Using the CMA106 or CMA 107 microdialysis pump, a microdialysate solution (physiological sterile saline solution) will be introduced into the catheter (perfusion rate = 0.3μl/minute). Microdialysis will take place over the duration of the prolonged OGTT. Microdialysate fractions will be analyzed by automated analyzer (ISCUS flex) for glycerol, glucose, lactate and pyruvate. After the prolonged OGTT the catheter will be removed. Samples will be taken and analysed every 30 minutes.

Subcutaneous adipose tissue biopsy

Subcutaneous abdominal adipose tissue biopsies allow both gene expression studies (Polymerase Chain Reaction) and histochemistry to be carried out on samples before and after Liraglutide or Lifestyle interventions

Pro-MagTM Ultra Biopsy 14g (or similar) needles, 20ml syringes and 100ml NaCl, dressing pack and local anaestheic (1% lidocaine, with needle and syringe), betadine solution/chloraprep stick, tegaderm dressing.

The Pro-Mag Ultra needle is inserted into the gluteal or abdominal subcutaneous adipose tissue after skin has been cleaned and local anaesthetic (1ml of 1% lidocaine) has been injected. The needle is placed under the skin and angled horizontally, to maximise tissue capture and reduce bleeding/trauma. Aspiration of adipocytes is then achieved using a syringe, needle and liposuction. Approximately 1 gram of tissue is taken out. One half is placed into formalin and the other into liquid nitrogen. An adhesive dressing will then be applied that can be removed the next day.

Extended OGTT

50ml syringes, electric blanket, Haemacue glucose analyser, 0.9% NaCl, stopwatch, cannulation apparatus, glucose, C^{13}-glucose, ^2H_2O supplemented drinking water

1. The patient will attended the CRU fasted on the study day.
2. Lie subject on bed and place left hand in Hot Box.
3. Choose a straight vein, ideally on one of the upper limbs. Cannulate and take a 5 ml blood sample and place hand back in Hot Box. Attach a 3-way tap with extension tubing. Ensure that blood can be taken easily from this cannula. If required, attach 0.9 % NaCl infusion to the blood sampling cannula and allow it to infuse slowly via a standard giving set and pump to maintain the patency of the cannula.
4. Fasting blood samples will be taken at the start of the OGTT (t=0min)
5. Patient consumes the oral glucose load (75g glucose + 0.5g ^{13}C-glucose)
6. Serum / plasma and breath samples will be collected for analysis at time points including 5, 15, 30, 60, 90, 120, 150 and 180 minutes

b. Magnetic Resonance Spectroscopy (MRS) and LiverMultiScan

Hepatic steatosis (liver fat fraction) was quantified using localised cardiac-triggered proton spectroscopy. Steatosis as a percentage of the liver fat content using ^{1}H MRS was measured with and without water suppression in an 8 cm^3 voxel, avoiding vascular and biliary structures, and in the same region of the liver for each participant at each visit. MRS data was batch analyzed offline, using AMARES as implemented by the OXSA toolbox in MATLAB (The Mathworks, MA, USA) [1]. To characterise extracellular water content, a T1 relaxation time map was acquired using the shortened Modified Look Locker Inversion (shMOLLI) recovery sequence taken in a transverse section of the liver [2]. This sequence sampled the T1 recovery curve using single-shot balanced steady state free precession acquisitions. Iron was quantified using a multiple echo gradient-echo acquisition which allowed calculation of a T2* map of the liver in a single plane. To correct for the effect of iron on T1, the shMOLLI sequence was modelled using a Bloch equation simulation for varying extracellular fluid and iron concentrations, and a correction lookup table was generated. This was then used to remove the effects of elevated iron from the T1 measurements, yielding an 'iron-corrected T1' (cT1) which predicts clinical outcomes and has been used as surrogate marker of inflammation and fibrosis [3, 4]. LiverMultiScan software, LMS Discover (version 4.1.3) was used to calculate hepatic iron content and to determine cT1 values (Perspectum, Oxford, UK).

c. RNA extraction and RNA-sequencing

Total RNA was enriched for polyA-tailed mRNA using oligo (dT) beads. The Illumina TruSeq Stranded mRNA HT Sample Prep Kit was used to prepare cDNA libraries for sequencing. In-house 8bp indexes were used to multiplex samples (10-plex), which were then sequenced over 1 lane of an Illumina HiSeq4000 machine using HiSeq 3000/4000 PE Cluster Kit and SBS Kit. Paired-end sequencing (75bp) was performed at a depth of ~25 million read pairs per sample.
Reads were mapped with STAR 2.5.1b on default settings with GENCODE version 19 as transcriptome and GRCh37 as genome reference. Gene level reads counts for all protein-coding and long intergenic non-coding RNA (lincRNA) transcripts present in GENCODE version 19 were quantified in a strand-specific manner with featureCounts from the Subread package v1.5.0-p2. Significant genes were identified with LIMMA/voom using the duplicateCorrelation function to account for the related nature of the samples.

d. Microbiome and analysis and 16S rRNA Sequencing

Faecal DNA was purified using the Fast DNA Stool Mini Kit (QIAGEN) and quantified using the QuantiFluor dsDNA System (Promega); 12.5 ng of DNA was used for library preparation and sequencing performed with Illumina MiSeq using 600-cycle V3 standard flow cells producing ∼100,000 paired end 2 × 300 base reads with V3-V4 16S-specific sequence primers. Raw reads were denoised, filtered and analysed using DADA2. In brief, Fastq quality was assessed and a trim length of 290 and 280, to remove primers a left trim option was specified. Reads totals were tracked through the pipeline and we retained a final total of 1576447 reads. Diversity analysis was

carried out using the phyloseq package, using a rarefaction depth of 24271. Taxonomy was assigned using Silva 138.1 prokaryotic SSU taxonomic training data formatted for DADA2. Due to the limited availability of matched paired samples, a pooled analysis within treatment arms was undertaken.

The sequencing data and associated analysis have been deposited under project ID PRJEB66353 and are available on GitHub at https://github.com/toryn13/LILIpaper.

2. Supplementary figures

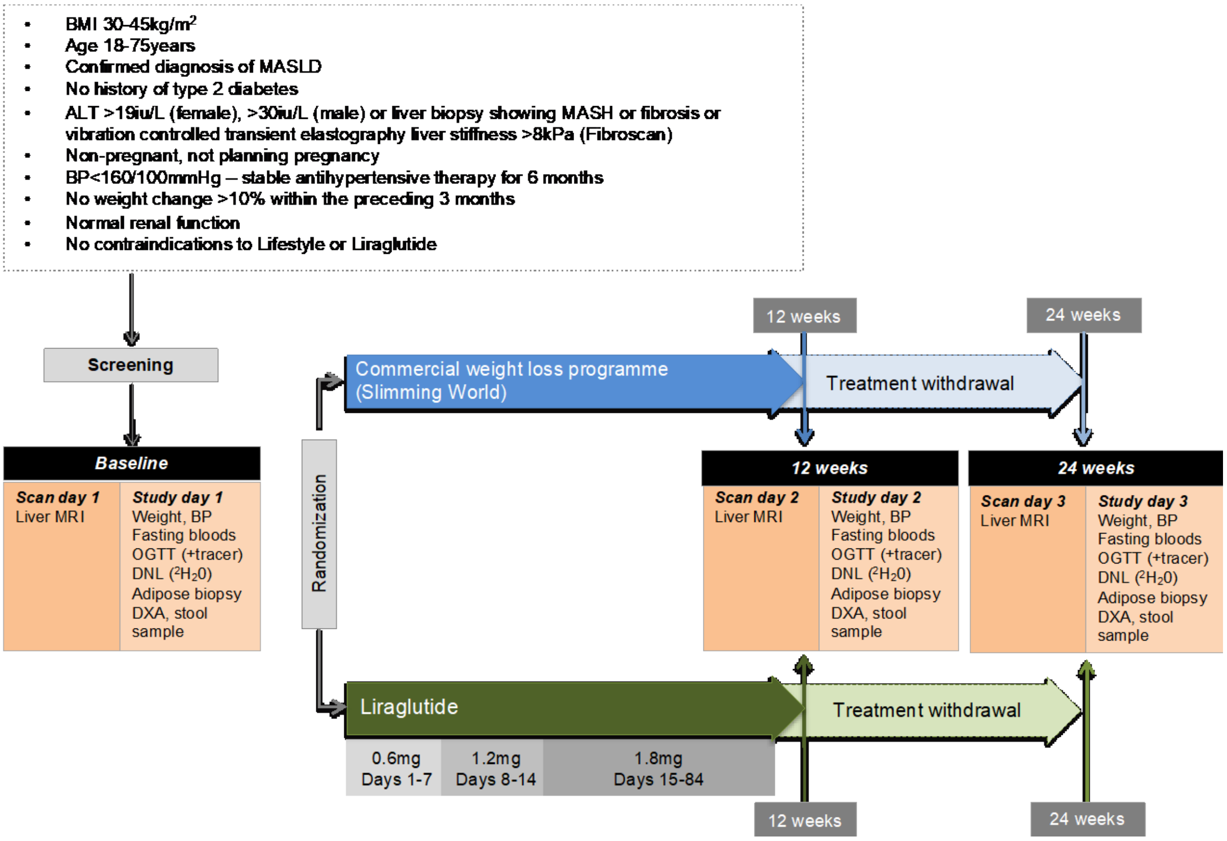

Fig. S1: *Summary of the trial structure, inclusion criteria and investigations comparing 12-weeks of lifestyle intervention with liraglutide, including the assessment of the impact of treatment withdrawal (DNL=de novo lipogenesis, OGTT=oral glucose tolerance test).*

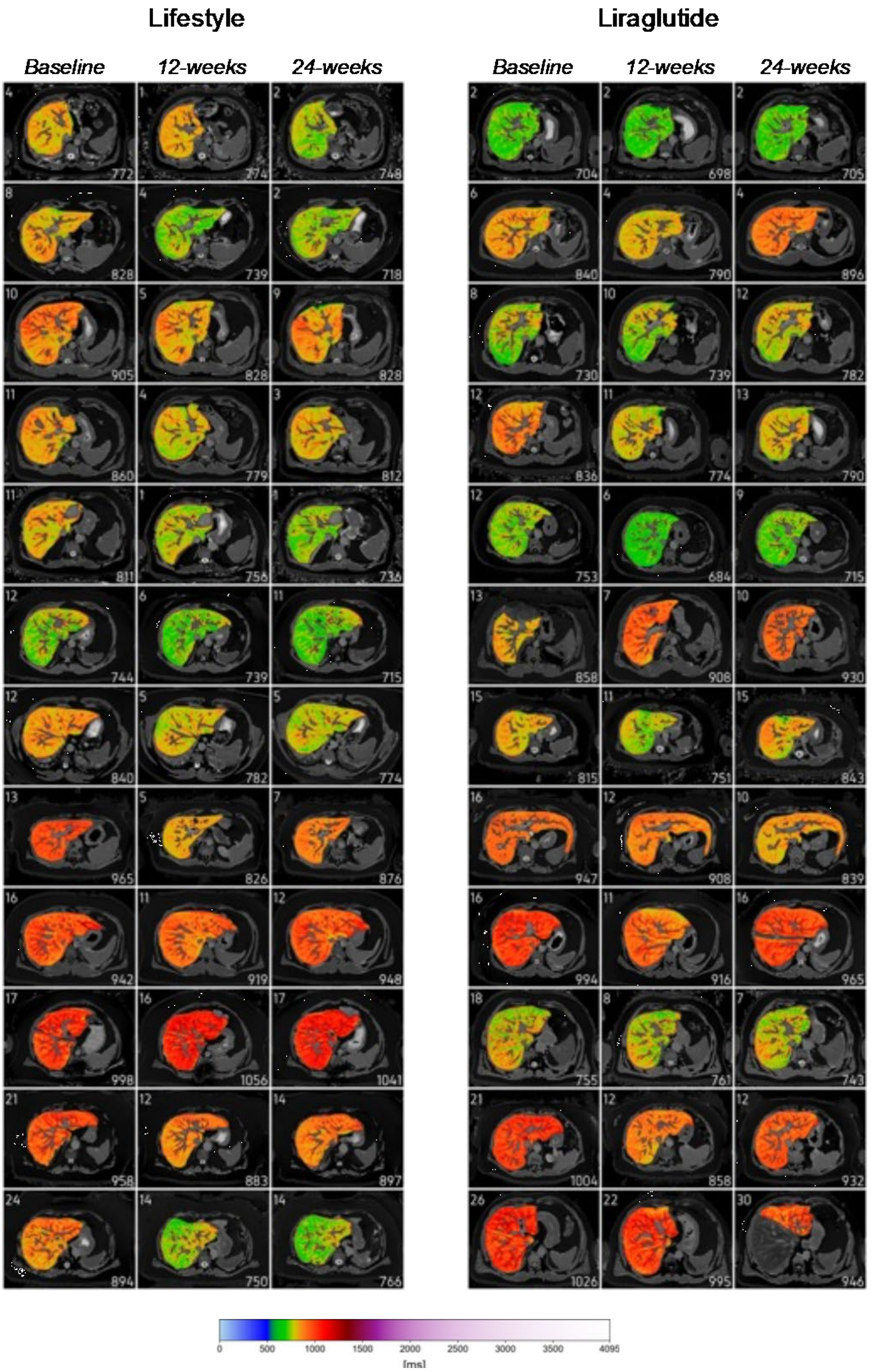

Fig. S2: *LiverMultiscan with colour representation of cT1 values in individual patients with MASLD treated with either lifestyle intervention or liraglutide for 12-weeks and following a further 12-weeks of treatment withdrawal. Bottom right = cT1 value (ms), top left = liver fat content (%).*

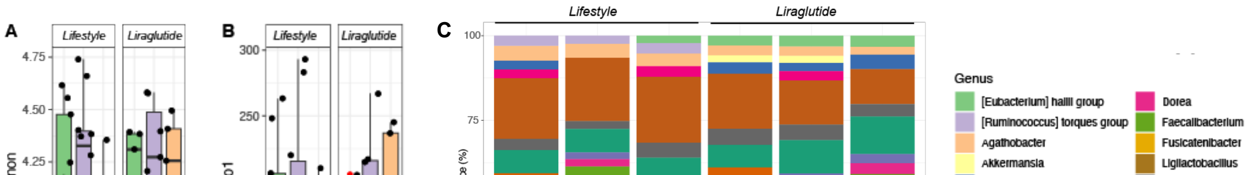

Fig. S3: *Analysis of 16S sequencing of stool microbiome after 12-weeks of lifestyle intervention or GLP1-RA therapy with liraglutide as well as after 12-weeks post-treatment withdrawal; Alpha diversity (A). The relative abundance of each genera was calculated and represented as a taxonomy bar blot (C). N/a represents features that were not classified. Other, represents genera that were below 2.5%. Relative abundance according to phylum is presented in panel D, as well as the ratio of Firmicutes:Bacteroidetes (E).*

3. Supplementary tables

Table S1. *Histology grading of baseline liver biopsies and vibration controlled transient elastography (Fibroscan) (VTCE) where available. Data are presented as median (range) unless otherwise stated. (IQR=interquartile range).*

	Lifestyle	***GLP-1RA, Liraglutide***
Liver biopsy histology		
n	9/14	8/15
Steatosis score	3 (3)	3 (3)
Lobular inflammation score	1 (1)	1 (0-2)
Ballooning score	1 (1)	1 (0-1)
Total NAFLD Activity Score (NAS)	5 (4-5)	5 (4-7)
Fibrosis score	2 (1-3)	2 (0-3)
VTCE		
n	13/14	14/15
Liver stiffness measurement, LSM (kPa)	8.2	9.7
LSM IQR (kPa)	1.6	1.6

Table S2. *Circulating targeted proteome analysis.*

Liraglutide: Baseline vs. end of treatment						
	logFC	AveExpr	t	P.Value	adj.P.Val	B
IL-20RA	-0.169509333	1.102986964	-2.739366594	0.008287306	0.481706335	-2.938152974
SCF	0.185373333	8.777206964	2.650868638	0.010471877	0.481706335	-3.062415534
CDCP1	-0.261226667	3.354912321	-2.490354833	0.015818885	0.485112461	-3.280627649
IL-17C	0.264396667	1.719955179	2.362390199	0.021731902	0.499833742	-3.447602724
IL-24	-0.271650585	1.5095932	-2.068827162	0.043873712	0.743011887	-3.817402835
ADA	-0.142138667	5.300381607	-2.018320147	0.048457297	0.743011887	-3.863167646
CXCL10	-0.266427333	9.811040536	-1.848674825	0.069895957	0.756540461	-4.048743007
IL13	-0.234576	0.641963929	-1.820688243	0.074109314	0.756540461	-4.078058846
MMP-10	-0.199758667	8.158806964	-1.78481494	0.07981999	0.756540461	-4.115085605
FGF-19	0.41162	7.483342143	1.759574812	0.084054521	0.756540461	-4.140763555
CCL11	0.08142	7.583721786	1.67720665	0.099190901	0.756540461	-4.222384259
CD244	-0.141362667	7.01331375	-1.671012294	0.100414357	0.756540461	-4.22838632
CCL4	-0.142332667	5.930994286	-1.639158221	0.106902456	0.756540461	-4.258947677
IL4	-0.138171333	0.71673125	-1.556974183	0.125226519	0.794317836	-4.335422569
CCL19	0.200086	9.252349464	1.527094017	0.132482261	0.794317836	-4.362368643
IL-2RB	-0.159762667	1.346601607	-1.450045179	0.152738552	0.794317836	-4.429707383
FGF-21	-0.293763333	5.832240714	-1.426066615	0.159514284	0.794317836	-4.450027141
MCP-4	0.159007333	15.62372411	1.415758336	0.162498001	0.794317836	-4.458668811
GDNF	-0.089369333	1.70909	-1.410475115	0.164043901	0.794317836	-4.463075955
ST1A1	-0.304168	3.01959125	-1.315741174	0.193732254	0.797873547	-4.539561371
TWEAK	0.073146	9.02044875	1.302175381	0.198296497	0.797873547	-4.550117071
MCP-3	0.121531333	3.714134464	1.299159024	0.199322252	0.797873547	-4.552450516
CSF-1	0.064013333	9.846884821	1.235886869	0.221765797	0.797873547	-4.600249768
CD6	-0.136190667	5.381283571	-1.23353528	0.222634445	0.797873547	-4.601983861
IL-10RB	-0.069615333	5.866142143	-1.220609537	0.227453763	0.797873547	-4.611460918
IL-18R1	-0.082164667	8.568600536	-1.171659207	0.246396744	0.797873547	-4.646510668
STAMBP	-0.192270667	4.095128571	-1.080253239	0.284755068	0.797873547	-4.708367514
AXIN1	-0.235010667	2.240774821	-1.073730462	0.287643121	0.797873547	-4.712601255
CD8A	0.120253333	10.18864161	1.066307026	0.290954583	0.797873547	-4.717390176
IL-1 alpha	-0.062923333	-2.226240714	-1.060781607	0.293436413	0.797873547	-4.720934323
MCP-1	0.075017333	11.66495339	1.053894153	0.296550406	0.797873547	-4.725327774
IFN-gamma	-0.299756667	6.7254475	-1.045172495	0.300526179	0.797873547	-4.730852446
CASP-8	-0.146152	1.554542143	-1.042161409	0.301907223	0.797873547	-4.732749717
SLAMF1	-0.067879333	1.698056607	-1.003752197	0.319904405	0.797873547	-4.756496066
ARTN	-0.084380667	0.329041607	-0.997460824	0.322919696	0.797873547	-4.760305031
IL18	-0.092468	8.354701964	-0.997264441	0.323014122	0.797873547	-4.76042356
CXCL11	-0.146123333	11.28277679	-0.993184068	0.324980271	0.797873547	-4.762881298
uPA	0.060796667	10.31621375	0.963839708	0.339355247	0.797873547	-4.780273761
CCL23	0.087506	9.89340875	0.958657716	0.341936666	0.797873547	-4.783293494
CXCL9	-0.173472667	7.484497143	-0.948762907	0.346901542	0.797873547	-4.789016397
IL10	-0.075328667	3.897508393	-0.924122958	0.359468808	0.806612934	-4.80302087
CCL3	-0.070664	5.064526964	-0.879284832	0.383081656	0.839131247	-4.827598914
IL2	-0.039097333	0.64133	-0.806229024	0.423591551	0.872234218	-4.865122395
OPG	-0.057073333	9.794019107	-0.775771672	0.44121493	0.872234218	-4.879836982
CCL25	0.06922	6.102908929	0.774126847	0.4421788	0.872234218	-4.880616002
SIRT2	-0.191507333	3.335694107	-0.756795435	0.45241003	0.872234218	-4.88872695
EN-RAGE	0.13958	2.97878	0.754747658	0.453627908	0.872234218	-4.889673513
FGF-23	-0.058022667	2.789065357	-0.751512875	0.455555603	0.872234218	-4.89116368
IL7	0.095466667	2.17653	0.736507326	0.464559529	0.872234218	-4.897994908
LIF-R	-0.032504	3.827790179	-0.666953941	0.507596791	0.879136206	-4.927904598
OSM	-0.075643333	2.062627321	-0.633894675	0.528783493	0.879136206	-4.941104832
IL-20	-0.029250667	0.411957321	-0.633308449	0.52916332	0.879136206	-4.941332978
TRANCE	-0.085160667	4.80004375	-0.626109419	0.533839321	0.879136206	-4.944117806
IL8	0.061018667	5.141547679	0.616839034	0.539892172	0.879136206	-4.947657928
CST5	0.049579333	5.107205357	0.612634804	0.5426488	0.879136206	-4.949246344
CXCL5	0.154066667	9.866148214	0.605590684	0.547283586	0.879136206	-4.951883818
Beta-NGF	0.022398667	1.015008036	0.57808589	0.56557133	0.879136206	-4.961895348
DNER	0.031324	8.900324464	0.571833224	0.569770398	0.879136206	-4.964107471
TNFSF14	-0.048379333	3.725592143	-0.559963746	0.577783306	0.879136206	-4.968241654
VEGFA	0.024298667	9.906609643	0.547946088	0.585951364	0.879136206	-4.972340488
4E-BP1	0.099076	6.326274107	0.524457647	0.602072812	0.879136206	-4.980098664
CD40	-0.041241333	11.35902179	-0.518879294	0.605931544	0.879136206	-4.981891941
PD-L1	-0.027882667	6.451255714	-0.511031953	0.611378977	0.879136206	-4.984382608
CXCL6	0.038461333	9.260345357	0.496683388	0.621396584	0.879136206	-4.988839818
CXCL1	0.081329333	8.206815893	0.492798419	0.624121477	0.879136206	-4.990025075
TNFB	-0.038852667	4.841006964	-0.45442226	0.651317661	0.879136206	-5.00123881
HGF	-0.032025333	8.177970714	-0.439541775	0.661995424	0.879136206	-5.005345076
IL-22 RA1	-0.034772667	2.019938393	-0.43224993	0.667253894	0.879136206	-5.007307839
FGF-5	0.02205	0.983330179	0.425248411	0.672318854	0.879136206	-5.009161846
IL5	-0.083785333	1.071607679	-0.420912895	0.675462902	0.879136206	-5.010294853
TNFRSF9	-0.029921333	6.918997143	-0.405227741	0.686885857	0.879136206	-5.014297735
Flt3L	-0.032201333	9.093605893	-0.403676485	0.688019639	0.879136206	-5.014685429
CD5	-0.026148667	5.06091375	-0.354505529	0.724318988	0.90779181	-5.026209391
CCL28	-0.02586	1.570150357	-0.346649844	0.730180369	0.90779181	-5.027912933
IL6	-0.05588	4.111799821	-0.32166737	0.748926978	0.91868376	-5.033078224
NRTN	-0.020764667	0.702349643	-0.269241895	0.788753477	0.9407429	-5.042667854
IL-12B	0.030838667	5.687646964	0.254005323	0.800440937	0.9407429	-5.045137001
IL-15RA	-0.014848667	1.568929643	-0.246610558	0.806129984	0.9407429	-5.046283691
IL-10RA	-0.054034	1.419014821	-0.232006641	0.817396022	0.9407429	-5.048449067
TSLP	0.023732	0.52269875	0.23007434	0.81888964	0.9407429	-5.048725705
LAP TGF-beta	0.01195	7.065839643	0.20761221	0.836300444	0.9407429	-5.051772142
TGF-alpha	-0.008992	3.207044286	-0.20479744	0.838488237	0.9407429	-5.0521319
NT-3	-0.02062	1.723605893	-0.164545515	0.869906423	0.941088536	-5.056740226
LIF	-0.020808667	0.526429286	-0.138245153	0.890552799	0.941088536	-5.059209427
TNF	-0.011786	3.287174107	-0.136112912	0.892230203	0.941088536	-5.059390834
TRAIL	-0.008631333	7.968634464	-0.132948395	0.894720594	0.941088536	-5.059654871
CCL20	-0.034618	7.100073036	-0.132881218	0.894773473	0.941088536	-5.059660409
CX3CL1	-0.007574667	5.744388214	-0.116188393	0.907927463	0.941088536	-5.060949803
MMP-1	-0.021972	6.99132625	-0.113053388	0.910400866	0.941088536	-5.061172695
IL-17A	-0.009621333	1.857581786	-0.085011268	0.932561877	0.950664281	-5.062895412
MCP-2	-0.006444667	8.441094286	-0.075197638	0.940330974	0.950664281	-5.063383111
IL33	5.00E-05	0.8376325	0.000810527	0.999356233	0.999356233	-5.065137126

Liraglutide: End of treatment vs. 12-weeks post-treatment						
	logFC	AveExpr	t	P.Value	adj.P.Val	B
MMP-10	0.570406562	8.221773878	4.571489572	3.43E-05	0.003159625	2.117430985
IL-10RB	0.21339469	5.888918776	3.93805026	0.000266257	0.012247823	0.185082628
Flt3L	0.278625609	9.137382653	3.530568297	0.000929584	0.023311929	-0.982262762
FGF-23	0.327806419	2.839308776	3.501604272	0.001013562	0.023311929	-1.062542576
IL13	0.536070789	0.720214082	2.68407323	0.009964929	0.183354687	-3.150840945
CX3CL1	0.183103842	5.796115714	2.55734929	0.013778006	0.211262763	-3.439686185
CASP-8	0.255264248	1.525351633	2.294741041	0.026184314	0.309856453	-4.004074866
IL-18R1	0.167023118	8.550617755	2.282638691	0.026944039	0.309856453	-4.028926447
CDCP1	0.234805909	3.264922653	2.229305179	0.030530868	0.312093318	-4.137188506
OPG	0.143947298	9.826165918	2.091328797	0.041840079	0.374459261	-4.407587086
LIF-R	0.08905486	3.853683061	2.060963225	0.044772303	0.374459261	-4.465182897
CXCL1	-0.334872375	8.227875306	-1.753102498	0.085998826	0.602276485	-5.008521016
TGF-alpha	0.069909188	3.237135918	1.72124172	0.091677189	0.602276485	-5.060404235
OSM	0.29772898	2.089782449	1.701146088	0.09541608	0.602276485	-5.092697699
IL-17A	0.168837387	1.844296122	1.678527497	0.099774143	0.602276485	-5.128644578
TNFRSF9	0.127206835	6.928335918	1.652325064	0.105026107	0.602276485	-5.169753616
IL-24	0.186517576	1.495012667	1.625239696	0.11129022	0.602276485	-5.206641736
IL-1 alpha	0.120753816	-2.24214449	1.578437391	0.121063896	0.618771025	-5.28256522
IL-22 RA1	-0.14603187	1.972944286	-1.539420397	0.130299089	0.630921903	-5.340265601
DNER	0.077882178	8.930449796	1.472616414	0.14741202	0.678095294	-5.436020959
CXCL5	-0.448245064	9.840097755	-1.419286022	0.162307211	0.681395087	-5.509682326
IFN-gamma	0.308138746	6.591345714	1.417098653	0.162942303	0.681395087	-5.512650461
ADA	0.104793491	5.263135918	1.379427312	0.174185727	0.696742907	-5.563107846
NRTN	-0.103165592	0.636322653	-1.308557462	0.196944214	0.729687178	-5.654626005
CD6	0.141113633	5.380725102	1.282889006	0.205719627	0.729687178	-5.686667565
ARTN	0.10619202	0.306957755	1.24094161	0.220686089	0.729687178	-5.737754761
IL-20	-0.04889897	0.38294	-1.216527046	0.229759991	0.729687178	-5.766756949
CD5	0.089099716	5.063001633	1.208958462	0.232627785	0.729687178	-5.775637919
LIF	-0.174461681	0.48864898	-1.1863979	0.241331401	0.729687178	-5.801801299
IL10	0.109186719	3.928242449	1.184027797	0.242259316	0.729687178	-5.804522978
VEGFA	0.059859641	9.917119592	1.174860247	0.245872853	0.729687178	-5.815002147
4E-BP1	-0.17048324	6.290417551	-1.116431803	0.269820724	0.757948021	-5.879981357
MCP-1	0.076654474	11.7322298	1.093799468	0.279527025	0.757948021	-5.904307286
IL4	0.101057401	0.697237347	1.047492537	0.300142802	0.757948021	-5.952602178
CST5	0.088823723	5.13254	1.045379796	0.30110773	0.757948021	-5.954758127
CD8A	-0.119387083	10.21625796	-1.037695513	0.304635246	0.757948021	-5.962564528
LAP TGF-bet	0.059593431	7.096401429	1.037279721	0.304826921	0.757948021	-5.96298536
IL-17C	-0.143668103	1.737489592	-0.990529936	0.326905295	0.785101141	-6.009272603
AXIN1	-0.25899701	2.15452551	-0.960855864	0.341462367	0.785101141	-6.037591099
SLAMF1	-0.067015128	1.623068367	-0.944786033	0.349521717	0.785101141	-6.052581257
CCL28	0.067419612	1.58199551	0.944073299	0.34988203	0.785101141	-6.053240471
IL5	-0.177091878	0.912567551	-0.907809743	0.368535053	0.789109778	-6.086148067
HGF	0.068853057	8.164302857	0.907259249	0.368823049	0.789109778	-6.086638037
TNFSF14	0.087893546	3.670327347	0.86335269	0.392257009	0.811192781	-6.124791711
CXCL10	0.110683	9.763319184	0.855071716	0.396779078	0.811192781	-6.131782338
FGF-19	0.181374464	7.733140612	0.823714681	0.414194681	0.816415194	-6.157660951
NT-3	-0.130151858	1.662924286	-0.818595279	0.417081675	0.816415194	-6.161796799
PD-L1	0.043503988	6.465767143	0.764565393	0.448290283	0.839098696	-6.20391601
IL-15RA	0.042628525	1.56463	0.758689934	0.451764727	0.839098696	-6.208327337
CCL19	-0.083683391	9.220363061	-0.739919465	0.462969296	0.839098696	-6.222197728
FGF-21	-0.164484112	5.744727143	-0.725521504	0.471671093	0.839098696	-6.232607006
CCL20	-0.144215903	6.914081633	-0.712519666	0.479608415	0.839098696	-6.241835065
CCL4	0.054891844	5.908803469	0.704614334	0.48447096	0.839098696	-6.247366054
CSF-1	0.031277055	9.850923469	0.635259378	0.528290156	0.839098696	-6.293295635
SIRT2	-0.188926574	3.255158163	-0.634890882	0.528528422	0.839098696	-6.293527198
IL18	0.060558905	8.297866735	0.631131822	0.530962221	0.839098696	-6.295881846
CXCL11	0.080637119	11.2664	0.613445859	0.542491184	0.839098696	-6.306775647
IL33	0.04325648	0.856199592	0.59387701	0.555395918	0.839098696	-6.318474085
MCP-3	-0.063654045	3.728226531	-0.588543548	0.55893973	0.839098696	-6.321597707
SCF	-0.040046726	8.815671837	-0.575578413	0.567601361	0.839098696	-6.329075122
TNF	0.040537912	3.250492653	0.552117444	0.583441504	0.839098696	-6.342188233
FGF-5	0.030968513	1.001554082	0.533560468	0.596119316	0.839098696	-6.352178824
uPA	0.032820604	10.33436694	0.531480208	0.597548553	0.839098696	-6.353277754
IL-12B	0.060824095	5.679049388	0.529203349	0.599114701	0.839098696	-6.354475679
TRAIL	0.035043944	7.978062245	0.528995501	0.599257766	0.839098696	-6.354584781
CXCL9	0.072981093	7.433492041	0.509355315	0.612847802	0.839098696	-6.364703017
CXCL6	-0.038499714	9.32710551	-0.499951983	0.6194038	0.839098696	-6.369413424
MCP-4	-0.052628227	15.67139531	-0.496304369	0.621955384	0.839098696	-6.371217247
IL6	-0.077677004	3.987925714	-0.485807869	0.629324022	0.839098696	-6.376335056
CCL11	0.020830661	7.632733469	0.430030701	0.669106769	0.86665822	-6.401711909
EN-RAGE	-0.059288821	3.019187143	-0.429938472	0.669173392	0.86665822	-6.401751332
IL-20RA	0.029409459	1.078230816	0.417401857	0.678254259	0.86665822	-6.407031976
ST1A1	0.113869239	2.928964286	0.384857914	0.702050605	0.867000808	-6.42001584
STAMBP	-0.075135127	4.020209592	-0.381171945	0.70476546	0.867000808	-6.421420432
TNFB	0.031079053	4.854867551	0.351392487	0.726838869	0.867000808	-6.432275425
IL8	-0.034598224	5.177640408	-0.347465206	0.729767909	0.867000808	-6.433641459
GDNF	0.024461751	1.667241837	0.341115368	0.734512337	0.867000808	-6.435817822
IL7	0.05934568	2.285793878	0.325329267	0.74635232	0.867000808	-6.441055295
CCL25	0.030169901	6.151912857	0.318659544	0.751373602	0.867000808	-6.443193934
MCP-2	0.026248678	8.474095714	0.315291006	0.753913746	0.867000808	-6.444257289
IL2	0.012714619	0.621940816	0.245968207	0.806760471	0.916320535	-6.463639856
TRANCE	0.031183641	4.752934898	0.218593315	0.827897923	0.928861084	-6.469978654
CD244	0.014358035	7.003618776	0.193477198	0.847405457	0.939292796	-6.475138568
CCL3	0.011431979	5.030945306	0.160341163	0.873288343	0.946038838	-6.480985146
CCL23	-0.014306351	9.956077347	-0.156597187	0.876222172	0.946038838	-6.481576964
Beta-NGF	-0.006179269	1.025756939	-0.146248366	0.884340653	0.946038838	-6.483140159
TWEAK	-0.007191318	9.070287755	-0.126075603	0.900201135	0.951936833	-6.485880421
CD40	0.009286246	11.37379959	0.095526691	0.924296037	0.966309494	-6.489257708
IL-2RB	-0.007822083	1.330170408	-0.070604581	0.944007173	0.974309104	-6.491323492
TSLP	0.006064878	0.442172653	0.059088001	0.953128471	0.974309104	-6.492068761
IL-10RA	-0.009773587	1.290724286	-0.040424777	0.967922933	0.978559449	-6.492995497
MMP-1	-0.001102579	7.034041224	-0.005752229	0.995434362	0.995434362	-6.493794444

Lifestyle: Baseline vs. end of treatment						
	logFC	***AveExpr***	***t***	***P.Value***	***adj.P.Val***	***B***
TWEAK	0.147049231	9.02044875	2.437068309	0.018077811	0.682822462	-3.40860158
CDCP1	-0.258733077	3.354912321	-2.296263498	0.025504655	0.682822462	-3.578205316
IL-17C	-0.274429231	1.719955179	-2.282716992	0.02634584	0.682822462	-3.594128872
CXCL6	0.178072308	9.260345357	2.140808068	0.036748664	0.682822462	-3.756625524
TRAIL	0.148998462	7.968634464	2.136549903	0.037109916	0.682822462	-3.761377339
IL-1 alpha	-0.106013077	-2.226240714	-1.663794736	0.101855467	0.970187267	-4.240687617
AXIN1	0.386345385	2.240774821	1.643272074	0.106045808	0.970187267	-4.259214928
IL-17A	-0.192416923	1.857581786	-1.58274352	0.119227758	0.970187267	-4.312694751
CCL20	-0.441536923	7.100073036	-1.577809984	0.120357964	0.970187267	-4.31697658
IL-24	-0.22724885	1.5095932	-1.569780487	0.122919368	0.970187267	-4.326788084
CCL11	0.081276923	7.583721786	1.558650626	0.124829048	0.970187267	-4.333493927
SIRT2	0.418587692	3.335694107	1.53994637	0.129321339	0.970187267	-4.349447846
MCP-1	0.108671539	11.66495339	1.421272113	0.160896707	0.970187267	-4.446682891
TSLP	-0.150600769	0.52269875	-1.359211627	0.179640268	0.970187267	-4.494744246
FGF-19	0.321515385	7.483342143	1.279496416	0.206106601	0.970187267	-4.553614352
IL2	-0.064135385	0.64133	-1.231218243	0.223492775	0.970187267	-4.587681392
MCP-4	0.145590769	15.62372411	1.206790438	0.232690199	0.970187267	-4.604457928
Beta-NGF	0.04977	1.015008036	1.195814706	0.236911412	0.970187267	-4.611894498
ST1A1	0.253023846	3.01959125	1.01893022	0.312708088	0.970187267	-4.722961426
STAMBP	0.193655385	4.095128571	1.012903726	0.315552197	0.970187267	-4.726450914
NRTN	-0.083363846	0.702349643	-1.006286078	0.318695344	0.970187267	-4.730260098
CASP-8	-0.144955385	1.554542143	-0.962255991	0.340142812	0.970187267	-4.755000194
VEGFA	-0.045669231	9.906609643	-0.958749457	0.341890853	0.970187267	-4.756925205
IL6	-0.177699231	4.111799821	-0.952274611	0.345134118	0.970187267	-4.760462151
CXCL1	0.168379231	8.206815893	0.949809833	0.346374011	0.970187267	-4.761802553
IL10	-0.083043077	3.897508393	-0.948416188	0.347076365	0.970187267	-4.762558983
PD-L1	0.05534	6.451255714	0.944232512	0.349190398	0.970187267	-4.764823395
NT-3	-0.125742308	1.723605893	-0.934124712	0.354332514	0.970187267	-4.770254796
ARTN	-0.083692308	0.329041607	-0.921010295	0.361077042	0.970187267	-4.777218538
IL8	0.095376154	5.141547679	0.89758376	0.37332934	0.970187267	-4.789423455
CD244	0.077837692	7.01331375	0.856566163	0.395410378	0.970187267	-4.810066151
Flt3L	-0.073354615	9.093605893	-0.85607751	0.395678234	0.970187267	-4.810306478
TNFSF14	-0.071763846	3.725592143	-0.773271108	0.442680754	0.970187267	-4.849118953
CXCL11	0.12209	11.28277679	0.772531709	0.443114736	0.970187267	-4.849448325
IL-18R1	-0.057756923	8.568600536	-0.766736799	0.446524632	0.970187267	-4.852019139
MCP-3	-0.075635385	3.714134464	-0.75270555	0.454844305	0.970187267	-4.858166081
IL-10RB	-0.045362308	5.866142143	-0.740445398	0.462186729	0.970187267	-4.863446881
IFN-gamma	-0.226814615	6.7254475	-0.736234567	0.464724132	0.970187267	-4.865241167
MMP-1	0.153091539	6.99132625	0.733316107	0.466487428	0.970187267	-4.866478922
FGF-23	0.057396923	2.789065357	0.692075378	0.49180854	0.970187267	-4.883457826
IL-10RA	-0.170032308	1.419014821	-0.679658565	0.499578211	0.970187267	-4.88838223
EN-RAGE	-0.132532308	2.97878	-0.66715445	0.507469702	0.970187267	-4.893253291
IL-12B	-0.085257692	5.687646964	-0.653742689	0.516008218	0.970187267	-4.898379683
CD6	0.076909231	5.381283571	0.648498153	0.519367831	0.970187267	-4.90035662
IL13	0.085836923	0.641963929	0.620229187	0.537674574	0.970187267	-4.910744145
CCL25	0.058484615	6.102908929	0.608903148	0.545101595	0.970187267	-4.914778645
IL-22 RA1	-0.051818462	2.019938393	-0.599663335	0.551199108	0.970187267	-4.918015978
HGF	-0.046104615	8.177970714	-0.589083622	0.558222912	0.970187267	-4.921663099
IL-20	-0.028637692	0.411957321	-0.577222935	0.56614995	0.970187267	-4.925675993
OSM	-0.072193846	2.062627321	-0.563213001	0.575584372	0.970187267	-4.930312697
TRANCE	0.08166	4.80004375	0.558916083	0.578493178	0.970187267	-4.931712345
TNFB	0.051112308	4.841006964	0.556532205	0.580110005	0.970187267	-4.932484305
CD40	0.046330769	11.35902179	0.542661737	0.589560334	0.970187267	-4.936911485
CD8A	0.065633077	10.18864161	0.54179371	0.590154163	0.970187267	-4.937184884
SCF	0.040405385	8.777206964	0.537905815	0.592817387	0.970187267	-4.938404151
TNFRSF9	0.042275385	6.918997143	0.533005684	0.59618204	0.970187267	-4.93992854
SLAMF1	0.037238462	1.698056607	0.512633186	0.61026563	0.970187267	-4.94611881
LIF	-0.073999231	0.526429286	-0.457676816	0.648991958	0.970187267	-4.961629513
CXCL10	0.068673846	9.811040536	0.443607793	0.659070638	0.970187267	-4.965320987
MCP-2	0.04025	8.441094286	0.437215624	0.663671069	0.970187267	-4.966960497
MMP-10	0.051594615	8.158806964	0.429158766	0.669488166	0.970187267	-4.968993412
CXCL5	0.114447692	9.866148214	0.418797023	0.676999418	0.970187267	-4.971552837
CCL23	0.038638462	9.89340875	0.394068402	0.695057975	0.970187267	-4.977410409
OPG	-0.029896154	9.794019107	-0.378304969	0.706663837	0.970187267	-4.98095984
IL-20RA	0.024794615	1.102986964	0.373026738	0.710565825	0.970187267	-4.982116196
4E-BP1	-0.074740769	6.326274107	-0.368320225	0.714051755	0.970187267	-4.983133695
CCL3	-0.029731538	5.064526964	-0.344409239	0.731855148	0.970187267	-4.988104781
IL7	-0.04662	2.17653	-0.334829434	0.739030459	0.970187267	-4.990003412
TNF	0.030767692	3.287174107	0.330791132	0.742062216	0.970187267	-4.990787815
CX3CL1	0.023086154	5.744388214	0.329668044	0.742906108	0.970187267	-4.991004282
IL5	-0.068834615	1.071607679	-0.321926824	0.748731478	0.970187267	-4.992476433
FGF-21	-0.064945385	5.832240714	-0.293505717	0.770242979	0.984199363	-4.997582819
CCL4	-0.025445385	5.930994286	-0.272804391	0.786027731	0.990610291	-5.001006625
DNER	-0.014025385	8.900324464	-0.238359791	0.812490037	0.996195584	-5.006150656
IL-15RA	0.014276154	1.568929643	0.220730056	0.826121958	0.996195584	-5.008516087
TGF-alpha	0.010032308	3.207044286	0.21271353	0.832338744	0.996195584	-5.009531753
ADA	0.011522308	5.300381607	0.152315231	0.879497044	0.996195584	-5.015978308
uPA	0.009461538	10.31621375	0.139640968	0.889455004	0.996195584	-5.017060642
CST5	-0.010116154	5.107205357	-0.116370381	0.907783909	0.996195584	-5.01880342
LIF-R	-0.005782308	3.827790179	-0.110455229	0.912451395	0.996195584	-5.019195952
IL4	0.010218462	0.71673125	0.107195122	0.915025194	0.996195584	-5.019403548
CCL28	0.006793846	1.570150357	0.084782114	0.932743217	0.996195584	-5.020662492
CXCL9	0.012403846	7.484497143	0.063155192	0.949872446	0.996195584	-5.021598698
IL18	0.004906923	8.354701964	0.049266779	0.960885603	0.996195584	-5.022055606
CD5	-0.003723077	5.06091375	-0.046989575	0.962692156	0.996195584	-5.022119752
GDNF	0.002712308	1.70909	0.039851247	0.968356376	0.996195584	-5.022301162
IL33	-0.001756154	0.8376325	-0.02650244	0.978952747	0.996195584	-5.022560375
IL-2RB	-0.002336154	1.346601607	-0.01973939	0.984322878	0.996195584	-5.022651898
FGF-5	0.000966154	0.983330179	0.017346283	0.986223287	0.996195584	-5.022677872
CSF-1	-0.00057	9.846884821	-0.010244934	0.991863029	0.996195584	-5.022735213
CCL19	-0.000947692	9.252349464	-0.006733525	0.994651888	0.996195584	-5.022752661
LAP TGF-beta	0.000296154	7.065839643	0.004789922	0.996195584	0.996195584	-5.022759216

Lifestyle: End of treatment vs. 12-weeks post-treatment						
	logFC	*AveExpr*	*t*	*P.Value*	*adj.P.Val*	*B*
IL10	0.25840662	3.928242449	2.666115792	0.010438755	0.811917911	-3.131190611
SLAMF1	-0.173321491	1.623068367	-2.324857413	0.024376034	0.811917911	-3.551522365
EN-RAGE	0.31887732	3.019187143	2.200089834	0.032669935	0.811917911	-3.695186215
LIF-R	0.098387729	3.853683061	2.166391003	0.035300779	0.811917911	-3.732996902
IL-17C	0.279999146	1.737489592	1.836738149	0.072473666	0.997328877	-4.079080766
IL7	0.314601023	2.285793878	1.640882349	0.107389641	0.997328877	-4.262782425
CCL25	0.15229249	6.151912857	1.530434792	0.13250394	0.997328877	-4.358644788
TNF	-0.108313344	3.250492653	-1.403574101	0.166912108	0.997328877	-4.461572576
IL-24	0.160399201	1.495012667	1.270716074	0.210541051	0.997328877	-4.552463282
FGF-23	-0.125124855	2.839308776	-1.271676035	0.209643799	0.997328877	-4.560173583
TSLP	-0.135416865	0.442172653	-1.255258793	0.215489769	0.997328877	-4.571832218
ST1A1	-0.384292568	2.928964286	-1.23577464	0.222583994	0.997328877	-4.585490036
GDNF	-0.092482331	1.667241837	-1.227031767	0.225822786	0.997328877	-4.591555249
IFN-gamma	-0.254749688	6.591345714	-1.114681325	0.270562833	0.997328877	-4.665971489
LAP TGF-beta	0.066933248	7.096401429	1.108466781	0.273209121	0.997328877	-4.669895105
TGF-alpha	0.046983914	3.237135918	1.100626822	0.276573522	0.997328877	-4.674815886
VEGFA	0.058341225	9.917119592	1.08945912	0.281416115	0.997328877	-4.681769263
FGF-21	0.253270545	5.744727143	1.062904662	0.293168167	0.997328877	-4.69803753
TRAIL	-0.073585783	7.978062245	-1.056856957	0.295891518	0.997328877	-4.701690192
CCL19	-0.118234345	9.220363061	-0.994654203	0.324915463	0.997328877	-4.738124222
DNER	0.054000582	8.930449796	0.971478693	0.336202697	0.997328877	-4.751166887
CD40	0.089026247	11.37379959	0.871336534	0.387927856	0.997328877	-4.804168473
IL-15RA	-0.048637036	1.56463	-0.823596336	0.414261282	0.997328877	-4.827498757
TWEAK	-0.046381691	9.070287755	-0.773664091	0.442940793	0.997328877	-4.850548558
SIRT2	-0.239425525	3.255158163	-0.765525707	0.447723895	0.997328877	-4.854173751
4E-BP1	-0.122443642	6.290417551	-0.76290426	0.449271001	0.997328877	-4.855333578
CCL28	-0.055222921	1.58199551	-0.735736214	0.465487999	0.997328877	-4.867127246
CXCL6	0.058981384	9.32710551	0.728734006	0.469721504	0.997328877	-4.870099837
CST5	0.06323525	5.13254	0.708088697	0.482330502	0.997328877	-4.878703829
IL6	-0.116099442	3.987925714	-0.690852768	0.493001043	0.997328877	-4.885703126
CD244	0.053831392	7.003618776	0.69016619	0.493428781	0.997328877	-4.885978466
CSF-1	-0.035565044	9.850923469	-0.687277079	0.495230942	0.997328877	-4.887134181
IL-20	-0.028800389	0.38294	-0.681716324	0.498709799	0.997328877	-4.88934535
CCL3	-0.051077901	5.030945306	-0.681616249	0.498772529	0.997328877	-4.889384984
OPG	0.04658017	9.826165918	0.643877365	0.522733978	0.997328877	-4.903927021
TNFSF14	-0.068805606	3.670327347	-0.64304057	0.523272115	0.997328877	-4.904240318
CXCL1	0.125490136	8.227875306	0.625058787	0.534906556	0.997328877	-4.910876617
MCP-3	0.070385158	3.728226531	0.619180078	0.538739151	0.997328877	-4.91300632
MMP-1	0.123761712	7.034041224	0.614321879	0.541917115	0.997328877	-4.914751475
NRTN	-0.048799299	0.636322653	-0.588917958	0.558690585	0.997328877	-4.923657969
CD8A	0.066139041	10.21625796	0.546957751	0.58695349	0.997328877	-4.937561505
CXCL10	-0.072386825	9.763319184	-0.532064692	0.597146822	0.997328877	-4.942253755
CASP-8	-0.061765745	1.525351633	-0.528292766	0.599741586	0.997328877	-4.94342194
MCP-2	0.044119832	8.474095714	0.504221417	0.616423238	0.997328877	-4.950684306
TNFRSF9	-0.040285759	6.928335918	-0.497874503	0.620856464	0.997328877	-4.952543627
AXIN1	-0.13894239	2.15452551	-0.490435105	0.626070907	0.997328877	-4.954693446
IL-10RB	0.027503331	5.888918776	0.48291004	0.631365092	0.997328877	-4.95683556
CCL23	0.046326159	9.956077347	0.48246374	0.631679699	0.997328877	-4.956961579
CXCL5	0.15520007	9.840097755	0.46755167	0.64223084	0.997328877	-4.961106136
MMP-10	-0.060214882	8.221773878	-0.459156088	0.648204322	0.997328877	-4.963383038
IL-17A	0.047237163	1.844296122	0.446814163	0.657028122	0.997328877	-4.966656219
IL-20RA	0.032170845	1.078230816	0.434423289	0.665936821	0.997328877	-4.969853718
Flt3L	0.035908074	9.137382653	0.432911363	0.667027224	0.997328877	-4.970237789
MCP-4	-0.047227418	15.67139531	-0.423747215	0.673651883	0.997328877	-4.972537394
IL-18R1	-0.030193434	8.550617755	-0.392605512	0.696357013	0.997328877	-4.979987941
IL-1 alpha	-0.030353791	-2.24214449	-0.37750501	0.707470152	0.997328877	-4.983397918
OSM	-0.067891133	2.089782449	-0.369076885	0.71370105	0.997328877	-4.98524349
IL18	-0.035299064	8.297866735	-0.35001652	0.727864625	0.997328877	-4.989264805
PD-L1	0.020538042	6.465767143	0.343421825	0.732787794	0.997328877	-4.990606866
ARTN	0.030865156	0.306957755	0.343171517	0.732974882	0.997328877	-4.990657305
FGF-5	0.020635008	1.001554082	0.338260448	0.73664888	0.997328877	-4.991639554
STAMBP	-0.066837251	4.020209592	-0.322611449	0.748397094	0.997328877	-4.994675647
IL-12B	-0.038426685	5.679049388	-0.318099613	0.751795642	0.997328877	-4.995524461
CXCL11	-0.043874226	11.2664	-0.317565999	0.752197918	0.997328877	-4.995624064
uPA	0.019120313	10.33436694	0.294590551	0.769582904	0.997328877	-4.999754891
CX3CL1	0.020986328	5.796115714	0.278876777	0.781542956	0.997328877	-5.002402469
FGF-19	-0.060720736	7.733140612	-0.262374112	0.794161037	0.997328877	-5.005027462
CD6	0.028456142	5.380725102	0.246138424	0.806629475	0.997328877	-5.007454401
SCF	0.016856423	8.815671837	0.2305081	0.818681148	0.997328877	-5.009644947
IL-2RB	-0.026285525	1.330170408	-0.225740969	0.822365776	0.997328877	-5.010284548
MCP-1	0.016410703	11.7322298	0.222797692	0.82464273	0.997328877	-5.010672791
CCL11	-0.009704098	7.632733469	-0.190605262	0.849642475	0.997328877	-5.014587456
IL13	0.034498528	0.720214082	0.16434484	0.870152998	0.997328877	-5.017330183
CDCP1	0.016047845	3.264922653	0.144964121	0.885349016	0.997328877	-5.019094493
IL33	0.010353656	0.856199592	0.135245354	0.89298608	0.997328877	-5.019896103
NT-3	0.022228084	1.662924286	0.133016093	0.894739318	0.997328877	-5.020072142
HGF	0.009257763	8.164302857	0.116063991	0.9080883	0.997328877	-5.021315179
IL-10RA	-0.024635662	1.290724286	-0.096948522	0.923172823	0.997328877	-5.02251403
IL-22 RA1	0.008705082	1.972944286	0.087310358	0.930789734	0.997328877	-5.023036951
ADA	-0.005916537	5.263135918	-0.074099517	0.941240573	0.997328877	-5.023664863
CD5	0.005558905	5.063001633	0.071764161	0.943089168	0.997328877	-5.023765175
CCL20	-0.013710299	6.914081633	-0.064448663	0.948881886	0.997328877	-5.024058619
CCL4	-0.003555043	5.908803469	-0.043418202	0.965549121	0.997328877	-5.024726687
IL4	-0.00431218	0.697237347	-0.042526828	0.966255957	0.997328877	-5.024749249
TNFB	-0.003760865	4.854867551	-0.040457196	0.967897223	0.997328877	-5.02479983
IL2	0.001926813	0.621940816	0.035464882	0.971856809	0.997328877	-5.024911459
LIF	0.003490426	0.48864898	0.022583543	0.982076527	0.997328877	-5.025131682
IL5	-0.004300078	0.912567551	-0.020972769	0.983354723	0.997328877	-5.025152345
CXCL9	-0.002767738	7.433492041	-0.018378865	0.985413151	0.997328877	-5.025182408
IL8	-0.00168357	5.177640408	-0.016086889	0.987232064	0.997328877	-5.025205674
Beta-NGF	-0.000367007	1.025756939	-0.008264403	0.993440448	0.997328877	-5.025261777
TRANCE	0.000504584	4.752934898	0.003365325	0.997328877	0.997328877	-5.025278558

Baseline: Lifestyle vs. Liraglutide						
	**logFC**	**AveExpr**	**t**	**P.Value**	**adj.P.Val**	**B**
**CXCL6**	0.410806667	9.260345357	2.726166038	0.008584112	0.654372817	-3.778015913
**IL4**	0.437304103	0.71673125	2.532249741	0.014225496	0.654372817	-3.901489984
**CD8A**	0.456313897	10.18864161	2.079257917	0.042280858	0.93605312	-4.165885175
**IL-18R1**	-0.278366205	8.568600536	-2.03982186	0.046194335	0.93605312	-4.187120144
**IL-10RA**	-0.869111385	1.419014821	-1.917645202	0.060367414	0.93605312	-4.250927072
**CD244**	0.297888513	7.01331375	1.809498357	0.075852645	0.93605312	-4.304817275
**IL5**	0.683957487	1.071607679	1.765683496	0.083012893	0.93605312	-4.325934093
**SCF**	-0.221036821	8.777206964	-1.624295696	0.110044406	0.93605312	-4.391160438
**TNFB**	0.266189744	4.841006964	1.599888083	0.115366479	0.93605312	-4.401959045
**TWEAK**	0.166717077	9.02044875	1.525170887	0.132960483	0.93605312	-4.434149817
**LAP TGF-beta**	0.168292923	7.065839643	1.502485743	0.138705749	0.93605312	-4.44366173
**CXCL1**	0.443810308	8.206815893	1.381907581	0.172600819	0.93605312	-4.492130963
**ADA**	0.15885841	5.300381607	1.159171116	0.251406448	0.93605312	-4.57210649
**TGF-alpha**	0.095826205	3.207044286	1.121532945	0.266945606	0.93605312	-4.584356689
**MMP-10**	-0.242798256	8.158806964	-1.114788928	0.269800165	0.93605312	-4.586512283
**STAMBP**	0.37833041	4.095128571	1.09230348	0.27947281	0.93605312	-4.593612396
**IL-20RA**	0.130007539	1.102986964	1.079653995	0.285019549	0.93605312	-4.597547705
**NRTN**	0.157439128	0.702349643	1.049034742	0.29876109	0.93605312	-4.606896947
**CST5**	0.165167897	5.107205357	1.048784219	0.298875365	0.93605312	-4.606972408
**AXIN1**	0.445713795	2.240774821	1.0464616	0.299936252	0.93605312	-4.607671211
**TRANCE**	0.268271897	4.80004375	1.013551654	0.315245583	0.93605312	-4.617417112
**MCP-2**	0.166319744	8.441094286	0.997256945	0.323017727	0.93605312	-4.6221346
**ST1A1**	0.443662564	3.01959125	0.986209653	0.32835942	0.93605312	-4.625292061
**FGF-21**	0.393875436	5.832240714	0.982563537	0.330135275	0.93605312	-4.62632691
**ARTN**	-0.161527282	0.329041607	-0.981201722	0.330800188	0.93605312	-4.626712499
**OSM**	0.226961795	2.062627321	0.977369367	0.332676128	0.93605312	-4.627794903
**CXCL11**	0.279432872	11.28277679	0.975994092	0.333351045	0.93605312	-4.628182361
**CCL19**	0.245839282	9.252349464	0.964183896	0.339184245	0.93605312	-4.631488502
**TRAIL**	0.121022821	7.968634464	0.957926089	0.342302164	0.93605312	-4.633224926
**LIF-R**	0.089029385	3.827790179	0.938754271	0.351971259	0.93605312	-4.638478264
**CD5**	0.132247282	5.06091375	0.921339605	0.360906677	0.93605312	-4.643163012
**MMP-1**	0.337612872	6.99132625	0.892673221	0.375930758	0.93605312	-4.650693315
**CCL28**	-0.129222154	1.570150357	-0.890141369	0.37727652	0.93605312	-4.651347531
**IL-2RB**	0.190629385	1.346601607	0.889110214	0.377825486	0.93605312	-4.651613469
**CXCL9**	0.314363385	7.484497143	0.883523529	0.380808508	0.93605312	-4.653049191
**PD-L1**	0.092079436	6.451255714	0.867233248	0.389591273	0.93605312	-4.657186405
**FGF-19**	-0.383304103	7.483342143	-0.842004198	0.403440826	0.93605312	-4.663448731
**IL13**	0.210356769	0.641963929	0.839011714	0.405103434	0.93605312	-4.664179799
**CXCL10**	0.233202256	9.811040536	0.831522525	0.409282794	0.93605312	-4.665998501
**IL-1 alpha**	-0.093211128	-2.226240714	-0.807498167	0.422866491	0.93605312	-4.671727182
**IL-17C**	-0.17457518	1.719955179	-0.801563024	0.426263647	0.93605312	-4.673117613
**Flt3L**	-0.120332513	9.093605893	-0.775179187	0.441561984	0.93605312	-4.679179205
**IL-17A**	0.166662154	1.857581786	0.756724409	0.45245224	0.93605312	-4.683302992
**IL18**	0.134839949	8.354701964	0.747303419	0.458071212	0.93605312	-4.685371192
**SIRT2**	0.356734564	3.335694107	0.724432301	0.471878326	0.93605312	-4.69028794
**TNFSF14**	-0.120594615	3.725592143	-0.71727793	0.476245291	0.93605312	-4.691795605
**IL2**	-0.067387538	0.64133	-0.714085813	0.47820105	0.93605312	-4.692463617
**LIF**	0.198736615	0.526429286	0.678489407	0.500313233	0.955909446	-4.699717044
**IL-22 RA1**	-0.102637487	2.019938393	-0.655635508	0.514798542	0.955909446	-4.704184008
**VEGFA**	0.055942154	9.906609643	0.648267265	0.519516003	0.955909446	-4.705592434
**CCL4**	0.10292641	5.930994286	0.609119688	0.54495911	0.957920414	-4.71281504
**IL8**	0.117165282	5.141547679	0.60864996	0.545268218	0.957920414	-4.712899037
**IL-12B**	0.134395897	5.687646964	0.568843445	0.571783614	0.957920414	-4.719786927
**DNER**	-0.059045538	8.900324464	-0.553909454	0.581891354	0.957920414	-4.722253316
**4E-BP1**	-0.194009333	6.326274107	-0.527744969	0.599804205	0.957920414	-4.726419184
**uPA**	0.05848159	10.31621375	0.476435086	0.635656355	0.957920414	-4.734012906
**IL33**	-0.054100564	0.8376325	-0.450669912	0.654003418	0.957920414	-4.737537523
**OPG**	-0.062181795	9.794019107	-0.434333626	0.66574952	0.957920414	-4.739672074
**CD6**	0.091178154	5.381283571	0.424379645	0.6729484	0.957920414	-4.740934507
**CASP-8**	-0.10995318	1.554542143	-0.402900251	0.688587245	0.957920414	-4.743560027
**Beta-NGF**	-0.030287333	1.015008036	-0.40168976	0.689472751	0.957920414	-4.743703974
**MCP-3**	-0.072282308	3.714134464	-0.397068634	0.692857238	0.957920414	-4.744249559
**SLAMF1**	0.050256564	1.698056607	0.381892611	0.704016141	0.957920414	-4.7459973
**IL-10RB**	-0.042052359	5.866142143	-0.378897496	0.706226296	0.957920414	-4.746334258
**FGF-23**	0.050122462	2.789065357	0.333603542	0.73995036	0.957920414	-4.75110908
**NT-3**	0.08098759	1.723605893	0.332104976	0.741075393	0.957920414	-4.751256758
**IFN-gamma**	-0.180214154	6.7254475	-0.322899624	0.747998615	0.957920414	-4.752149423
**IL6**	-0.107069026	4.111799821	-0.316718901	0.75265884	0.957920414	-4.752734793
**TNF**	0.05039159	3.287174107	0.299054587	0.766028229	0.957920414	-4.754345768
**CCL3**	0.046287333	5.064526964	0.295973649	0.768367545	0.957920414	-4.754617335
**CD40**	0.044200051	11.35902179	0.285769541	0.77613077	0.957920414	-4.755496793
**CSF-1**	0.02770841	9.846884821	0.274902953	0.784423331	0.957920414	-4.756399596
**CXCL5**	0.132988205	9.866148214	0.268622969	0.789227304	0.957920414	-4.756905459
**HGF**	0.037274923	8.177970714	0.262895272	0.793615991	0.957920414	-4.757356688
**IL-24**	0.018560277	1.5095932	0.071619226	0.943197256	0.986069859	-4.75795521
**MCP-4**	-0.053944513	15.62372411	-0.246819103	0.805969397	0.957920414	-4.758571428
**IL7**	0.062051436	2.17653	0.246000885	0.806599502	0.957920414	-4.758631212
**FGF-5**	0.022278718	0.983330179	0.220792248	0.826073772	0.957920414	-4.760376183
**CCL25**	0.038063333	6.102908929	0.218749401	0.827656924	0.957920414	-4.760509362
**TSLP**	0.042382974	0.52269875	0.211146942	0.833554901	0.957920414	-4.760994143
**MCP-1**	-0.029160513	11.66495339	-0.210518395	0.834042962	0.957920414	-4.761033458
**CCL20**	-0.100633487	7.100073036	-0.198501444	0.843386451	0.957920414	-4.761762623
**GDNF**	0.022790769	1.70909	0.184839768	0.854036261	0.958187025	-4.762539665
**IL-15RA**	0.016521026	1.568929643	0.141000527	0.888385934	0.961136618	-4.764659759
**EN-RAGE**	-0.049448564	2.97878	-0.137401599	0.891216352	0.961136618	-4.764808502
**CCL23**	-0.022150154	9.89340875	-0.124698724	0.90121782	0.961136618	-4.765302801
**CCL11**	0.011253846	7.583721786	0.119128582	0.905608595	0.961136618	-4.765504452
**IL-20**	-0.010332051	0.411957321	-0.114954397	0.908900932	0.961136618	-4.765649532
**CX3CL1**	0.005650564	5.744388214	0.044540034	0.964635649	0.994195919	-4.767317381
**CDCP1**	-0.007047641	3.354912321	-0.03452606	0.972582964	0.994195919	-4.767434987
**IL10**	0.001399487	3.897508393	0.008822627	0.992992655	0.998339444	-4.767600492
**TNFRSF9**	0.00030041	6.918997143	0.002090704	0.998339444	0.998339444	-4.767611405

End of treatment: Lifestyle vs. Liraglutide						
	logFC	***AveExpr***	***t***	***P.Value***	***adj.P.Val***	***B***
CD8A	0.510934154	10.18864161	2.328142733	0.023618601	0.940382973	-4.025076364
MMP-10	-0.494151539	8.158806964	-2.268857577	0.027231856	0.940382973	-4.059645509
IL-18R1	-0.302773949	8.568600536	-2.218677798	0.030664662	0.940382973	-4.08841371
CXCL6	0.271195692	9.260345357	1.799689601	0.077408957	0.98258344	-4.309581062
CCL19	0.446872974	9.252349464	1.752639862	0.085250192	0.98258344	-4.33213916
IL5	0.669006769	1.071607679	1.727087185	0.089778333	0.98258344	-4.344185707
IL4	0.288914308	0.71673125	1.672984947	0.100023401	0.98258344	-4.369209111
IL-17C	0.364250718	1.719955179	1.672459439	0.100127428	0.98258344	-4.369448925
IL-10RA	-0.753113077	1.419014821	-1.661701485	0.102276572	0.98258344	-4.374344491
LAP TGF-beta	0.179946769	7.065839643	1.606528964	0.11389824	0.98258344	-4.399034618
IL-17A	0.349457744	1.857581786	1.58670219	0.118327073	0.98258344	-4.407735245
NRTN	0.220038308	0.702349643	1.466140165	0.148318174	0.98258344	-4.458644058
VEGFA	0.125910051	9.906609643	1.45906725	0.150248181	0.98258344	-4.461522564
CST5	0.224863385	5.107205357	1.427839023	0.159005579	0.98258344	-4.474086252
CCL28	-0.161876	1.570150357	-1.115076014	0.269678212	0.98258344	-4.586420767
CXCL1	0.35676041	8.206815893	1.110857289	0.271474215	0.98258344	-4.587763412
TSLP	0.216715744	0.52269875	1.079652084	0.285020392	0.98258344	-4.597548296
IL-12B	0.250492256	5.687646964	1.060232349	0.293683917	0.98258344	-4.603506926
TNFB	0.176224769	4.841006964	1.059168939	0.294163513	0.98258344	-4.60383031
ARTN	-0.162215641	0.329041607	-0.985383176	0.3287614	0.98258344	-4.625526949
OSM	0.223512308	2.062627321	0.962514783	0.340014035	0.98258344	-4.631952692
CSF-1	0.092291744	9.846884821	0.915652419	0.363856163	0.98258344	-4.644674924
TGF-alpha	0.076801897	3.207044286	0.898875815	0.372646766	0.98258344	-4.649083137
uPA	0.109816718	10.31621375	0.894649712	0.374882308	0.98258344	-4.650181374
LIF	0.25192718	0.526429286	0.860082689	0.393486106	0.98258344	-4.658979233
TWEAK	0.092813846	9.02044875	0.849085041	0.399523506	0.98258344	-4.661708961
IL7	0.204138103	2.17653	0.80929882	0.421839064	0.98258344	-4.671303395
CD5	0.109821692	5.06091375	0.765105135	0.447487512	0.98258344	-4.681442158
Beta-NGF	-0.057658667	1.015008036	-0.764705685	0.447723421	0.98258344	-4.6815313
NT-3	0.186109897	1.723605893	0.763178942	0.448625761	0.98258344	-4.681871594
MCP-2	0.119625077	8.441094286	0.717274668	0.476247288	0.98258344	-4.691796289
MCP-3	0.12488441	3.714134464	0.686027934	0.49558431	0.98258344	-4.698210966
LIF-R	0.062307692	3.827790179	0.656992212	0.513932423	0.98258344	-4.703922988
FGF-19	-0.293199487	7.483342143	-0.644071371	0.522212601	0.98258344	-4.706387543
OPG	-0.089358974	9.794019107	-0.62416351	0.535106924	0.98258344	-4.710091416
EN-RAGE	0.222663744	2.97878	0.618710673	0.538667297	0.98258344	-4.711086127
CCL20	0.306285436	7.100073036	0.604153775	0.548231487	0.98258344	-4.713699848
IL-10RB	-0.066305385	5.866142143	-0.597420569	0.55268433	0.98258344	-4.714888237
TNFSF14	-0.097210103	3.725592143	-0.578190502	0.565501207	0.98258344	-4.718210523
CD6	-0.121921744	5.381283571	-0.567472624	0.572707839	0.98258344	-4.720016003
GDNF	-0.069290872	1.70909	-0.561969126	0.576425685	0.98258344	-4.72093023
SCF	-0.076068872	8.777206964	-0.558994383	0.578440109	0.98258344	-4.72142075
IL-22 RA1	-0.085591692	2.019938393	-0.54674909	0.586767937	0.98258344	-4.723413041
IL-20RA	-0.06429641	1.102986964	-0.5339527	0.595531079	0.98258344	-4.725448708
SIRT2	-0.253360462	3.335694107	-0.514507201	0.608963793	0.98258344	-4.728451426
Flt3L	-0.079179231	9.093605893	-0.510070723	0.612047769	0.98258344	-4.729121149
TNFRSF9	-0.071896308	6.918997143	-0.500362017	0.618821324	0.98258344	-4.730566845
CD244	0.078688154	7.01331375	0.477984477	0.634560193	0.98258344	-4.733794794
IFN-gamma	-0.253156205	6.7254475	-0.453593914	0.651910154	0.98258344	-4.737147252
MCP-1	-0.062814718	11.66495339	-0.453478089	0.651993019	0.98258344	-4.737162759
IL2	-0.042349487	0.64133	-0.44876499	0.65536864	0.98258344	-4.737790435
IL13	-0.110056154	0.641963929	-0.438960926	0.662413676	0.98258344	-4.739075359
IL33	-0.05229441	0.8376325	-0.435624243	0.664818421	0.98258344	-4.739506271
FGF-23	-0.065297128	2.789065357	-0.434602623	0.665555412	0.98258344	-4.739637557
IL-1 alpha	-0.050121385	-2.226240714	-0.43420702	0.665840887	0.98258344	-4.739688313
IL8	0.082807795	5.141547679	0.430169758	0.668757096	0.98258344	-4.740203685
MMP-1	0.162549333	6.99132625	0.429792372	0.669029954	0.98258344	-4.740251617
FGF-5	0.043362564	0.983330179	0.429742772	0.669065819	0.98258344	-4.740257913
SLAMF1	-0.054861231	1.698056607	-0.41688283	0.678390669	0.98258344	-4.741866198
AXIN1	-0.175642256	2.240774821	-0.412378703	0.681668745	0.98258344	-4.742418064
FGF-21	0.165057487	5.832240714	0.411753193	0.682124478	0.98258344	-4.742494235
CASP-8	-0.111149795	1.554542143	-0.407284996	0.685383365	0.98258344	-4.74303502
TRANCE	0.101451231	4.80004375	0.383290474	0.702985507	0.98258344	-4.745839136
CXCL10	-0.101898923	9.811040536	-0.363338036	0.71774856	0.98258344	-4.748042416
HGF	0.051354205	8.177970714	0.36219465	0.71859792	0.98258344	-4.748165139
CXCL9	0.128486872	7.484497143	0.361114493	0.719400638	0.98258344	-4.748280722
CXCL5	0.17260718	9.866148214	0.348649363	0.72868691	0.98258344	-4.749589789
TRAIL	-0.036606974	7.968634464	-0.289753417	0.773097077	0.98258344	-4.755157088
CCL25	0.048798718	6.102908929	0.280445495	0.780190472	0.98258344	-4.755943469
CD40	-0.043372051	11.35902179	-0.280416217	0.780212813	0.98258344	-4.755945903
IL-24	-0.025841458	1.5095932	-0.099715391	0.920978237	0.98258344	-4.757270093
ST1A1	-0.113529282	3.01959125	-0.25236223	0.801704097	0.98258344	-4.758161201
IL18	0.037465026	8.354701964	0.207636847	0.836281301	0.98258344	-4.761212199
CX3CL1	-0.025010256	5.744388214	-0.197140968	0.844445711	0.98258344	-4.76184248
MCP-4	-0.040527949	15.62372411	-0.185432613	0.853573533	0.98258344	-4.762507093
IL-2RB	0.033202872	1.346601607	0.154860766	0.877499365	0.98258344	-4.764051043
CCL23	0.026717385	9.89340875	0.150410863	0.880992065	0.98258344	-4.764252682
DNER	-0.013696154	8.900324464	-0.128484375	0.898235468	0.98258344	-4.765160499
IL-20	-0.010945026	0.411957321	-0.121774349	0.903522638	0.98258344	-4.765409817
CCL11	0.011396923	7.583721786	0.120643135	0.904414419	0.98258344	-4.765450533
IL-15RA	-0.012603795	1.568929643	-0.107568486	0.914730383	0.98258344	-4.765893574
PD-L1	0.008856769	6.451255714	0.083415854	0.93382448	0.98258344	-4.766578572
CCL4	-0.013960872	5.930994286	-0.082620601	0.934453903	0.98258344	-4.766598182
IL10	0.009113897	3.897508393	0.057455702	0.954390929	0.98258344	-4.767121715
4E-BP1	-0.020192564	6.326274107	-0.054927894	0.956395434	0.98258344	-4.76716391
CDCP1	-0.009541231	3.354912321	-0.046742038	0.962888545	0.98258344	-4.767287525
TNF	0.007837897	3.287174107	0.046514889	0.963068761	0.98258344	-4.767290672
IL6	0.014750205	4.111799821	0.043632309	0.965355904	0.98258344	-4.767329269
CXCL11	0.011219538	11.28277679	0.039187241	0.968883349	0.98258344	-4.767383951
ADA	0.005197436	5.300381607	0.037925078	0.969885073	0.98258344	-4.767398408
CCL3	0.005354872	5.064526964	0.034240489	0.972809644	0.98258344	-4.767437904
STAMBP	-0.007595641	4.095128571	-0.021929892	0.98258344	0.98258344	-4.767540617

12-weeks post treatment: Lifestyle vs. Liraglutide						
	logFC	AveExpr	t	P.Value	adj.P.Val	B
IL-17A	0.471057968	1.844296122	2.333822404	0.023859744	0.977040695	-4.064791253
CCL19	0.481423928	9.220363061	2.121321365	0.039109884	0.977040695	-4.175592324
FGF-23	0.387634145	2.839308776	2.063503683	0.044520308	0.977040695	-4.204474174
IL4	0.394283889	0.697237347	2.03669246	0.047242999	0.977040695	-4.217673957
TSLP	0.358197486	0.442172653	1.739135313	0.088450996	0.977040695	-4.355427241
OSM	0.58913242	2.089782449	1.677517413	0.099972522	0.977040695	-4.381831232
CSF-1	0.159133843	9.850923469	1.610724955	0.113827452	0.977040695	-4.409581287
IL-10RA	-0.738251001	1.290724286	-1.521707982	0.134673723	0.977040695	-4.44511691
IL-12B	0.349743037	5.679049388	1.516455314	0.135993316	0.977040695	-4.447161071
LAP TGF-beta	0.172606953	7.096401429	1.497233011	0.140910393	0.977040695	-4.454590995
CST5	0.250451857	5.13254	1.468936429	0.148403977	0.977040695	-4.465382458
CD8A	0.32540803	10.21625796	1.409530498	0.165154605	0.977040695	-4.487465955
CD5	0.193362503	5.063001633	1.307497697	0.197300838	0.977040695	-4.523549456
IL5	0.496214969	0.912567551	1.267650906	0.211066019	0.977040695	-4.536994337
IL-22 RA1	-0.240328644	1.972944286	-1.262550449	0.212878514	0.977040695	-4.538688697
VEGFA	0.127428467	9.917119592	1.246384881	0.218699673	0.977040695	-4.544018729
TGF-alpha	0.099727171	3.237135918	1.223642965	0.227087456	0.977040695	-4.551413363
TNFB	0.211064688	4.854867551	1.189251994	0.24021743	0.977040695	-4.562363866
TWEAK	0.13200422	9.070287755	1.153304521	0.254522586	0.977040695	-4.573509096
CXCL6	0.173714594	9.32710551	1.124192556	0.266547934	0.977040695	-4.582307688
IL-10RB	0.119585975	5.888918776	1.099794403	0.276932446	0.977040695	-4.589523712
TNF	0.156689154	3.250492653	1.063512597	0.29289537	0.977040695	-4.599986215
NRTN	0.165672015	0.636322653	1.047226665	0.300264113	0.977040695	-4.604577598
Flt3L	0.163538305	9.137382653	1.032705976	0.306940819	0.977040695	-4.608616187
uPA	0.123517009	10.33436694	0.996783638	0.323891266	0.977040695	-4.618382686
CDCP1	0.209216833	3.264922653	0.989898667	0.327210582	0.977040695	-4.620217894
IL13	0.391516107	0.720214082	0.976912045	0.333533383	0.977040695	-4.623647277
CX3CL1	0.137107257	5.796115714	0.954303787	0.34473344	0.977040695	-4.629516615
SCF	-0.132972021	8.815671837	-0.952426261	0.345674569	0.977040695	-4.629998265
CASP-8	0.205880198	1.525351633	0.922341777	0.360984744	0.977040695	-4.637594694
ADA	0.115907464	5.263135918	0.760342958	0.450785629	0.977040695	-4.674524943
IL10	-0.140106003	3.928242449	-0.757150891	0.452677427	0.977040695	-4.675184446
Beta-NGF	-0.063470929	1.025756939	-0.748621607	0.457754997	0.977040695	-4.676933597
HGF	0.1109495	8.164302857	0.728563042	0.469825142	0.977040695	-4.680972096
IL-18R1	-0.105557397	8.550617755	-0.718923817	0.475689463	0.977040695	-4.682875281
CXCL9	0.204235702	7.433492041	0.710355513	0.480936842	0.977040695	-4.684546526
IFN-gamma	0.309732229	6.591345714	0.709861765	0.48124021	0.977040695	-4.684642243
IL-15RA	0.078661766	1.56463	0.697688109	0.488753823	0.977040695	-4.686981901
IL18	0.133322995	8.297866735	0.692437748	0.492014383	0.977040695	-4.687978914
CXCL5	-0.430837954	9.840097755	-0.679832586	0.499891314	0.977040695	-4.690342858
IL-1 alpha	0.100986222	-2.24214449	0.657842963	0.513796269	0.977040695	-4.694366149
ST1A1	0.384632525	2.928964286	0.647848744	0.520184015	0.977040695	-4.696152361
CD40	-0.123112053	11.37379959	-0.631129897	0.530963468	0.977040695	-4.699081106
TNFRSF9	0.095596287	6.928335918	0.618813357	0.538978703	0.977040695	-4.701191082
LIF-R	0.052974824	3.853683061	0.610964177	0.544119164	0.977040695	-4.702514645
MCP-2	0.101753923	8.474095714	0.609100448	0.545343412	0.977040695	-4.7028265
EN-RAGE	-0.155502398	3.019187143	-0.561958435	0.576771307	0.977040695	-4.710405679
FGF-21	-0.25269717	5.744727143	-0.555469639	0.581165233	0.977040695	-4.711402245
AXIN1	-0.295696877	2.15452551	-0.546693418	0.587133682	0.977040695	-4.712732112
MMP-10	0.136469905	8.221773878	0.545059261	0.58824825	0.977040695	-4.712977449
TRAIL	0.072022753	7.978062245	0.541804156	0.590471379	0.977040695	-4.713463997
CXCL11	0.135730883	11.2664	0.514580738	0.609218362	0.977040695	-4.717421333
ARTN	-0.086888777	0.306957755	-0.506007276	0.615178429	0.977040695	-4.718626197
CCL3	0.067864752	5.030945306	0.474352814	0.637409226	0.977040695	-4.722902443
IL-20RA	-0.067057796	1.078230816	-0.474296841	0.637448844	0.977040695	-4.722909764
TRANCE	0.132130287	4.752934898	0.461579008	0.646477985	0.977040695	-4.724551147
FGF-5	0.053696069	1.001554082	0.461040841	0.646861261	0.977040695	-4.724619635
CCL20	0.175779831	6.914081633	0.432799108	0.667108211	0.977040695	-4.728103233
CCL11	0.041931682	7.632733469	0.431392222	0.668123562	0.977040695	-4.728271096
CCL25	-0.073323872	6.151912857	-0.385950895	0.701246334	0.977040695	-4.73340254
IL-20	-0.031043607	0.38294	-0.38488222	0.702032715	0.977040695	-4.733516431
SLAMF1	0.051445133	1.623068367	0.361441918	0.719362638	0.977040695	-4.735935875
SIRT2	-0.202861511	3.255158163	-0.339734231	0.735545678	0.977040695	-4.738042182
IL-24	0.059489986	1.495012667	0.253292232	0.801228126	0.977040695	-4.738522467
GDNF	0.047653211	1.667241837	0.331161259	0.7419708	0.977040695	-4.7388384
CXCL10	0.081170902	9.763319184	0.312504017	0.756017435	0.977040695	-4.7405014
IL2	-0.031561681	0.621940816	-0.304277163	0.762238109	0.977040695	-4.741204283
TNFSF14	0.059489049	3.670327347	0.291207061	0.77215348	0.977040695	-4.742282655
CCL4	0.044486015	5.908803469	0.284577641	0.777197587	0.977040695	-4.742811643
PD-L1	0.031822715	6.465767143	0.278712479	0.781668294	0.977040695	-4.743269551
CCL28	-0.039233467	1.58199551	-0.273785074	0.785429967	0.977040695	-4.743646917
CXCL1	-0.1036021	8.227875306	-0.270290112	0.788101229	0.977040695	-4.74391052
CD244	0.039214797	7.003618776	0.263340926	0.793420232	0.977040695	-4.744424645
LIF	0.073975072	0.48864898	0.25069711	0.803123289	0.977040695	-4.745325899
IL8	0.049893141	5.177640408	0.249707724	0.803883903	0.977040695	-4.745394561
IL-2RB	0.051666313	1.330170408	0.232408243	0.817213628	0.977040695	-4.746551425
4E-BP1	-0.068232162	6.290417551	-0.222675919	0.824736968	0.977040695	-4.747165903
MCP-4	-0.045928757	15.67139531	-0.215847821	0.830025243	0.977040695	-4.747581382
IL-17C	-0.05941653	1.737489592	-0.204149419	0.839103865	0.977040695	-4.748263236
CCL23	-0.033915125	9.956077347	-0.185004432	0.854008672	0.977040695	-4.749297405
IL6	0.053172643	3.987925714	0.165727256	0.869070892	0.977040695	-4.750236145
IL7	-0.05111724	2.285793878	-0.1396481	0.889525063	0.977040695	-4.751342182
IL33	-0.019391586	0.856199592	-0.132675974	0.895006856	0.977040695	-4.751605919
FGF-19	-0.051104287	7.733140612	-0.115662244	0.908404998	0.977040695	-4.752192882
NT-3	0.033729956	1.662924286	0.105722701	0.916245003	0.977040695	-4.752498609
MMP-1	0.037685043	7.034041224	0.097978099	0.922359579	0.977040695	-4.752717808
DNER	0.010185442	8.930449796	0.095976531	0.923940657	0.977040695	-4.752771751
OPG	0.008008154	9.826165918	0.057980882	0.954005679	0.978754104	-4.753584669
MCP-3	-0.009154793	3.728226531	-0.042182733	0.966528821	0.978754104	-4.753804601
CD6	-0.009264253	5.380725102	-0.04197243	0.966695593	0.978754104	-4.75380706
STAMBP	-0.015893517	4.020209592	-0.040181997	0.968115473	0.978754104	-4.753827505
MCP-1	-0.002570947	11.7322298	-0.018282138	0.985489912	0.985489912	-4.754005467

4. Supplementary references

[1] Purvis LAB, Clarke WT, Biasiolli L, Valkovič L, Robson MD, Rodgers CT. OXSA: An open-source magnetic resonance spectroscopy analysis toolbox in MATLAB. PLoS One 2017;12:e0185356.

[2] Piechnik SK, Ferreira VM, Dall'Armellina E, Cochlin LE, Greiser A, Neubauer S, et al. Shortened Modified Look-Locker Inversion recovery (ShMOLLI) for clinical myocardial T1-mapping at 1.5 and 3 T within a 9 heartbeat breathhold. J Cardiovasc Magn Reson 2010;12:69.

[3] Andersson A, Kelly M, Imajo K, Nakajima A, Fallowfield JA, Hirschfield G, et al. Clinical Utility of Magnetic Resonance Imaging Biomarkers for Identifying Nonalcoholic Steatohepatitis Patients at High Risk of Progression: A Multicenter Pooled Data and Meta-Analysis. Clin Gastroenterol Hepatol 2022;20:2451-2461.e2453.

[4] Jayaswal ANA, Levick C, Selvaraj EA, Dennis A, Booth JC, Collier J, et al. Prognostic value of multiparametric magnetic resonance imaging, transient elastography and blood-based fibrosis markers in patients with chronic liver disease. Liver Int 2020;40:3071-3082.
